# A metabarcoding framework for facilitated survey of endolithic phototrophs with *tuf*A

**DOI:** 10.1186/s12898-016-0068-x

**Published:** 2016-03-10

**Authors:** Thomas Sauvage, William E. Schmidt, Shoichiro Suda, Suzanne Fredericq

**Affiliations:** Department of Biology, University of Louisiana at Lafayette, Lafayette, LA 70503 USA; Department of Marine Science, Biology and Chemistry, University of the Ryukyus, Nishihara, Okinawa 903-0213 Japan

**Keywords:** Algae, Amplicon Metagenomics, Bryopsidales, Calcium carbonate, Coral reef, Illumina MiSeq, Metabarcoding, Next generation sequencing, *Ostreobium*, *Phaeophila*, Phototrophs, Plastid, Rhodoliths, *tuf*A, Ulvophyceae

## Abstract

**Background:**

In spite of their ecological importance as primary producers and microbioeroders of marine calcium carbonate (CaCO_3_) substrata, endolithic phototrophs spanning both prokaryotic (the cyanobacteria) and eukaryotic algae lack established molecular resources for their facilitated survey with high throughput sequencing. Here, the development of a metabarcoding framework for the elongation factor EF-T*tu* (*tuf*A) was tested on four Illumina-sequenced marine CaCO_3_ microfloras for the characterization of their endolithic phototrophs, especially the abundant bioeroding *Ostreobium* spp. (Ulvophyceae). The framework consists of novel *tuf*A degenerate primers and a comprehensive database enabling Operational Taxonomic Unit (OTU) identification at multiple taxonomic ranks with percent identity thresholds determined herein.

**Results:**

The newly established *tuf*A database comprises 4057 non-redundant sequences (from 1339 eukaryotic and prokaryotic phototrophs, and 2718 prokaryotic heterotrophs) including 27 classes in 10 phyla of phototrophic diversity summarized from data mining on GenBank^®^, our barcoding of >150 clones produced from coral reef microfloras, and >300 eukaryotic phototrophs (>230 Ulvophyceae including >100 ‘*Ostreobium*’ spp., and >70 Florideophyceae, Phaeophyceae and miscellaneous taxa). Illumina metabarcoding with the newly designed primers resulted in 802 robust OTUs including 618 phototrophs and 184 heterotrophs (77 and 23 % of OTUs, respectively). Phototrophic OTUs belonged to 14 classes of phototrophs found in seven phyla, and represented ~98 % of all reads. The phylogenetic profiles of coral reef microfloras showed few OTUs in large abundance (proportion of reads) for the Chlorophyta (Ulvophyceae, i.e. *Ostreobium* and *Phaeophila*), the Rhodophyta (Florideophyceae) and Haptophyta (Coccolithophyceae), and a large diversity (richness) of OTUs in lower abundance for the Cyanophyta (Cyanophyceae) and the Ochrophyta (the diatoms, ‘Bacillariophyta’). The bioerosive ‘*Ostreobium*’ spp. represented four families in a large clade of subordinal divergence, i.e. the Ostreobidineae, and a fifth, phylogenetically remote family in the suborder Halimedineae (provisionally assigned as the ‘Pseudostreobiaceae’). Together they harbor 85–95 delimited cryptic species of endolithic microsiphons.

**Conclusions:**

The novel degenerate primers allowed for amplification of endolithic phototrophs across a wide phylogenetic breadth as well as their recovery in very large proportions of reads (overall 98 %) and diversity (overall 77 % of OTUs). The established companion *tuf*A database and determined identity thresholds allow for OTU identification at multiple taxonomic ranks to facilitate the monitoring of phototrophic assemblages via metabarcoding, especially endolithic communities rich in bioeroding Ulvophyceae, such as those harboring ‘*Ostreobium*’ spp., *Phaeophila* spp. and associated algal diversity.

**Electronic supplementary material:**

The online version of this article (doi:10.1186/s12898-016-0068-x) contains supplementary material, which is available to authorized users.

## Background

Endolithic phototrophs are major primary producers [68] and microbioeroders in marine carbonate substrata [98]. They may colonize the surface and cavities of the substratum (chaesmoendoliths and cryptoendoliths, respectively), as well as actively penetrate it (euendoliths) wherever sufficient light penetrates for photosynthesis. They may be found in aragonite, calcite, or a combination of both, in e.g. live and dead corals, mollusk shells, and crustose coralline algae (CCA) [97]. In calcium carbonate CaCO_3_-building ecosystems, such as CCA ridges, coral reefs, oyster reefs and rhodolith beds, euendolithic phototrophs play a critical role in the dynamic balance between constructive (accretion) and destructive (dissolution) processes [36]. With upcoming global changes, this balance may be negatively affected by enhancing the bioerosive power of boring phototrophs, as measured under projected ocean acidification [higher partial pressure of carbon dioxide (pCO_2_)] and increased temperature regimes [81, 82, 99]. The prospect of accelerated biogenic CaCO_3_ dissolution poses strong concerns for the future maintenance of the structural integrity and functionality of these ecosystems considering the biodiverse assemblages of micro- and macro-organisms they support [72, 73].

Commonly reported euendolithic phototrophs include the eukaryotic green algal genera *Ostreobium* Bornet and Flahault (order Bryopsidales) and *Phaeophila* Hauck (‘Ulvales-Ulothrichales’) (both in class Ulvophyceae, phylum Chlorophyta), and prokaryotic (eubacterial) blue-green algal genera (all in class Cyanophyceae, phylum Cyanophyta) such as *Mastigocoleus* Lagerheim ex Bornet and Flahault, and *Plectonema* Thuret ex Gomont [98]. They also include microscopic alternate life stages of otherwise conspicuous alga, e.g. the *Conchocelis*-stage of the red alga *Porphyra* C. Agardh (Bangiophyceae, Rhodophyta) and endolithic vegetative networks underlying diminutive epilithic Bryopsidales, e.g. *Pseudochlorodesmis* Børgesen and *Caulerpa ambigua* Okamura [1, 50]. Among the above, *Ostreobium* microsiphons are omnipresent agents of bioerosion [98], although the microfilaments of the genus *Phaeophila* are also often reported (e.g. see [12, 79]). The molecular diversity of *Ostreobium* spp. remains particularly unexplored toward establishing comprehensive sequence reference databases for the profiling of endolithic communities via metabacording (e.g. [14]).

**Fig. 1:**
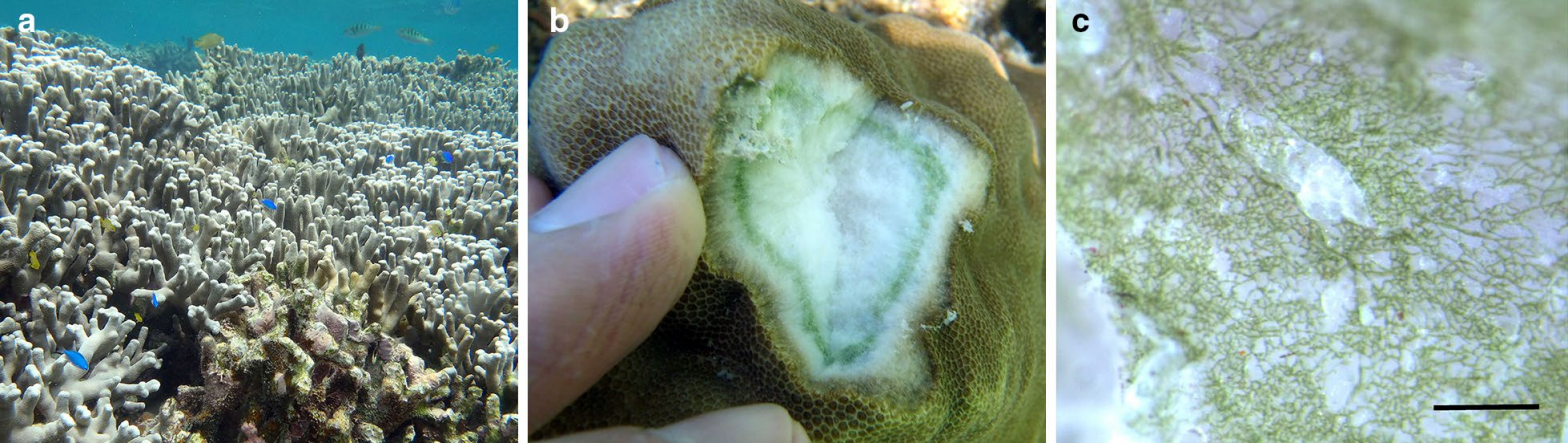
*Ostreobium* in situ in the Ryukus. **a** Coral reef habitat where microfloras harboring *Ostreobium* spp. underlay live coral tissue (*picture background*) and limestone often covered by epilithic turf algae and crustose coralline algae (*foreground*). **b** Fragmented coral colony showing *Ostreobium*’s ‘*green layer*’ found below live coral tissue, here *Porites* sp. **c** Calcium carbonate colonized by *Ostreobium* sp. microsiphons (*Scale bar* approximately 20 µm)

The skeleton of reef-building scleractian coral species (Cnidaria) is abundantly colonized by *Ostreobium* [54, 60, 61] (Fig. 1), in which it develops a dense green layer underlying the animal tissue and where, as part of the coral holobiont, it may play a role as a nutritional ally (i.e. metabolite translocation, [31]). Previously, Gutner-Hoch and Fine [38] investigated the molecular diversity of this ‘green layer’ with *rbc*L in two coral species from the Red Sea in order to gain insights into potential patterns of association of *Ostreobium* haplotypes with coral species. While these authors reported some possible haplotype-to-coral species distributional patterns, they also revealed multiple *rbc*L haplotypes of *Ostreobium,* whose taxonomic breadth remains unknown (with regard to species diversity and the taxonomic rank attributed to this novel diversity) from a lack of phylogenetic context in their analysis. Earlier, Verbruggen et al. [102] suggested that the genus *Ostreobium* might actually represent an entire suborder of microsiphonous species that they informally proposed as the ‘Ostreobidineae’ (next to two other informally accepted suborders in the Bryopsidales, the Bryopsidineae and Halimedineae, see [41]) based on the early branching of a single *Ostreobium* specimen in a comprehensive multi-marker phylogeny of the Bryopsidales [Chloroplast 16S ribosomal DNA (rDNA), RuBisCO Large subunit (*rbc*L), and elongation EF-*Tu* (*tuf*A)]; however, this remains to be substantiated. Overall, considering the high density of *Ostreobium* microsiphons in the so-called coral ‘green layer’, this microhabitat is particularly convenient to target in order to rapidly build reference barcode data sets.

Metabarcoding represents a novel terminology [96] for amplicon-based metagenomics (e.g. usually targeting 16S, [18]) in contrast to whole-genome metagenomics [65]. Important limitations to metabarcoding include the availability of universal primers amplifying the targeted diversity with minimal taxon bias [51, 59], the clustering of next-generation reads into biologically relevant Operational Taxonomic Unit (OTU) through the elimination of sequencing error/noise (e.g. [25, 26, 88]), and building up taxonomy-curated reference sequence database for OTU identification/annotation (e.g. Greengenes, [21]; Ribosomal Database Project [RDP], [13]; silva, [77]). Currently, a metabarcoding framework specifically developed to target lower phototrophs (i.e. the algae sensu lato: prokaryotic blue-green algae and eukaryotic algae) and their recovery in large proportions of reads is inexistent and is sorely needed to facilitate the monitoring of endolithic phototrophs and other microbial/algal assemblages. A recent progress toward metabarcoding phototrophs was made with the establishment of a curated chloroplast 16S rDNA database (primarily for phytoplankton taxa, PHYTO-REF, [17]); however, primers used to amplify 16S are generally highly conserved in prokaryotes and tend to recover low proportions of phototrophic organisms from environmental mixtures where microbial DNA from heterotrophic phyla is inherently overdominant. For instance, in a study of freshwater phytoplankton communities with 16S (V3–V4 region), only 9 % of reads represented phototrophic organisms [27]. Likewise, 16S libraries sequenced from coral tissue and their underlying endolithic communities enumerated very few phototrophs in comparison to heterotrophs (e.g. see [59, 85, 94]).

A candidate DNA marker for metabarcoding phototrophs is the gene encoding the protein chain elongation factor EF-*Tu*, or *tuf*A, whose role in the RNA translation machinery is deeply conserved among eubacteria and their eukaryotic endosymbiotic offshoot, the organelles [52], especially in the chloroplast (translation mechanisms are modified in the mitochondrion, see [95]). Iwabe et al. [42] first used the elongation factors EF-*Tu* (and its homolog in archaea and eukaryotic nucleus, the elongation factor 1 alpha, EF-1a, [40]) to examine deep (domain) phylogenetic relationships (EF-*Tu* and EF-1a show some amino acid conservation but their DNA sequences are highly divergent). Later, Delwiche et al. [19] demonstrated the cyanobacterial (Cyanophyta) origin of all plastids in a single-gene phylogeny of *tuf*A rooted with heterotrophs. Subsequently, *tuf*A has gained much popularity in phylogenetic and systematic studies of diverse phototrophs (e.g. [5, 29, 66, 84, 106]) and was also recommended as a standard marker for the routine barcoding of the Chlorophyta [86] for its high amplification rate (95 %, except in the Cladophorales, Ulvophyceae), and faster evolving rate relative to other commonly used markers in this phylum [e.g. *rbc*L or rDNA markers such as the large subunit (LSU), 23S universal plastid amplicon (UPA) and the internal transcribed spacer (ITS)]. In early branching members of the Streptophyta (the Charophytes *Mesostigma* and *Chlorokybus*), *tuf*A is chloroplast-encoded while in the remainder of this phylum (i.e. the higher phototrophs), it is nuclear-encoded [3]. In some eubacteria (Gram-negative), including some Cyanophyta, e.g. in the Oscillatoriales (e.g. *Oscillatoria nigro*-*viridis* PCC7112), *tuf*A paralogs known as elongation factor EF-*Tu* 2 or *tuf*B may exist [45, 53, 90]; however, these usually undergo concerted evolution and thus exhibit very low divergence [100]. Considering the universality of *tuf*A and the large amount of data available on Genbank^®^ for the order Bryopsidales (>1300 accessions), this marker is thus well-suited for the barcoding of *Ostreobium* spp. and metabarcoding of microfloras dominated by its microsiphons and associated algal diversity.

Here, we established a metabacording framework consisting of newly developed primers, a curated database of phototrophic diversity (GenBank^®^ data, a clone library of endolithic phototrophs, and new reference barcodes for *Ostreobium* spp. and related taxa), a provisional classification scheme for cryptic endoliths in the order Bryopsidales, and recommended identity thresholds for the taxonomic annotation of phototrophs at high levels (domain to class) and at lower levels in the Ulvophyceae (order to family, i.e. in the Bryopsidales and Ulvales). This framework, geared toward molecular ecology studies of endolithic phototroph assemblages rich in bioeroding Ulvophycean taxa (e.g. *Ostreobium* spp.), is tested on four Illumina-metabarcoded CaCO_3_ microfloras.

## Methods

### Building up *Ostreobium* and reference *tuf*A diversity

**Fig. 2:**
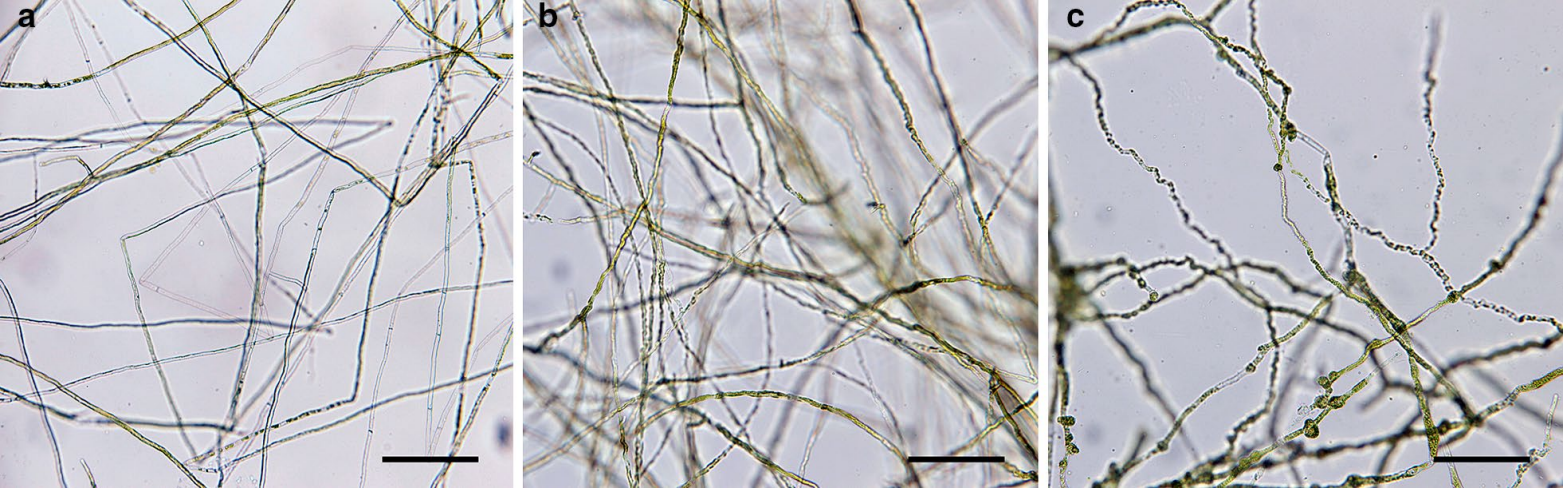
Free-living *Ostreobium* in culture. **a** Specimen belonging in provisional family ‘Hamidaceae’ (TS1385, note that some chloroplast-depleted siphons may falsely appear septated), **b** ‘Maedaceae’ (TS1410B), and **c** ‘Odoaceae’ (TS1408). No picture is available for the ‘Unarizakiaceae’. *Scale bar* 50 µm

*Ostreobium* specimens were sequenced primarily from the CaCO_3_ of densely colonized coral skeletons collected throughout the Ryukyu archipelago and culture starters established from them (Fig. 2, Additional file 1: Table S1). A few additional specimens originated from endolithic siphons underlying rhodolith-forming encrusting red algae from the Gulf of Mexico (GM) and also from starters established from oyster shells collected in Florida and corals from miscellaneous localities (Additional file 1: Table S1). For culturing, colonized blocks of CaCO_3_ were subsetted to ~0.04–0.25 cm^2^ and incubated for 10–20 days in 60 mL polypropylene cups (Diamond™) with 20–30 mL half strength Provasoli enriched seawater medium [75] supplemented with 1.25 mg/L germanium dioxide (GeO_2_) under light–dark 14:10 cycles (50 μmol m^−2^ s^−1^) and at room temperature (22 ± 1 °C). Specimens were extracted with a DNeasy Plant Mini Kit (Qiagen, Valencia, CA, USA) and *tuf*A amplified by polymerase chain reaction (PCR) as previously published [29, 39, 87] and/or with novel primers in various combination (Table 1). Successful PCR products were Sanger-sequenced commercially and chromatograms assembled in Sequencher v.5.1 (Gene Codes, Ann Arbor, Michigan, USA). To further increase reference sequence context, *tuf*A barcodes were also generated for macroscopic (>5–20 cm, e.g. *Codium, Halimeda*, *Rhipilia*) and diminutive members of the Bryopsidales (<2 cm) (e.g. the polyphyletic ‘*Pseudochlorodesmis*’ species complex, [103]; and the monophyletic *Caulerpa ‘ambigua’* species complex, [23]). Likewise, miscellaneous ‘Ulvales-Ulothrichales’ that occasionally emerged in cultures (Additional file 1: Table S1) as well as several Florideophyceae (Rhodophyta) and few Phaeophyceae (Ochrophyta) maintained in the algal collections at the University of Louisiana at Lafayette (LAF) were also barcoded (Additional file 2: Table S2). Corals and reef substratum collections in the Ryukyu archipelago were conducted under Permit 24-3 delivered by the Okinawa Prefecture Fishery Control 33-2-40. Collections in Florida’s coastal waters were permitted by a ‘Saltwater fishing’ license from the Fish and Wildlife (#1000427446) and those from Garden Key, Dry Tortugas by the U.S. National Park Service (#DRTO-2013-SCI-0015).

**Table 1:** Newly designed PCR primers for *tuf*A barcoding and metabarcoding

Name	Sequence (5′–3′)	bp	GC ( %)	Tm ( °C)
*tu*470F	TTTTAATGGCTGTCGAAAATGTTG	24	33.3	52.8
*tu*bryoF	GCAGATGGTCCAATGCCWCAAAC	23	52.2	59
*tu*bryoR	CCWGGTTTAGCTAAAACCATNCC	23	45.7	54.9
*env_tufA*F^a^	TGGGTDGAHAADATTTWYNMNYTRATGR	28	33.3	46.5–62.8
*env_tufA*R^a^	TNACATCHGTWGTWCKNACATARAAYTG	28	35.1	49.8–60.6

^a^ Metabarcoding primers

### Microflora samples

**Fig. 3:**
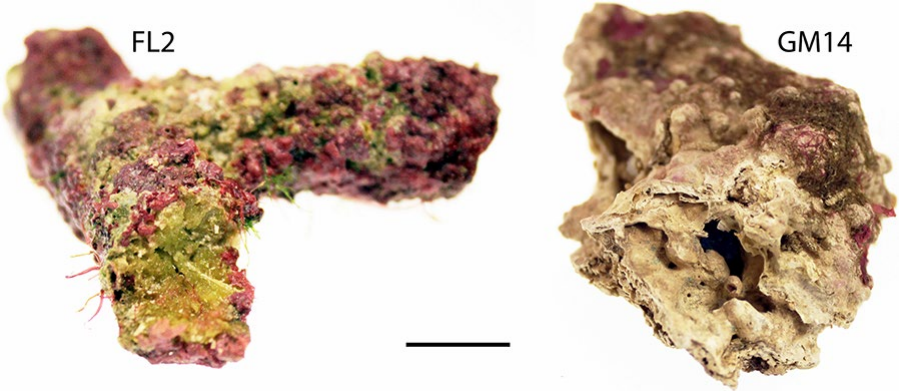
Drilled surfaces of microfloras samples FL02 and GM14. Note the dense (FL02) vs. light (GM14) phototroph colonization. *Scale bar* approximately 1.5 cm

Several microflora specimens were selected for environmental sequencing to build a clone library and for metabarcoding (Table 2, Fig. 3). These consisted of limestone fragments from reef rubble or substratum adjacent to coral colonies originating from the Ryukyu archipelago (JP01, JP03, JP04, JP06, JP07 and JP25), the Florida Keys (FL01 and FL02) and a northwestern Gulf of Mexico rhodolith (GM14). These specimens were lightly drilled within CaCO_3_ patches devoid of crustose epiliths with a sterile 1.6 mm (1/16’’) bit mounted on a Flex-Shaft Attachment (Model 225) powered by a Dremel 3000 rotary tool (Dremel^®^, Racine, WI, USA). For each microflora sample, DNA from a total of 40-80 mg of pulverized CaCO_3_ obtained from multiple drills was extracted with a PowerSoil DNA Isolation kit (MO BIO Laboratories, Carlsbad, CA, USA). Samples JP01 to JP07 were each extracted from limestone fragments pooled from multiple locations in the Ryukyus to maximize *tuf*A sequence diversity. Four of the above microflora samples were metabarcoded (JP07, FL01, FL02 and GM14); samples JP07 and FL02 were densely colonized with endolithic taxa whereas samples FL01 and GM14 were more lightly colonized (Fig. 3) and were thus metabarcoded at different sequencing depths (see “*tuf*A microflora assays” section). In preliminary cloning assays, two miscellaneous environmental samples comprising an aquarium window scrap exhibiting encrusting Ulvophyceae (E09), and endoliths underlaying a crustose coralline *Lithophyllum* sp. specimen (S15) were also processed (Table 2).

**Table 2:** Limestone samples processed via cloning and metabarcoding

Sample	Clon./Metab.^a^	Site	Depth (m)	Date	Substratum
FL01	−/+	Big pine key, Florida	4	09/2013	Reef rubble
FL02	−/+	Big pine key, Florida	4	09/2013	Reef rubble
JP01	+/−	Ryukyus^c^	<5	07/2012	Reef rubble
JP03	+/−	Ryukyus	<5	07/2012	Reef rubble
JP04	+/−	Ryukyus	<5	07/2012	Reef rubble
JP06	+/−	Ryukyus	<5	07/2012	Reef matrix
JP07	+/+	Ryukyus	<5	07/2012	Reef matrix/rubble
JP25	+/−	Ryukyus	<5	07/2012	Reef rubble
GM14	−/+	Ewing Bank, NWGM^b^	57	08/2008	Rhodolith
E09	+/−	–	–	03/2013	Aquarium window
S15	+/−	NWGM	65	08/2008	*Lithophyllum* sp.

^a^ Cloning and/or metabarcoding: performed (+), not performed (−)

^b^ Northwestern Gulf of Mexico

^c^ Mixed locations and sampling dates within the archipelago

### *tuf*A microflora assays

**Fig. 4:**
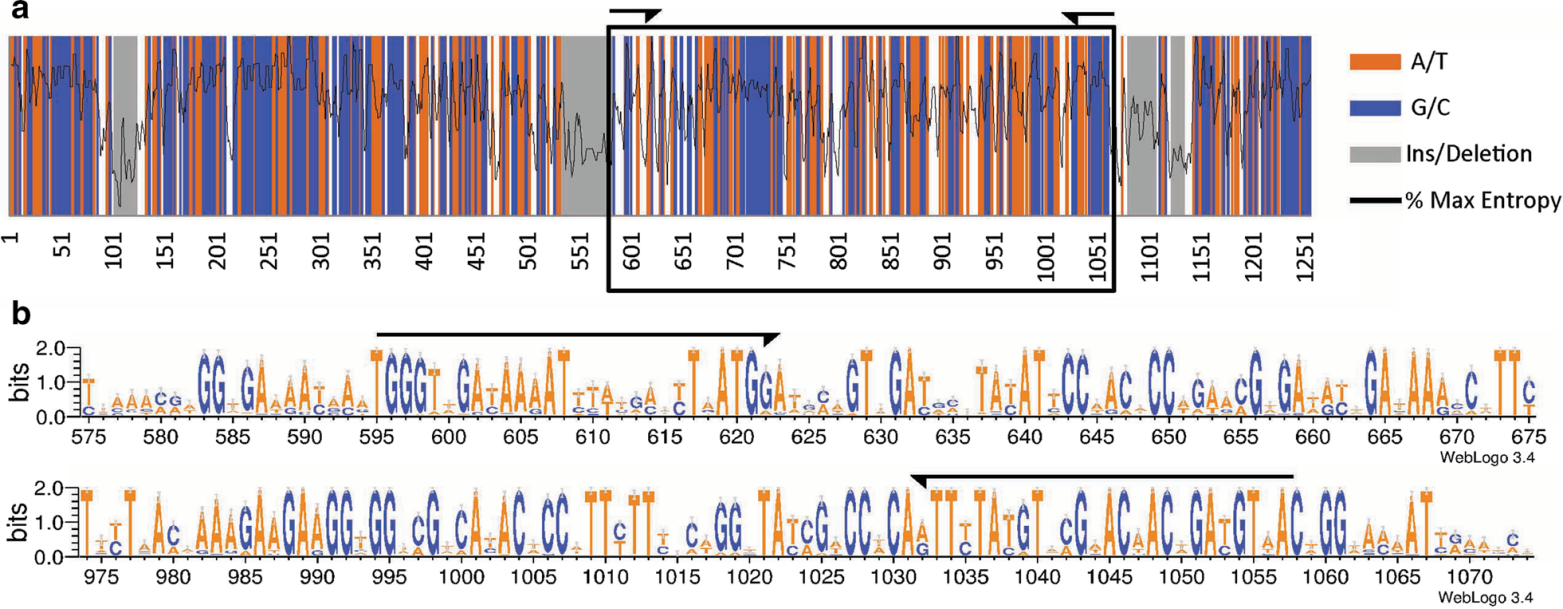
Metabarcoding *tuf*A. **a** Location of the metabarcode along 1251 bp of *tuf*A displaying sites with conserved A/T (*orange*) and G/C (*blue*) nucleotides, insertion-deletions regions (*grey*), and percentage of maximum entropy (as moving average). Note the lower proportion of conserved sites (i.e. greater informativeness) within the metabarcode. **b** Site conservation within the forward (*top*) and reverse (*bottom*) priming regions. All of the above were produced with (or from data output from) WebLogo 3.4

Degenerate primers (*env_tuf*AF/*env_tuf*AR, see Table 1) were designed with HYDEN [58] on a phylogenetically diverse alignment of phototroph sequences. These primers target a 462 base pair (bp) amplicon (407 bp without incorporated primers) that is nested in the 3′ half of *tuf*A (see Fig. 4, created with WebLogo 3.4, [15]). The produced amplicon does not overlap with the 5′ intron found in the Euglenophyta [67], and is devoid of codon insertion/deletions (some exist within the amplicon in heterotrophic bacteria). PCR products for cloning or Illumina-metabarcoding were amplified from DNA extracts on a low temperature/long annealing and long extension cycle to maximize diversity recovery (3 min at 95 °C, 1 min steps at 94, 42, and 72 °C for 40 cycles and a 5 min final 72 °C extension). Samples generally required 1:10th to 1:100th dilution for successful amplification due to DNA extract concentration variation and/or the potential presence of PCR inhibitors commonly found in algal samples (such as polysaccharides and natural products, [105]). For cloning, PCR products were separated with a TOPO^®^ TA Cloning^®^ Kit (One Shot^®^ Top 10 chemically competent *E. coli*, Invitrogen™, Life Technologies, Grand Island, NY) following the manufacturer’s protocol. Clones were grown on LB agar plates containing 50 µg/mL Ampicillin and 40 µg/mL X-Gal. PCR was performed on white colonies with primers *env_tuf*AF and M13R (M13 Reverse priming site on TOPO^®^ vector) and conditions as above. Colony PCR products of correct size were sequenced commercially and assembled in Sequencher v5.1. For metabarcoding, DNA extracts were shipped to MRDNA (http://www.mrdnalab.com, Shallowater, TX, USA), where PCR products were amplified using a HotStarTaq Plus Master Mix Kit (Qiagen, Valencia, CA, USA) with indexed *env_tuf*AF primers (Additional file 3: Table S3). The amplification of the densely colonized samples JP07 and FL02 at MRDNA produced strong PCR products, while the more lightly colonized samples GM14 and FL01 resulted in weaker PCR products (in congruence with PCR testing conducted at LAF prior to the shipping of these samples). Thus, JP07 and FL02 products were pooled in higher proportion for deep sequencing while products from FL01 and GM14 were normalized and pooled with microbiota assays from other customers (i.e. 16S rDNA) to produce a nominal 20,000 reads per assay (as routinely performed at MRDNA). The prepared library (TruSeq DNA Sample Prep Kit for 2 × 250 bp paired-ends) was sequenced on the lane of an Illumina MiSeq Platform (Illumina Inc, San Diego, CA, USA).

### De novo clustering of *tuf*A OTUs

Raw Miseq reads were processed with the USEARCH pipeline (http://www.drive5.com/uparse/) [26] to overlap paired-ends (-*fastq_mergepairs*, Q = 2), demultiplexing (including stripping of indexes and forward primers, *fastq_strip_barcode_relabel.py* script), filter for high quality reads (-*fastq_filter*) of appropriate size (expected error E >0.5, min 400 bp, max 440 bp), global trimming (5′ cropping of 20 bp, 3′ cropping of 50 bp), and removal of noisy reads after dereplication (singletons, doubletons and tripletons found across the entire data set), as well as chimeras and contaminants (non-*tuf*A reads) with UCHIME (command -*uchime_ref*) and UBLAST (-*ublast*) (Table 3). The dereplication file (with remaining reads, average read length of 375 bp) was then clustered with UPARSE (-*cluster_otus*) at the recommended 97 % global threshold and with SWARM [62] at multiple local thresholds (d = 1 to 16) to explore its clustering optima (none published). UPARSE (97 %) and SWARM (d = 10) outputs were then parsed to identify robust core OTUs generated by both algorithms to follow recent recommendations for reproducibility with the use of multiple algorithms [88]. Core OTUs were then mapped at the 97 % level (with -*usearch_global*) against the quality-filtered (merged) reads (including singletons, doubletons and tripletons) to produce an OTU abundance table (*uc2otutab.py* script). Cumulative and non-cumulative rank-abundance curves were built from this table to explore the microflora’s assemblage structure.

**Table 3:** Remaining reads throughout the UPARSE pipeline as counts and percentage of raw reads

Raw	4,917,888	100 %
Paired-ends merging	4,720,138	96
Demultiplexing	1,928,898	39
Read quality filter	1,465,438	30
Read size filter^a^	1,331,438	27
Denoising^b^	837,069	17
Decontamination^c^	824,355	17

^a^ Reads used for OTU mapping

^b^ Singletons, doubletons and tripletons

^c^ Non-*tuf*A reads and chimeric reads

### *tuf*A reference database

GenBank^®^’s *tuf*A sequences were summarized into a non-redundant local database augmented with newly generated Sanger data (including clones). We retrieved with BLASTn the 20,000 closest matches to multiple *tuf*A sequences representing major branches of photosynthetic algal diversity (eukaryotic and prokaryotic). Search results were pooled, filtered for sequences >300 bp, and dereplicated (100 % identity). Following preliminary tree building, sequences of prokaryotic heterotrophs (eubacteria other than cyanobacteria) were segregated and used for separate searches as above. The numerous resulting heterotroph sequences were dereplicated at 99 %. We excluded the non-photosynthetic apicoplast-encoded *tuf*A of the Apicomplexa [11] and the nuclear-encoded *tuf*A of the Streptophyta [3] because of conflicting phylogenetic signal (see [19, 43, 49]) and irrelevance to the target group/habitat investigated herein (i.e. lower phototrophs inhabiting CaCO_3_). Nonetheless, the deepest branch of the Streptophyta, i.e. the Charophyte algae *Mesostigma* and *Chlorokybus* [56, 101] were maintained in the database because their *tuf*A is chloroplast-encoded. The only two sequences available for the terrestrial order Trentepohliales (*Cephaleuros* and *Trentepohlia*, Ulvophyceae) were also excluded for their large divergence with other eukaryotic *tuf*A and ambiguous phylogenetic placement (until genome sequencing clarifies the organellar localization of their *tuf*A, i.e. nuclear or chloroplastic). The final sorted *tuf*A database comprised 4057 sequences (1339 phototrophs and 2718 heterotrophs). We followed PHYTO-REF for the classification of Diatoms (namely a single phylum-class noted as ‘Bacillariophyta’ within the Ochrophyta) and otherwise followed the Algaebase class-scheme [37]. Within the Ulvophyceae, we adopted the combined notation ‘Ulvales-Ulothrichales’ [55]. A summary of the database sequence content is provided in Additional file 4: Table S4.

### *tuf*A phylogenies

Phylogenetic reconstruction of the *tuf*A database was performed to visualize its diversity and for clade-based (topological) identification of clones and OTU sequences (n = 802). In order to produce a diverse heterotroph outgroup while greatly reducing sequence number for efficient computation time, we ‘framed’ bacterial reference diversity by selecting the most distant haplotypes in large clades observed in preliminary trees (n = 556 heterotroph sequences kept). The final dataset comprising 2697 sequences was translated into amino acids and aligned with MUSCLE [24]. Regions with poor homology (codon insertion/deletions) were cropped between sites of conserved amino acids (see Fig. 4) and the sequences translated back to nucleic acids. The final alignment (891 bp) was then ran with RAxML-HPC2 on the CIPRES computer cluster (http://www.phylo.org) with a GTR+I+G model of evolution partitioned per codon position, 200 topological searches from random restarts and 1000 bootstrap replicates for node support estimation. To further detail diversity in euendolithic Ulvophyceae, and for species delimitation analyses (see below), the above phototroph alignment was subsetted for the orders Bryopsidales and ‘Ulvales-Ulothrichales’ and few outgroup taxa. Previously dereplicated barcode data were reintroduced to show sampling effort in these orders. The final alignment comprising 906 sequences was used for phylogenetic analysis as listed above (with 1000 restarts and 1000 bootstrap replicates) and species delimitation analyses (see below). All trees were edited in iTOL [57].

### Ulvophycean species delimitation

Branch lengths were extracted from the RAxML Ulvophyceae tree with function *cophenetic.phylo* of the package APE in R [69, 78] to produce a distance matrix as input for the standalone version of the Automatic Barcode Gap Discovery software (ABGD, [76]). The latter was run with minimum (pmin) and maximum (pmax) intraspecific distance priors comprised between 0.001 and 1 in 100 steps, and with a 0.5 relative gap width. Alternative species boundaries hypotheses were produced with the general mixed yule coalescence (GMYC) model with the package SPLITS in R [35], with the single threshold method based on an ultrametric tree generated in BEAST v2.0 [8] using a relaxed log-normal clock with a constant population coalescent as prior, and a GTR+I+G model of evolution partitioned per codon position. Markov Chain Monte Carlo (MCMC) chains were run for 30 million generations (sampled every 1000th generations) and the quality of the run assessed in Tracer v1.6 [80] to ensure that effective sample size (ESS) values were >200 with the default burnin (3000 trees).

### Divergence and identity thresholds

The *tuf*A database alignment was cropped to the metabarcode length (i.e. clustered OTUs of 375 bp) and a pairwise percent identity matrix created with function *dist.dna* in package APE (R) (a few sequences with >20 bp of missing data on their 5′ or 3′ side were excluded in order to avoid inflation of computed percent identity values). From this distance matrix, boxplots depicting the amount of divergence within individual families, suborders and orders of the Ulvophyceae (‘Ulvales-Ulothrichales’ and Bryopsidales) were drawn in order to examine their validity. Next, to define clade-based (conservative) identity thresholds for the rapid annotation of OTU at multiple taxonomic ranks, the function *sppDist* of the package SPIDER in R [10] was used to compute the overall intra- and inter- clade divergence values for all phototrophs at high-levels ranks (domain, phylum, and class clades) and at lower levels for the Ulvophyceae (order, suborder, and family clades, as well as molecular species clades delimited by ABGD and GMYC). *In silico* (relaxed) thresholds for the above ranks were also assessed by scoring taxonomic discrepancies between an OTU’s best hit (obtained with function -*usearch_global*) and its topological reference taxonomy. From this procedure, the distribution of correctly and incorrectly classified hits was reported (for all phototrophs and the Ulvophyceae). Finally, to assess the overall performance of the database in light of annotation thresholds defined above (i.e. “How distant are OTUs from databased sequences?” and “Do abundant OTUs score highly against the database?”), the distributions of OTUs’ best hit (percent identity) against the database was plotted for all phototrophic and Ulvophycean OTUs, as well as for the most abundant OTUs (i.e. those representing ≥1 % of reads) in each of the metabarcoded samples.

### Availability of supporting data

The annotated database and annotated core OTUs are available in the Dryad repository, doi:10.5061/dryad.6cj8h. Newly barcoded specimens and clones are deposited in Genbank^®^ (KU361834-KU362236) and the raw Illumina dataset on the National center for Biotechnology and Information (NCBI) Sequence Read Archive (#SRP067712).

## Results

### DNA Sequencing

**Fig. 5:**
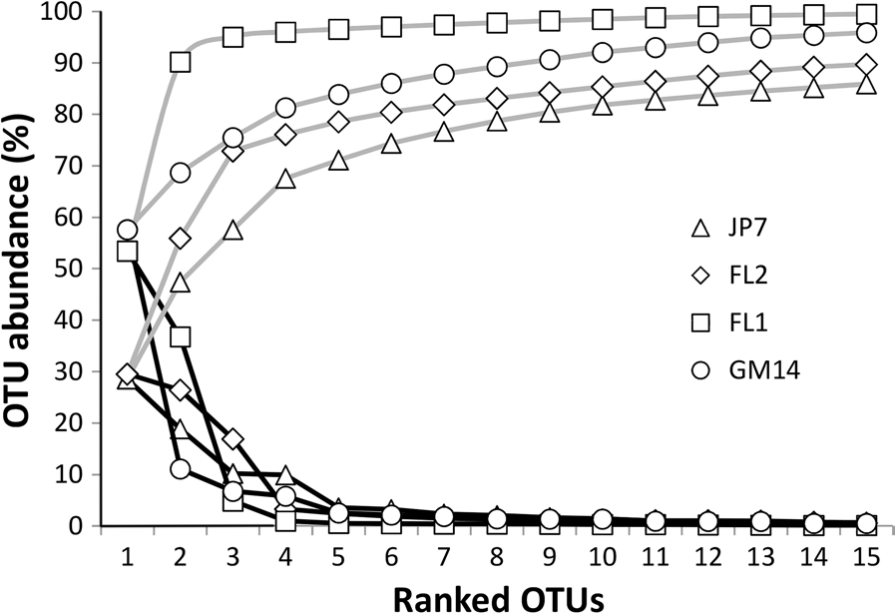
Cumulative and non-cumulative OTU-ranked abundance curves. *Curves* are displayed for the first 15 ranking OTUs only. See Additional file 6: Figure S1 for the extended cumulative abundance curve

A total of 316 specimens were barcoded with *tuf*A (including 238 Ulvophyceae, 72 Florideophyceae, and six Phaeophyceae) among which 104 were new representatives of ‘*Ostreobium*’ spp. (47 from cultures) (Additional file 1: Table S1, Additional file 2: Table S2). Cloning resulted in the sequencing of 153 bacterial colonies, which after dereplication represented a library of 86 unique *tuf*A sequences, including 37 Chlorophyta, 25 Cyanophyta, 15 Rhodophyta, one Haptophyta and eight Ochrophyta (see Additional file 5: Table S5 for class details). The shared Miseq run output was a total of 4,917,888 raw paired-end reads for the four microfloras tested. Upon data processing throughout the USEARCH pipeline, read numbers gradually decreased, leaving 27 % of the raw data for OTU abundance mapping and ~17 % for clustering (Table 4). Clustering at multiple levels with SWARM (d = 1–16) output from 2483 to 740 OTUs. SWARM indicated a clear clustering optima between local thresholds of d = 10–13 (i.e. up to 13 steps from a given centroid), where the number of common OTUs with UPARSE did not vary (Table 5). Clustering at the 97 % recommended global threshold with UPARSE generated 822 OTUs, the majority but 20 were also produced by SWARM. The 802 core OTUs common to SWARM and UPARSE mapped to 1,260,811 (~94 %) of the 1,331,438 reads available (70,243 reads unmapped) (Table 4). The number of OTUs and their final read counts for each of the microflora samples clearly reflected the differential sequencing depth carried at MRDNA. Non-cumulative ranked abundance curves demonstrated 3–5 OTUs strongly dominating each of the microfloras and the presence of numerous low abundance OTUs (i.e. long tails, Fig. 5). Cumulative curves (ranked OTUs) demonstrated rapid plateauing of mapped read abundance (Fig. 5 and Additional file 6: Figure S1). Phototrophs represented 618 out of the 802 robust OTUs, i.e. 77 % of OTUs (53.3–84.6 % depending on the sample) and overall 98 % of mapped reads (83.2–99.4 % depending on the microflora sample) (Fig. 13, Additional file 7: Table S6).

**Table 4:** OTU counts and corresponding read counts per microflora sample

	OTUs	Reads
Mapped	802	1,261,195
Corrected^a^	802	1,260,811
FL01	28	23,785
FL02	317	558,503
JP07	609	672,119
GM14	38	6404

^a^ Table cells with read counts <3 were removed

**Table 5:** Number of OTUs common to UPARSE and SWARM output at multiple clustering thresholds

	UPARSE	99 %	98 %	97 %	96 %	95 %
SWARM		1070	918	***822***	761	698
d01	2483	1044	913	***818***	752	696
d04	1416	963	902	***813***	750	693
d08	1208	851	845	***806***	748	690
d09	1135	835	829	***804***	747	689
***d10***	***1093***	***833***	***827***	*802*	***747***	*689*
***d11***	***1066***	***833***	***827***	*802*	***747***	*689*
***d12***	***1047***	***833***	***827***	*802*	***747***	*689*
***d13***	***1032***	***833***	***827***	*802*	***747***	*689*
d14	788	735	735	***735***	719	676
d15	763	722	722	***723***	708	673
d16	740	706	706	***707***	696	668

Recommended global threshold for UPARSE (97 %) and determined local threshold optima for SWARM (d10–d13) are in bold italics. The 802 OTUs kept for subsequent analyses are italicized

### Phototroph phylogeny

**Fig. 6:**
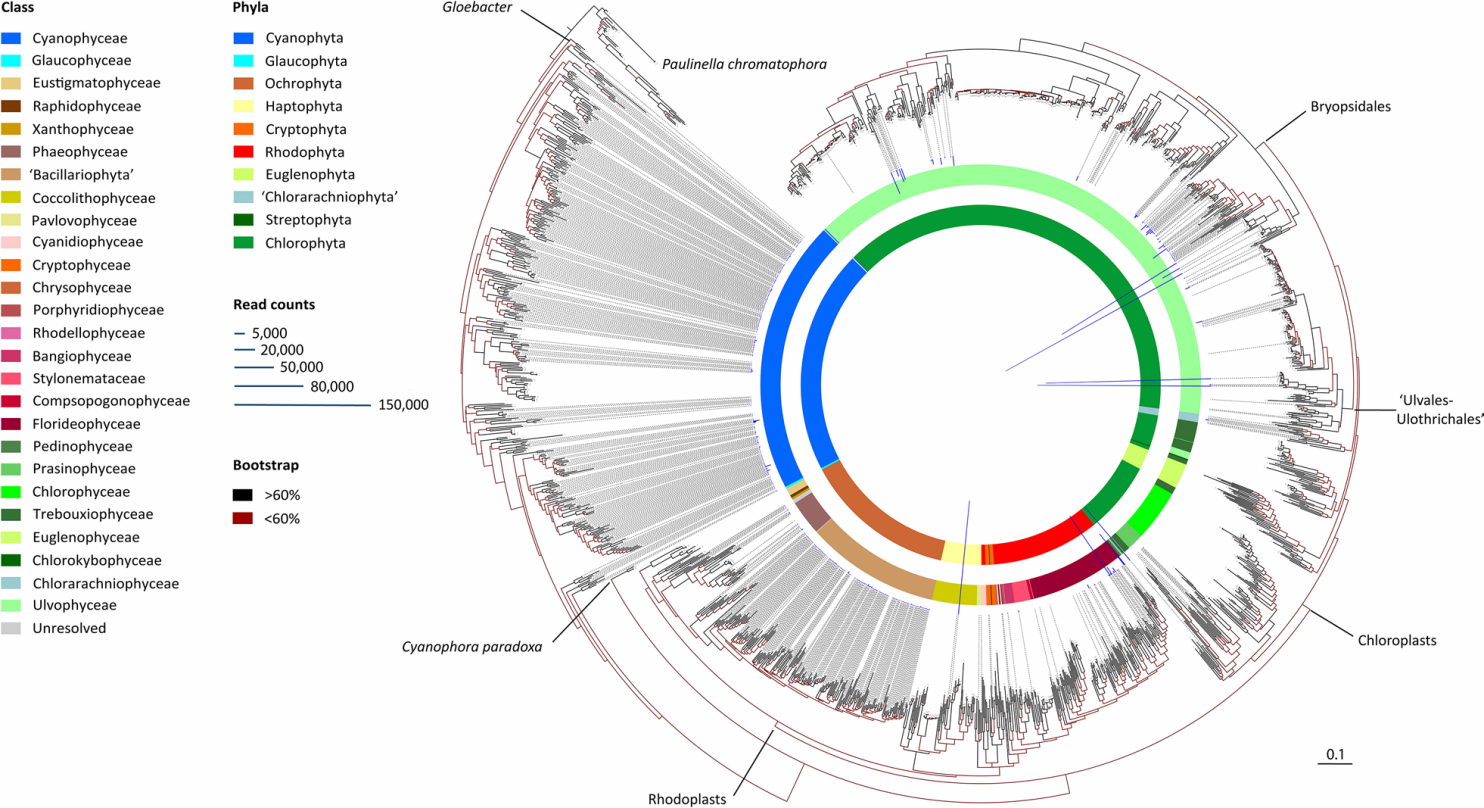
Phototrophic diversity. RAxML tree displaying 2141 *tuf*A sequences of ‘Phototrophs’ (including OTUs) rooted with 556 sequences of ‘Heterotrophs’ (not shown) and based on an alignment of 891 bp. The tree was edited in iTOL for bootstrap support, OTU abundance (*vertical bars* topping *dashed lines*) and taxonomy (*inner ring* Phyla, *outer ring* Classes). A few phylogenetic landmarks are reported on the tree margin as well as stems leading to orders of the Ulvophyceae (Bryopsidales and ‘Ulvales-Ulothichales’)

Phylogenetic reconstruction of phototrophic *tuf*A diversity recovered topological features congruent with those presented in Delwiche et al. [19], albeit with much denser taxon sampling. The resulting tree was characterized by a poorly resolved backbone with higher resolution at lower taxonomic levels. Phylum para/polyphyly was caused by the nesting of secondary endosymbiotic phyla (Ochrophyta, Haptophyta, Cryptophyta; and Euglenophyta and ‘Chlorarachniophyta’) among extent members of early (single-celled) lineages of the Rhodophyta and Chlorophyta [46] from which they diversified (respectively). A few classes are also para/polyphyletic in congruence with known taxonomic discrepancies (e.g. see [34]). Subclasses of the Cyanophyta (not shown) are also mostly polyphyletic (e.g. Synechococcophycideae) and distributed between two major clades (as in e.g. [63, 83]). Overall, the phototrophic diversity comprised in the database includes 27 classes in 10 phyla (Fig. 6, Additional file 4: Table S4). Several phylogenetic landmarks in the evolution of photosynthetic organisms are highlighted on the tree (Fig. 6).

### OTU richness and abundance

The phylogenetic distribution of OTUs (richness and abundance) is clearly visible on the phototroph tree (see dashed lines and bars’ height, Fig. 6) and allowed their classification (on a topological basis) to 14 classes in seven phyla. All but 22 eukaryotic OTUs could not be assigned with confidence to a specific class within the phylum they branched in (Additional file 8: Table S7); however, these OTUs represented very few reads (<0.25 % of all mapped reads). The densely colonized microfloras FL02 and JP07 (i.e. those sequenced deeply) exhibited particularly similar richness profiles for phylum, class, and Ulvophycean family (Fig. 13). Together, these two microfloras harbored 605 phototrophic OTUs, 141 of which were assigned to both samples following demultiplexing (Additional file 9: Figure S2, Additional file 8: Table S7). Their abundance profiles were also similar except at the family level (Fig. 13). By comparison, the less dense microfloras FL01 and GM14 (i.e. those with low sequencing depth) showed skewed profiles toward particular groups and much fewer OTUs (33 phototrophic OTUs, 13 unique to these two samples, 20 shared with others). In the coral reef samples (i.e. FL01, FL02, and JP07), the Cyanophyceae (Cyanophyta) and ‘Bacillariophyta’ (Ochrophyta) were found in low read abundance but revealed tremendous OTU diversity (267 and 162 OTUs, respectively), while the Coccolithophyceae (Haptophyta), the Pedinophyceae and Ulvophyceae (Chlorophyta) and the Florideophyceae (Rhodophyta) were found in large read abundance but were much less diverse (i.e. few OTUs, Fig. 6). In the rhodolith sample (GM14), the Florideophyceae were particularly abundant.

### Ulvophyceae phylogeny

**Fig. 7:**
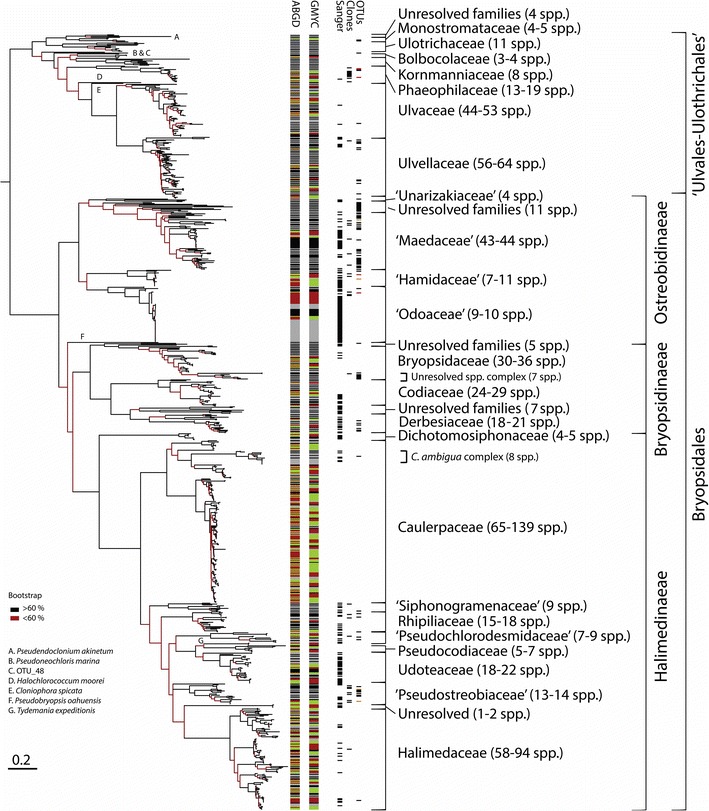
Ulvophyceae diversity. RAxML tree displaying 901 *tuf*A sequences of the order Bryopsidales and ‘Ulvales-Ulothrichales’ based on an alignment of 891 bp. The tree was rooted with few outgroup taxa of the Chlorophyta and edited in iTOL for bootstrap support, molecular species, and sequencing method (Sanger, Cloning or Metabarcoding). OTU abundance color-coded for high read counts (*red*), moderate (*brown*) and low (*black*). Congruent clusters of molecular species between ABGD and GMYC are represented in alternating *dark* and *light grey* colors. Incongruent species boundaries are displayed in alternating colors *yellow* and *red*. Taxonomy is reported on the tree margin. Note important incongruence in molecular species delimitation between the two methods in the families Caulerpaceae and Halimedaceae

**Fig. 8:**
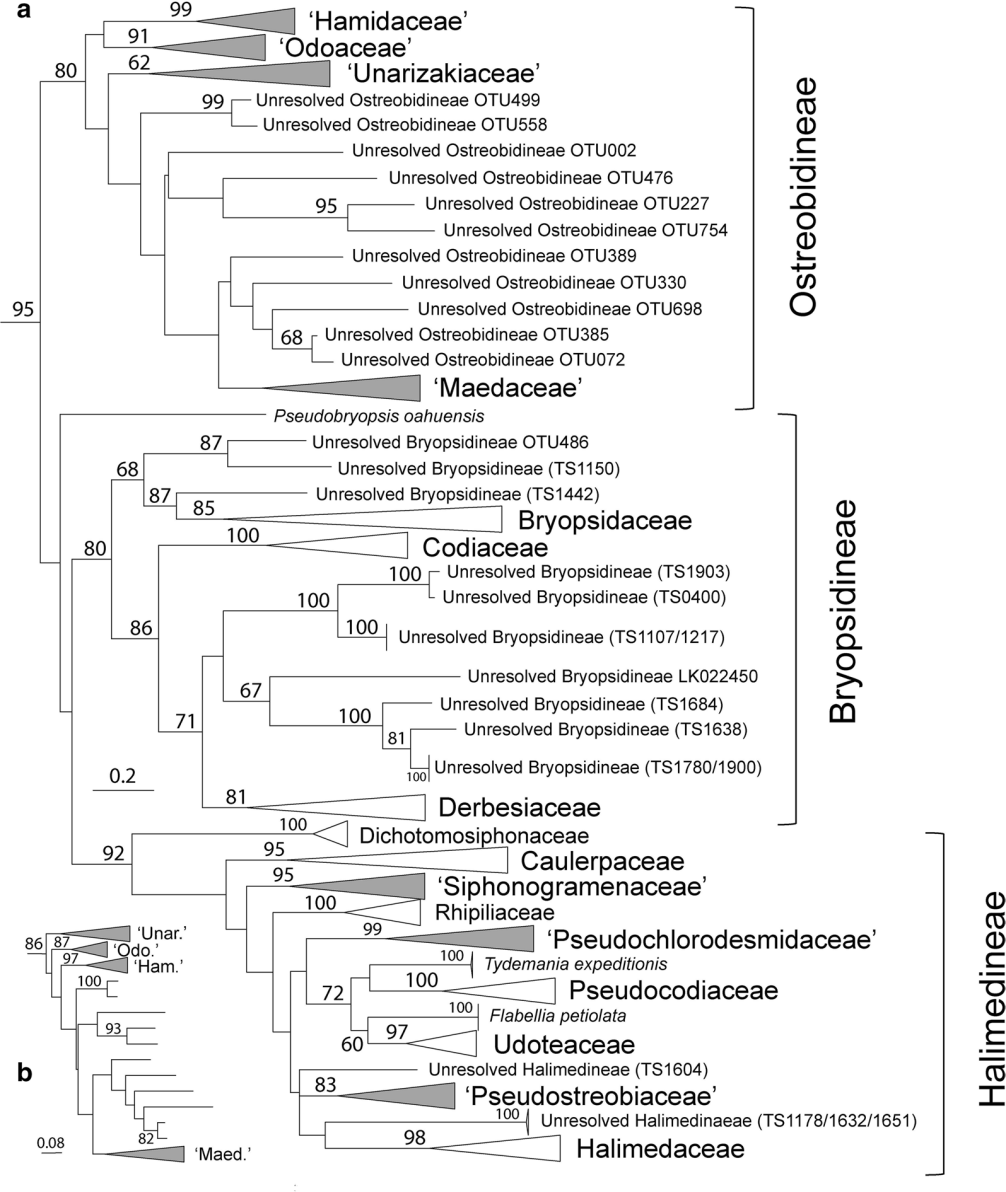
Family diversity in the order Bryopsidales. **a** Summary family topology of the Bryopsidales obtained with RAxML for the Ulvophyceae data set (see Fig. 7). **b** Close-up of alternative family branching order in the Ostreobidineae obtained with RAxML for the full database tree (magnified from Fig. 6). Families provisionally delimited in the present study are shaded in *gray*

**Fig. 9:**
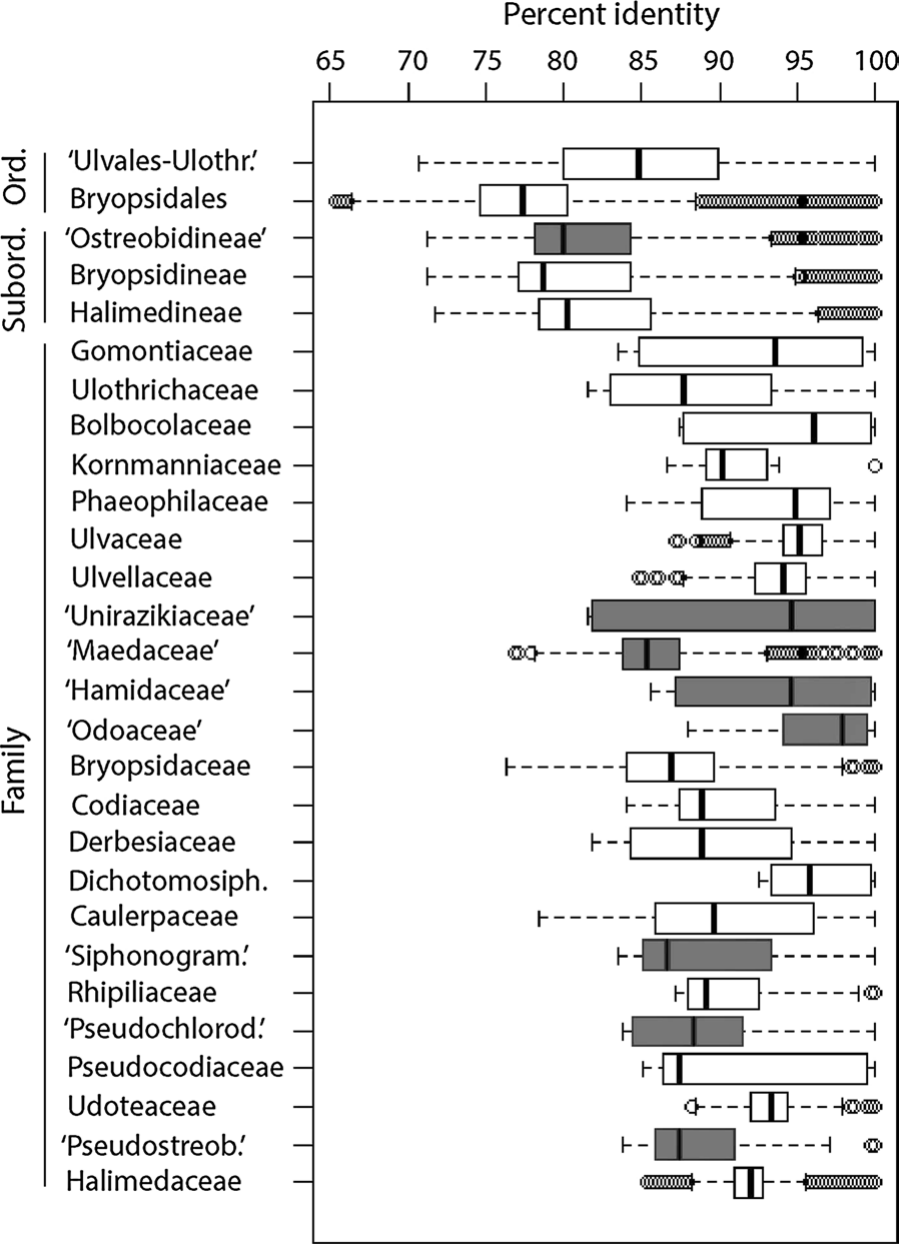
Molecular divergence in the Ulvophyceae. Percent identity values computed based on an alignment of 375 bp of the *tuf*A metabarcode. Provisional suborder and families delimited in the present study are shaded in *gray*. Abbreviations as follows: ‘*Ord.*’ Order, ‘*Subord.*’ Suborder, ‘*Ulvales-Ulothr.*’ Ulvales-Ulothrichales, ‘*Dichotomosiphon.*’ Dichotomosiphonaceae, ‘*Siphonogram.*’ Siphonogramenaceae, ‘*Pseudochlorod.*’ Pseudochlorodesmidaceae, ‘*Pseudostreob.*’ Pseudostreobiaceae

The phylogenetic estimation made on the Ulvophyceae dataset (Fig. 7) resulted in nearly identical branching features than within the larger tree except for a few differences in the Bryopsidales (shown for the Ostreobidineae, see Fig. 8). The Bryopsidales and the ‘Ulvales-Ulothrichales’ radiations each received strong bootstrap support (95 and 82 %, respectively). Overall, the tree details 23 families, some accepted taxonomically, others provisionally delimited here based on topological features and clade divergence. ‘*Ostreobium*’ is revealed as a polyphyletic form genus representing a complex of family-level clades (Figs. 7, 8, 9). Most of its diversity is monophyletic within a large, confirmed subordinal-level clade, the Ostreobidineae (previously proposed by [102]), whose divergence is particularly consistent with other suborders, the Bryopsidineae and the Halimedineae (Fig. 9). In this suborder, four ‘*Ostreobium*’ families are provisionally delimited and named from their location of collection in the Ryukyus for the purpose of molecular classification (*nomina nuda* ‘Hamidaceae’, ‘Maedaceae’, ‘Odoaceae’ and ‘Unarizakiaceae’, Fig. 9). Few OTUs are found in the phylogenetic vicinity of the ‘Maedaceae’ and presently cannot be assigned to a family clade (Figs. 7, 8). Additional ‘*Ostreobium*’ diversity is found in a remote family-level clade of the suborder Halimedineae provisionally assigned for taxonomic simplicity to the ‘Pseudostreobiaceae’ (Figs. 7, 8, 9). Our barcoding efforts also further documented members of the diminutive epilithic, polyphyletic form genus ‘*Pseudochlorodesmis*’ delimited here in two provisionally family-level clades named based on their taxonomic history [103], i.e. the ‘Pseudochlorodesmidaceae’ and Siphonogramenaceae’ (Figs. 7, 8, 9). Other ‘*Pseudochlorodesmis*’ spp. (see Additional file 1: Table S1 for details) represented primordia of otherwise conspicuous taxa in families of the Halimedineae (e.g. in *Halimeda*, see [64]) and neotenic thalli within the Rhipiliaceae [103] and the Caulerpaceae (a sister taxon to the *C.* ‘*ambigua*’ complex, [23]). Barcoding of diminutive, simple Bryopsidineae also revealed numerous unresolved/unknown taxa in the phylogenetic vicinity of the Derbesiaceae and Bryopsidaceae (Figs. 7, 8). Several epilithic families are found endolithically as seen by the presence of OTUs in their clade (Fig. 7, e.g. the Bryopsidaceae, Derbesiaceae and Halimedaceae) supposedly as reproductive cells, germlings and/or endolithic siphonous networks).

### Delimited species

The GMYC analysis delimited 504 hypothetical species in the Ulvophyceae (LGMYC = 7663.072> L0 = 7577.771.182, P = 0) while with ABGD the output was 666 spp. Species groups delimited by the two methods (GMYC-ABGD) represented 349–485 spp. in the Bryopsidales, and 147–172 spp. in the ‘Ulvales-Ulothrichales’ (Fig. 7). The majority of discrepancies arose in the Bryopsidales in well-sampled families of the suborder Halimedineae (overall 195–308 spp.), namely in the Halimedaceae (56–88 spp.) and especially in the rapidly diversifying Caulerpaceae (67–132 spp.), where GMYC seemed to overlump and ABGD oversplit terminal clades. By contrast, species delimitation showed greater congruence between the two methods in the remainder of the Ulvophyceae, in the ‘Ulvales-Ulothrichales’ (Ulvellaceae: 56–65 spp., Ulvaceae: 43–52 spp., Phaeophilaceae: 13–18 spp.) and in the Bryopsidales within the suborders Bryopsidineae (overall 85–91 spp.) and Ostreobidineae (overall 72–81 spp.; 53–58 spp. in the Ryukyus alone). The remote *Ostreobium* clade found in the Halimedineae, i.e. the ‘Pseudostreobiaceae’, harbors 13–14 spp. Molecular species divergence computed over the metabarcode length (375 bp) indicated species boundaries lying between 99.73 and 97.87 % with the GMYC method (i.e. 1–8 bp substitutions), and between 99.73 and 99.47 % with ABGD (1–2 bp substitutions) (not shown, see Additional file 10: Figure S3).

### Identity Threshold for annotation

**Fig. 10:**
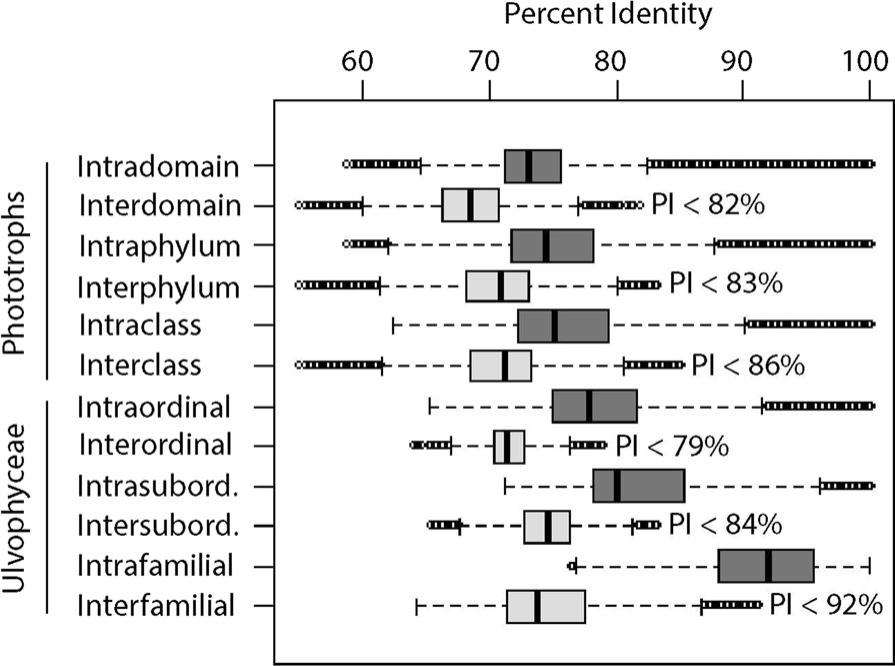
Conservative annotation thresholds. Overall intra- and inter-molecular divergence (*dark* and *light grey*, respectively) at the domain, phylum and class levels for all phototrophs and at the ordinal, subordinal and family levels within the Ulvophyceae (orders Bryopsidales and ‘Ulvales-Ulothrichales’). Percent identity values were computed based on an alignment of 375 bp of the *tuf*A metabarcode. Minimum conservative thresholds for metabarcode classification/annotation are printed. *PI* percent identity

**Fig. 11:**
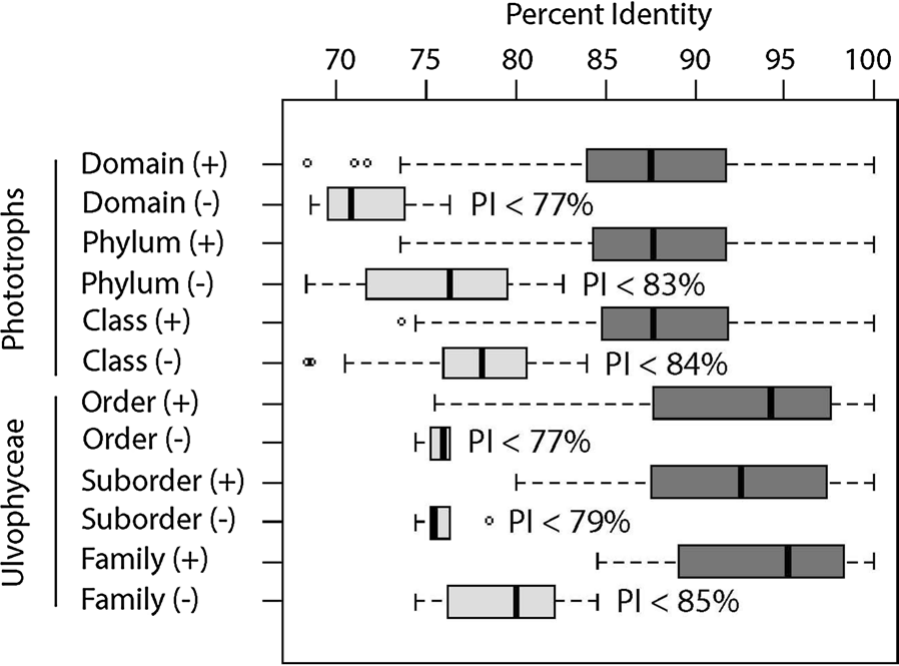
Relaxed annotation thresholds. Distribution of taxonomic match (+)/mismatch (−) between metabarcodes’ best hit and their tree-based classification for all phototrophs and the Ulvophyceae. Percent identity values were computed based on 375 bp of the *tuf*A metabarcode. Minimum thresholds to avoid misclassification are printed. *PI* percent identity

**Fig. 12:**
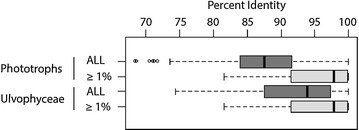
Database performance. Distribution of OTU’s best hit against the database for phototrophs and for the Ulvophyceae-only (orders Bryopsidales and ‘Ulvales-Ulothrichales’). ‘ALL’ represents matches for all OTUs regardless of abundance (in *dark grey*). ‘≥1 %’ represents matches for the most abundant OTUs (in *light grey*). Note the skewed distribution toward high identity matches for the most abundant OTUs (regardless of taxonomy) indicating high performance of the database for annotation of the targeted communities. Values were computed based on 375 bp of the *tuf*A metabarcode

**Fig. 13:**
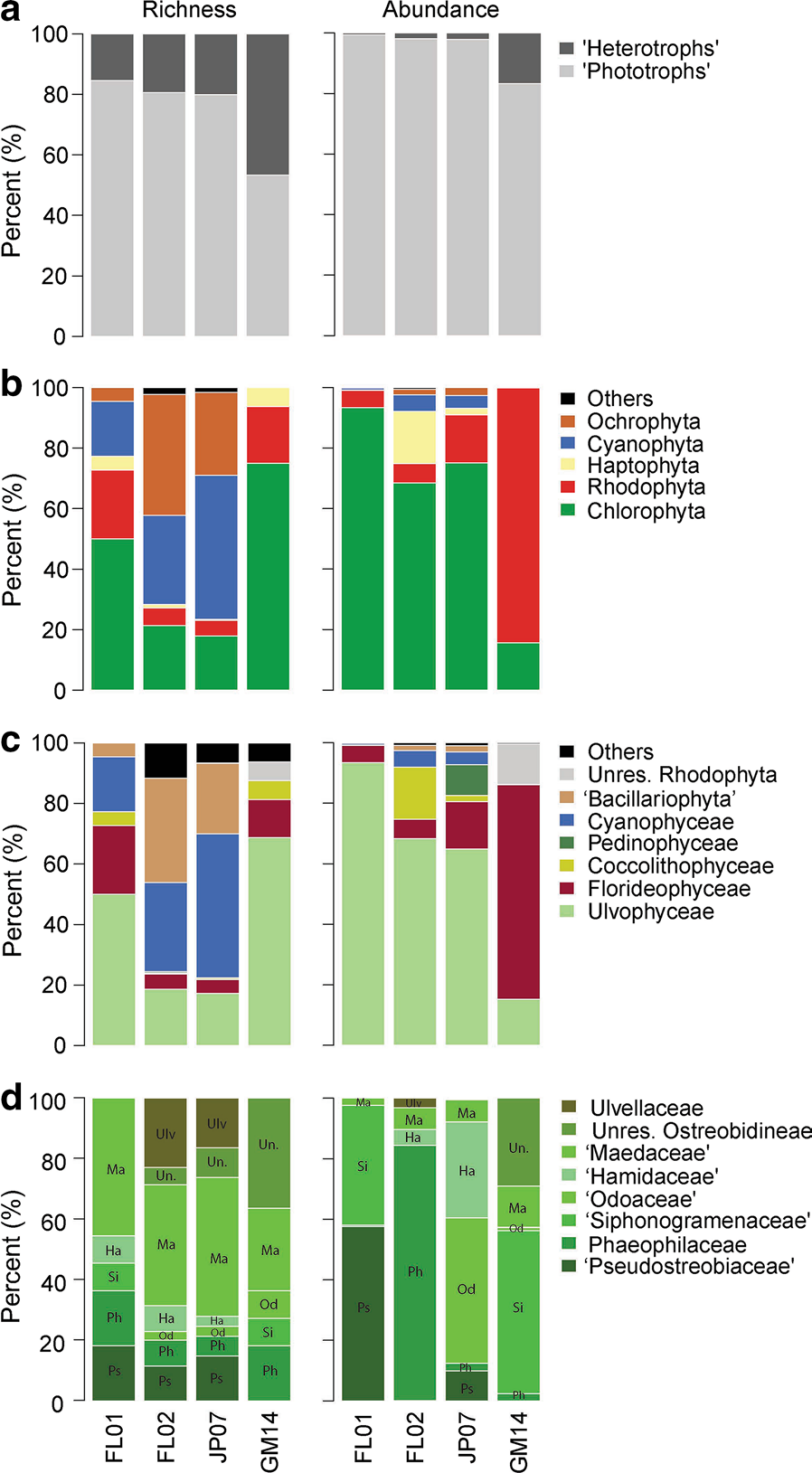
OTU richness and abundance at multiple taxonomic levels. **a** ‘Phototrophs’ vs. ‘Heterotrophs’, **b** photrophic phyla, **c** phototrophic classes, and **d** Ulvophycean families within orders Bryopsidales and ‘Ulvales-Ulothichales’. Richness represents OTU diversity as percentage of the total number of OTUs. Abundance represents OTUs read counts as percentage of total mapped reads per sample. Family abbreviations as follows: ‘*Ulv*’ Ulvellaceae, ‘*Un.*’ Unresolved Ostreobidineae, ‘*Ma*’ Maedaceae, ‘*Ha*’ Hamidaceae, ‘*Od*’ Odoaceae, ‘*Si*’ Siphonogramenaceae, ‘*Ph*’ Phaeophilaceae, ‘*Ps*’ Pseudostreobiaceae. ‘*Others*’ includes very low abundance representatives (each <1 % of mapped reads in average) of phylum Cryptophyta and ‘Chlorarachniophyta’ and the classes Bangiophyceae, Compsopogonophyceae, Chlorarachniophyceae, Cryptophyceae, Pavlovophyceae, Phaeophyceae, Prasinophyceae, Stylonematophyceae and unresolved members of the Chlorophyta, Haptophyta, and Ochrophyta (See Additional file 7: Table S6, Additional file 8: Table S7; Additional file 11: Figure S4 for further details)

Examination of taxonomic rank divergence obtained with *sppDist* within the Ulvophyceae (Fig. 10) indicated conservative annotation thresholds for family, suborder and order boundaries at 92, 84 and 79 %, respectively (values rounded up from 91.2, 83.2 and 78.9 %, respectively) and relaxed annotation thresholds at 85, 79, 77 %, respectively (values rounded up from 84.5, 78.4, and 76.3 %) (Fig. 11). For the full breadth of phototrophic OTUs, conservative annotation thresholds for class, phylum and domain boundaries laid at 86, 83 and 82 %, respectively (values rounded up from 85.1, 82.2 and 81.9 %), while relaxed annotation thresholds laid at 84, 83 and 77 %, respectively (values rounded up from 84.0, 82.7 and 76.3 %) (Figs. 10, 11, respectively). Overall, phototrophic OTUs matched the database at high identity levels with the majority of hits ≥84 % (see lower boxplot quartiles, Fig. 12) in the range of class annotation (i.e. relaxed = 84 %, conservative = 86 %). For the Ulvophyceae, the majority of hits were ≥87 % (see lower boxplot quartiles, Fig. 12) in the range of family-level thresholds (i.e. relaxed = 85 %, conservative = 92 %). The most abundant OTUs, those driving abundance profiles (i.e. >1 % abundance, Fig. 13 and Additional file 11: Figure S4), hit the database at even higher identity values (lower quartile >91 %, Fig. 12) and displayed the same distribution for the phototrophs (n = 32 OTUs) or the Ulvophyceae-only (n = 19 OTUs).

## Discussion

### Bioinformatics

Following demultiplexing, we noted important read losses (>50 %) (Table 3) probably caused by the systematic exclusion of reads containing base pair error(s) within their index in the early steps of the UPARSE pipeline (no mismatch allowed). Here, demultiplexing losses may have also been exarcerbated by the length of our amplicon (467 bp) for the chemistry used (2 × 250 bp) since paired-ends could not overlap over the index regions. An additional consequence of potential increased substitution errors in the index region along with tag jumping in Illumina-based metabarcoding [89], is the misassignment of sequence-to-sample (i.e. the false assignment of an OTU to a given sample), which may inflate OTU diversity across samples and thus bias richness profiles. However, since index error/jumping generally concerns only a small proportion of reads, false assignment is less likely to bias abundance profiles (Fig. 13), especially when those profiles are underlayed by very abundant OTUs such as shown here (Fig. 5 and Additional file 11: Figure S4). Although we noted some possible tag error/jumping in OTUs retrieved in the deeply sequenced microfloras FL02 and JP07 (Additional file 12: Figure S5), numerous unique OTUs were also found in each of these samples (Additional file 9: Figure S2), (Fig. 13). If there is any bias, we suspect that the richness profile reported for the Ulvophyceae (Fig. 13, bottom left histogram) was the most affected since this class included OTUs found in very large proportion of reads that were more likely to propagate (‘bleed’) to other samples via tag error/jump. Hence, in this regard, abundance profiles are in our opinion a much more robust depiction of OTUs actually present in a given sample.

Currently, tag-jumps represent a non-negligible issue of the metabarcoding approach. Indeed, Esling et al. [28] demonstrated that libraries prepared with single index primers (as done here) and saturated double index primers result in undetectable read cross-contamination. This is particularly problematic when presence-absence of OTUs (i.e. richness) is critical for the outcome of a particular study (e.g. keystone OTU, geographic variation). To mitigate cross-contamination, these authors showed that multiplexing samples following Latin Square designs can optimize mistag detection. Recently, Kitson et al. [48] also reported on the development of nested metabarcode tagging (2 × 2 indexes) that apparently lead to accurate sample/index tracking. Sequencing depth and evenness of the community found within a sample are also non-trivial issues affecting community profiles (richness and abundance) obtained via metabarcoding. On the one hand, samples sequenced evenly (i.e. normalized across samples for similar sequencing depth) may not show all diversity present for samples that are strongly dominated by a few taxa ‘masking’ low abundance OTUs [2]. On the other hand, samples sequenced unevenly (as done here) may falsely exhibit higher diversity simply because they were sequenced at greater depth, which promotes biodiversity recovery [91]. In spite of these concerns, we are confident that our deeply sequenced samples JP07 and FL02 truly harbor more diversity than FL01 and GM14, especially considering the dense endolithic colonization observed for these samples, and that the preparation of JP07 consisted in pooling subsamples of microfloras from multiple locations across the Ryukyus archipelago to specifically maximize biodiversity recovery (see “Methods” section). To further support the above, we subsampled JP07 and FL02 to a shallow sequencing depth of 20,000 reads as reported for FL01 and GM14 (Table 4). Following OTU clustering (not shown), we observed a ~2–3 fold higher diversity for JP07 and FL02 than for FL01 and GM14, namely 87 and 66 OTUs vs. 28 and 38 OTUs, respectively. We nonetheless recognize that sequencing FL01 and GM14 deeper could have retrieved some additional OTU diversity.

Overall, sequencing future projects with the now available 2 × 300 bp chemistries should greatly improve upon demultiplexing issues caused by index errors. In spite of the noted important read losses, our data set contained very high read redundancy and still comprised important sequencing noise (as seen from overly abundant singletons, doubletons and tripletons resulting from the deep sequencing of FL02 and JP07, Table 3) and thus included much sufficient data to assess our microfloras’ diversity. Using a combination of superior algorithms with different *de novo* clustering strategies (as recommended in [88]) such as UPARSE’s global and SWARM’s local threshold algorithms, allowed us to filter for high quality core OTUs produced by both pipelines. As a result of this dual-clustering approach, our OTUs are very close to biological sequences (i.e. contain 0, 1 or 2 incorrect bases as shown for clones and Illumina core OTUs obtained for JP07, our only sample sequenced with both methods, (see Additional file 13: Figure S6), in congruence with the ≤1 % error rate reported on a mock community for UPARSE alone [26]. Overall, the phylogenetic breadth documented by our *de novo* OTUs (prokaryotic and eukaryotic diversity), added to the negligible proportion of identified contaminants (i.e. non-*tuf*A, <1 % of filtered reads, Table 3), together demonstrate the specificity of our newly designed degenerate primers to target *tuf*A from phototrophs that represented the majority of retrieved OTUs (77 % overall) and mapped reads (>98 % overall) (Fig. 13 and Additional file 7: Table S6). The priming region of *env_tuf*AF (Fig. 4) actually overlaps with an extended stretch of codon insertion/deletions in numerous heterotrophs, which may further favor annealing, and therefore amplification, toward phototrophic diversity. For samples with very low phototroph content and/or containing inhibitors (i.e. secondary metabolites [105]), target capture via hybridization with *tuf*A probes may provide a valuable alternative to amplification, as well as enhance rare OTUs detection limits. Some studies have performed target capture metabarcoding, such as for instance Patel et al. [71], Denonfoux et al. [20] and more recently Dowle et al. [22].

### Endolithic Ulvophyceae

The molecular diversity and phylogenetic breadth of the boring microsiphons referred to as *Ostreobium* spp. had clearly been overlooked in the literature and lacked *tuf*A referencing (a single sequence—FJ535859 was available on GenBank^®^ prior to our study). We document for this form genus an impressive polyphyletic cryptic diversity encompassing an estimated 85–95 species-level entities (Fig. 7), for which we delimited five provisional families to facilitate the profiling of endolithic communities (Figs. 9, 13). These families exhibit molecular divergence comparable to conspicuous Bryopsidalean families that are well circumscribed morphologically (e.g. the Halimedaceae, the Caulerpaceae, etc., Fig. 9). Our phylogeny also reveals unresolved branches at the base of the ‘Maedaceae’ (Ostreobidineae) (Figs. 7, 8), which pending further sampling, may lead to the delimitation of additional families. In this endeavor, clarifying the molecular identity of the generitype *O.quecketti* [7] from its type locality (Le Croisic, Brittany, France) should allow recircumscription of the family Ostreobiaceae P.C. Silva (proposed in [70]; validated in [9]) toward establishing a more stable classification. We expect that sequencing of endolithic communities worldwide will further increase species diversity of euendolithic microsiphons. Finally, although field collections were focused on ‘*Ostreobium*’ spp., our assessment also recovered common euendolithic microfilamentous genera of the ‘Ulvales-Ulothrichales’, namely the Phaeophilaceae and Ulvellaceae, in culture and from cloning and metabarcoding (Fig. 7, Additional file 1: Table S1, Additional file 5: Table S5 and Additional file 8: Table S7).

### Database performance

Thorough analyses of our newly established database resources for *tuf*A permitted referencing the taxonomy of OTUs based on their phylogenetic position, as well as defining identity thresholds for their rapid annotation in the context of environmental biomonitoring studies via metabarcoding. Although the backbone of our phototroph tree was overall poorly resolved (Fig. 6, as in [19]), topological relationships obtained among phyla/classes with *tuf*A were congruent with currently accepted hypotheses of chloroplasts/rhodoplasts and secondary endosymbiotic lineages evolution [74], allowing visualization of the database and OTUs in a phylogenetically sound framework (Fig. 6 and Additional file 14: Figure S7). Relying on our divergence analyses, we were able to establish in silico (relaxed) and clade-based (conservative) thresholds for OTU annotation at high taxonomy levels (Class 84–86 %, Phylum 83–83 %, and Domain 77–82 %, Figs. 10, 11) and lower taxonomy levels in the Ulvophyceae (species 97–99 %, family 85–92 %, suborder 79–84 % (for the Bryopsidales) and order 77–79 %, Figs. 10, 11). The fact that high-level taxonomy thresholds exceeded lower-level ones (i.e. within the Ulvophyceae) reflects the combination of group-specific differences in *tuf*A’s evolutionary rate (e.g. due to genome architecture, or lineage diversification age), low biodiversity sampling in some clades, taxonomic discrepancies, and poorly resolved relationships (i.e. paraphyletic/polyphyletic classes and phyla). Overall, our database achieved matching of OTUs at high identity values (the majority matching at >85 %, Fig. 12), which as a single threshold holds the potential to accurately annotate OTUs to both family (for Ulvophyceae representatives) and class-levels (all phototrophs). Here, the majority of hits below 85 % belonged to the class Cyanophyceae (not shown), which as the sole representative in the phylum Cyanophyta (and ‘Phototrophs’ in the prokaryotic domain) may thus be annotated with identity thresholds of 82 % (Fig. 10), and eventually further relaxed down to 77 % (Fig. 11). Overall, we recommend applying a range of thresholds (based on the above relaxed and conservative thresholds) and investigate with tree methods the phylogenetic position of OTUs that may cause profile disparities in order to correct their taxonomy. As demonstrated in the present study, our 375 bp metabarcode packs sufficient phylogenetic signal for model-based tree estimation when included in well-sampled sequence alignments (e.g. as implemented here within RAxML). Finally, matches in the 97–99 % range could be used for molecular species annotation in the Ulvophyceae (as estimated by GMYC or ABGD analyses, see Fig. 7 and Additional file 10: Figure S3), providing that profiling at this level becomes necessary and that a molecular-based taxonomy becomes available for this cryptic diversity (e.g. [44]).

### Community profile

In spite of a possible, minimal bias in richness profile introduced via tag error/jump, the profiling of the microfloras revealed a core assemblage of phyla, classes and some Ulvophycean families inhabiting endolithic communities, whose abundance varies, but appears universal (Fig. 13). Similarities in the phyla/classes richness and abundance profiles of the OTU-rich coral reef samples FL02 and JP07 are interesting considering their distant geographic origin (Pacific vs. Atlantic) and the fact that for the preparation of JP07, multiple CaCO_3_ samples collected from across the Ryukyu archipelago had been pooled together to maximize the recovery of endolithic biodiversity (see “Methods” section). In this regard, JP07 assemblage may thus be viewed as an average phyla/classes profile for shallow coral reefs of the Ryukyus (e.g. abundance of <5 % ‘Bacillariophyta’, <5 % Coccolithophyceae, <5 % Cyanophyceae, <20 % Florideophyceae and >65 % Ulvophyceae, and miscellaneous groups), whose applicability to other tropical areas such as in the Atlantic (FL02) would appear relevant. By contrast with these two samples, the skewed profiles of the less densely colonized microfloras FL01 and GM14 may reflect immature or developing communities undergoing succession following for example, recent niche opening or disturbance. The rhodolith microflora of GM14 was particularly Rhodophyta-rich from an abundant crust-forming *Rhizophyllis* sp. OTU (Florideophyceae) and an early branching OTU that we could not presently resolve within the phylum (Fig. 13, Additional file 11: Figure S4). Although we were careful to sample CaCO_3_ patches devoid of conspicuous encrusting red algae for the preparation of the microfloras’ DNA extracts, we cannot exclude that accidental drilling of *Rhizophyllis* (Rhizophyllidaceae) (present on the surface of GM14 upon this sample reexamination) could account for these reads rather than propagules of this taxon present endolithically. Likewise, the most abundant Rhodophyta in other microflora samples were also encrusting taxa, the families ‘Corallinaceae-Hapalidiaceae’ and Peyssonneliaceae (Florideophyceae), but other non-encrusting taxa were also present (in class Bangiophyceae, Compsopogonophyceae, Stylonematophyceae, and within the Florideophyceae, e.g. the order Ceramiales, and family Dumontiaceae, Additional file 7: Table S6, Additional file 8: Table S7). Lastly, GM14 also exhibited a lower phototroph/heterotroph ratio than other microfloras (Fig. 13), which may be explained by its depth of collection in the mesophotic zone (65 m) where light availability may limit photosynthesis and algal CaCO_3_ penetration more importantly than in shallow waters.

### Evolutionary perspectives

Our barcoding approach coupled with metabarcoding brought to light the widespread use of the endolithic niche as a habitat by the Bryopsidales (including otherwise epilithic taxa, see OTU and clones’ distribution in Fig. 7). For instance, several macroscopic taxa of the Bryopsidineae (e.g. Derbesiaceae, Bryopsidaceae) and the Halimedineae (e.g. Caulerpaceae, Halimedaceae, Rhipiliaceae, ‘Siphonogramenaceae and Pseudochlorodesmidaceae’) are present and may occupy the substratum in the form of germlings, reproductive cells, and endolithic siphonous/microsiphonous networks, the latter possibly used for lateral vegetative dispersal, thallus regeneration and access to nutrient-rich sediment trapped in cavities. Overall, the Bryopsidales seem to present a tight evolutionary link with the endolithic niche as an ancestral habitat, as evidenced by the early branching of the Ostreobidineae in multi-marker studies [102], also supported here with *tuf*A (bootstrap >95 %, Fig. 8). We hypothesize that environmental constraints associated with the epilithic niche such as high light/UV, direct water flow, and macroscopic predators (which themselves diversified and specialized over time, [16], such as kleptoplastic sacoglossan sea slugs, [39]) may have sparked the diversification of morphologically complex and conspicuous families from ancestral microsiphons living at the interface between the water column and the endolithic niche. In epilithic families able to exploit the endolithic niche with networks of siphons, this feature may thus represent a symplesiomorphy for some (e.g. the ‘Siphonogramenaceae and ‘Pseudochlorodesmidaceae’) and a possibly re-acquired trait for others (i.e. the *Caulerpa* ‘*ambigua*’ complex in the Caulerpaceae) as suggested from the long branches leading to such lineage (i.e. accelerated evolution following key innovation into a new adaptive zone, Simpson [92, 93]) (Figs. 7, 8). Aside from the Bryopsidales, the phylogenetic extent of phototrophic OTUs also found in the endolithic niche seemingly in the form of chaesmo- and cryptoendolithic reproductive cells or germlings, perennial boring euendoliths (endolithic Ulvophyceae) as well as transient ones stages (e.g. some alternate life history stages of the Florideophyceae, Rhodophyta), highlights the critical importance of the CaCO_3_ substratum in algal evolution as a seedbank for life cycle completion and survival of diverse algal taxa [30, 32].

## Conclusions

In summary, we provide a flexible and comprehensive metabarcoding framework including primers, reference data and annotation thresholds for the facilitated recovery and rapid profiling of phototrophs found in endolithic communities. As a new resource for environmental biomonitoring, the framework is timely to enable the use of high throughput sequencing to accelerate biodiversity characterization of microbial/algal assemblages from endolithic communities found in coral reef and rhodolith ecosystems in the context of global change studies (e.g. bioerosion, distributional shifts), holobiont functioning and anthropogenic degradation (e.g. eutrophication, overfishing). Our framework could also find useful applications for water quality studies related to public health, such as the monitoring of river eutrophication based on the Diatom Index (i.e. composition of the ‘Bacillariophyta’ [47, 104]) and the detection of toxic Cyanobacteria (i.e. the Cyanophyceae) in freshwater and coastal systems [4, 6] pending further curation and increase in reference data for these particular groups, which our primers retrieved very efficiently. Overall, *tuf*A metabarcoding assays could be performed on numerous types of samples harboring algal phototrophs including for instance water column, biofilms, periphyton, soil and ice samples and herbivorous organisms (e.g. vertebrate/invertebrate stomach contents, kleptoplastidic slugs tissue). Further studies of endolithic communities in coral reef and rhodolith ecosystems may reveal potential bioindicators of ecosystem degradation (taxonomic group/OTU ratio), whose efficient monitoring and detection may allow better management and conservation practices.

## Additional files

10.1186/s12898-016-0068-x Barcoded Ulvophyceae. Collection information for Ulvophyceae specimens newly sequenced for *tuf*A. To ease public database searches, Genbank^®^ identifiers include both morphological and molecular identification (in brackets []) at the genus or family level (when abbreviated). Polyphyletic genera, species complexes or combined orders indicated in between quotes. All specimens from shallow waters (<0–5 m) otherwise indicated in footnotes. Endolithic habitat abbreviated as follows: *CaCO*_*3*_ open reef substratum, *Corall.* Florideophycean crusts of the order Corallinales, *Peyss.* Florideophycean crusts of the order Peyssonneliales, *LPS* large polyp coral species (e.g. *Galaxea* sp., *Favia* sp.), *Shell* Oyster shell (primarily) or other bivalve, *SP* *S* small polyp coral species (e.g. *Porites* sp., *Montipora* sp.), *na* epilithic, epiphytic, or not recorded. Collector (Coll.) initials as follows: DP = D. Pence, HS = H. Spalding, JR = J. Richards, KI = K. Ikemoto, MDR = M. Diaz-Ruiz, MS = M. Star, NP = N. Pyron, TS = T. Sauvage, WES = W.E. Schmidt, ZM = Z. McCorkhill. Other abbreviations: Cult. = Cultured, IUI = Interuniversity Institute for Marine Science, INVEMAR = Instituto de investigaciones marinas y costeras.
10.1186/s12898-016-0068-x Barcoded Florideophyceae, Phaeophyceae and miscellaneous taxa. Collection information for specimens of the Florideophyceae and Phaeophyceae sequenced for *tuf*A in the present study (as well as a miscellaneous Prasinophyceae). Polyphyletic orders or family and combined orders are indicated in between quotes. All specimens from shallow waters (0–10 m) otherwise indicated in footnotes. Collector (Coll.) initials as follows: CP = C. Pueschel, CS = C. Stoude, DG = D. Gabriel, DK = D. Krayesky, DWF = D.W. Freshwater, EC = E. Coppejans, ED = E. Deslandes, JC = J. Cabioch, JH = J. Hughey, JR = J. Richards, JRu = J. Rueness, JZ = J. Zertuche, MG = M. Guiry, MHH = M. H. Hommersand, MJW = M. J. Wynne, MY = M. Yoshizaki, OC = O. Camacho, SF = S. Fredericq.
10.1186/s12898-016-0068-x Indexed forward primers used for metabarcoding. Indexes in bold.
10.1186/s12898-016-0068-x Summary taxonomy and content of the non-redundant *tuf*A database. Diversity and sequence counts for (A) Heterotrophs and Phototrophs, (B) Phyla and Classes for all phototrophs and (C) Orders/suborders and families for the Ulvophyceae (Genus taxonomy not shown for conciseness). Taxonomic groupings with unsettled naming such as combined orders, provisional families, and polyphyletic families (i.e. ‘Kornamanniaceae’ and ‘Ulothrichaceae’) are noted between quotes.
10.1186/s12898-016-0068-x Clone taxonomy. Detailed reference taxonomy for 86 phototrophic clones from microfloras sampled in the Ryukyu archipelago (JP01, JP03, JP04, JP06, JP07, JP25), an aquarium window scrap (E09) and a Gulf of Mexico crustose coralline *Lithophyllum* sp. rhodolith. Taxonomic groupings with unsettled naming such as combined orders, provisional families, and polyphyletic or monophyletic species or genera complexes, are noted between quotes.
10.1186/s12898-016-0068-x Cumulative OTU-ranked abundance curves. Note the much larger OTU diversity in densely colonized JP07 and FL02 vs. lightly colonized microfloras FL01 and GM14. Cumulative abundances values below 80 % not displayed for figure succinctness.
10.1186/s12898-016-0068-x Summary of microfloras richness and abundance at multiple taxonomic levels. (A–B) OTU counts and read abundance for ‘Phototrophs’ vs. ‘Heterotrophs’ group. (C–D) OTU counts and read abundance for phototrophic phyla and classes. (E–F) OTU counts and read abundance for Ulvophyceaen families. Note that OTUs may be found more than once across samples.
10.1186/s12898-016-0068-x OTUs taxonomy and their microflora assignment. Detailed reference taxonomy for 802 core OTUs as determined from their phylogenetic position (see Fig. 6). Taxonomic groupings with unsettled naming such as combined orders, provisional families, and polyphyletic or monophyletic species or genera complexes, are noted between quotes.
10.1186/s12898-016-0068-x Overlapping OTU diversity. Venn diagram depicting common OTU diversity (i.e. at the circles’ intersection) and unique OTUs in each of FL02 and JP07 for all phototrophs and several OTU-rich classes. Overlapping OTUs may represent diversity that is both truly found in both samples (i.e. pantropical) and resulting from false assignment caused by index tag jumping or tag error (see Additional file 12: Figure S5), which may potentially inflate richness estimates. Note, however, the still important OTU richness unique to both FL02 and JP07, especially the OTU-rich classes Cyanophyceae and ‘Bacillariophyta’.
10.1186/s12898-016-0068-x Molecular species divergence in the Ulvophyceae. Distribution of intra- and interspecific divergence for molecular species of the orders Bryopsidales and ‘Ulvales-Ulothrichales’ delimited with ABGD and GMYC. Percent identity values were computed based on an alignment of 375 bp of the *tuf*A metabarcode. Maximum thresholds for molecular species classification/annotation are printed. *PI* percent identity.
10.1186/s12898-016-0068-x OTU abundance profiles. OTUs underlying abundance patterns in Fig. 13. OTUs found in large read abundance across multiple microflora samples are color-coded in blue, green, orange and red. Other abundant OTUs are color-coded in white and those below 2 % abundance in shades of grey. The OTUs low-level taxonomy and their percent match to the database are listed in the legend.
10.1186/s12898-016-0068-x Example of putative index tag jumping or tag error. Comparative read abundance for seven OTUs demultiplexed (i.e. assigned) to both FL02 and JP07. For illustration purpose, only OTUs with >5 % of mapped reads in either FL02 or JP07 are shown (these correspond to seven out of the 141 common OTUs, see Additional file 9: Figure S2). Note the drastic abundance disparity illustrative of or potential of false assignment via index tag jumping or tag error (e.g. OTU 511 representing ≪0.1 % reads in FL02 and 10.4 % of reads in JP07). By contrast, note the more balanced abundance of OTU 355, 356, and 506, which may reflect the true presence of common OTUs in both samples (i.e. pantropical OTUs).
10.1186/s12898-016-0068-x OTUs vs. clone identity and coverage. Comparison of all phototrophic clones obtained from JP07 with their matching OTUs. Note high identity of clones and OTUs based on 375 bp, with 0, 1 or 2 base pair variation (100, 99.7 and 99.5 % respectively). Also note that clone H and its corresponding OTU_364 share the highest coverage, although further cloning would be necessary to accurately assess congruence in sequence coverage between the two methods.
10.1186/s12898-016-0068-x *tuf*A database and higher taxonomy overview. Unrooted RAxML tree comprising 556 ‘Heterotrophs’and 2141 ‘Phototrophs’ *tuf*A sequences aligned on 891 bp. ‘Heterotrophs’ include multiple phyla of the Eubacteria. ‘Phototrophs’ include the prokaryotic eubacterial phylum Cyanophyta and several eukaryotic phyla, including the Archaeplastida (Chlorophyta, Glaucophyta and Rhodophyta) and secondary endosymbiotic phyla. ‘Chloroplasts’ include the Chlorophyta and the secondary endosymbiotic phyla Euglenophyta and Chlorarachniophyta. ‘Rhodoplasts’ include the Rhodophyta and the secondary endosymbiotic phyla of the Cryptophyta, Haptophyta and Ochrophyta.

## Abbreviations

ABGD: automatic barcode gap discovery
bp: base pairs
CaCO_3_: calcium carbonate
CCA: crustose coralline algae
EF-1a: elongation factor 1 alpha
ESS: effective sample size
GeO_2_: germanium dioxide
GMYC: general mixed yule coalescence
ITS: internal transcribed spacer
LAF: University of Louisiana at Lafayette
LSU: large subunit
MCMC: Markov Chain Monte Carlo
NCBI: National Center for Biotechnology Information
pCO_2_: partial pressure of carbon dioxide
PCR: polymerase chain reaction
PI: percent identity
OTU: Operational Taxonomic Unit
*rbc*L: RuBisCO large subunit
RDP: ribosomal database project
sp., spp.: species
*tuf*A: elongation factor EF-*Tu*
*tuf*B: elongation factor EF-*Tu* 2
UPA: universal plastid amplicon
