# Supplementary material for: A metabarcoding framework for facilitated survey of endolithic phototrophs with tufA
Source: BMC Ecol. 2016 Mar 10;16:8. doi: 10.1186/s12898-016-0068-x (PMC4785743; doi:10.1186/s12898-016-0068-x)
Supplement: Supplementary file 1 — 10.1186/s12898-016-0068-x Barcoded Ulvophyceae. Collection information for Ulvophyceae specimens newly sequenced for tufA. To ease public database searches, Genbank® identifiers include both morphological and molecular identification (in brackets []) at the genus or family level (when abbreviated). Polyphyletic genera, species complexes or combined orders indicated in between quotes. All specimens from shallow waters (<0–5 m) otherwise indicated in footnotes. Endolithic habitat abbreviated as follows: CaCO 3 open reef substratum, Corall. Florideophycean crusts of the order Corallinales, Peyss. Florideophycean crusts of the order Peyssonneliales, LPS large polyp coral species (e.g. Galaxea sp., Favia sp.), Shell Oyster shell (primarily) or other bivalve, SP S small polyp coral species (e.g. Porites sp., Montipora sp.), na epilithic, epiphytic, or not recorded. Collector (Coll.) initials as follows: DP = D. Pence, HS = H. Spalding, JR = J. Richards, KI = K. Ikemoto, MDR = M. Diaz-Ruiz, MS = M. Star, NP = N. Pyron, TS = T. Sauvage, WES = W.E. Schmidt, ZM = Z. McCorkhill. Other abbreviations: Cult. = Cultured, IUI = Interuniversity Institute for Marine Science, INVEMAR = Instituto de investigaciones marinas y costeras. [file 12898_2016_68_MOESM1_ESM.pdf]

GenBank	Genbank Identifier	Collection #	Order	Suborder	Family	Cult.	Geographical origin	Year	Coll.	GPS coordinates	Habitat
KU361834	Pseudobryopsis oahuensis	TS0066	Bryopsidales	aff. Bryopsidineae	Unresolved		Kualoa Beach Park, Oahu, Hawaii, USA	2004	KI	21°30'48"N 157°50'13"W	na
KU361835	Unres. Bryopsidineae/[aff.Bryops.]	TS1150	Bryopsidales	Bryopsidineae	aff_Bryopsidaceae	X	Coral Beach, Eilat, Israel	2011	TS	29°30'13"N 34°55'08"E	na
KU361836	Unres. Bryopsidineae/[aff.Bryops.]	TS1442	Bryopsidales	Bryopsidineae	aff_Bryopsidaceae		Uganzaki, Ishigaki, Japan	2012	TS	24°21'70"N 124°04'51"E	na
KU361837	Unres. Bryopsidineae/[Bryops.]	TS0059/ARS1788	Bryopsidales	Bryopsidineae	Bryopsidaceae		Kahala, Oahu, Hawaii, USA	2006	ZM	21°16'12"N 21°16'12"N	na
KU361838	Unres. Bryopsidineae/[Bryops.]	TS0395	Bryopsidales	Bryopsidineae	Bryopsidaceae	X	Waikiki, Oahu, Hawaii, USA	2009	TS	21°15'50"N 157°49'24"W	na
KU361839	Bryopsis sp.	TS1192	Bryopsidales	Bryopsidineae	Bryopsidaceae		Mibaru Beach, Okinawa, Japan	2012	TS	26°07'53"N 127°47'03"E	na
KU361840	Unres. Bryopsidineae/[Pseudoderbesia sp.][2]	TS1915	Bryopsidales	Bryopsidineae	Bryopsidaceae		Aquarium, Ewing Bank, offshore Louisiana, USA		TS	--	na
KU361841	Unres. Bryopsidineae [Codium sp.][2]	TS1099	Bryopsidales	Bryopsidineae	Codiaceae		Aquarium, Ewing Bank, offshore Louisiana, USA	2011	TS	--	na
KU361842	Unres. Bryopsidineae [Codium sp.][3]	TS1111	Bryopsidales	Bryopsidineae	Codiaceae	X	Aquarium, Ewing Bank, offshore Louisiana, USA	2011	TS	--	na
KU361843	Codium sp.	TS1230	Bryopsidales	Bryopsidineae	Codiaceae		Hamahiga, Okinawa, Japan	2012	TS	26°19'12"N 127°57'59"E	na
KU361844	Codium sp.	TS1231	Bryopsidales	Bryopsidineae	Codiaceae		Hamahiga, Okinawa, Japan	2012	TS	26°19'12"N 127°57'59"E	na
KU361845	Codium sp.	TS1286	Bryopsidales	Bryopsidineae	Codiaceae		Odo, Okinawa, Japan	2012	TS	26°05'21"N 127°42'38"E	na
KU361846	Codium sp.	TS1307	Bryopsidales	Bryopsidineae	Codiaceae		Uganzaki, Ishigaki, Japan	2012	TS	24°21'70"N 124°04'51"E	na
KU361847	Codium sp.	TS1310	Bryopsidales	Bryopsidineae	Codiaceae		Maesato, Ishigaki, Japan	2012	TS	24°19'54"N 124°11'00"E	na
KU361848	Codium sp.	TS1311	Bryopsidales	Bryopsidineae	Codiaceae		Maesato, Ishigaki, Japan	2012	TS	24°19'54"N 124°11'00"E	na
KU361849	Codium sp.	TS1462	Bryopsidales	Bryopsidineae	Codiaceae		Aragusuku, Miyako, Japan	2012	TS	24°45'37"N 125°25'30"E	na
KU361850	Unres. Bryopsidineae [Codium sp.][3]	TS1668	Bryopsidales	Bryopsidineae	Codiaceae		Aquarium, Ewing Bank, offshore Louisiana, USA	2012	TS	--	na
KU361851	Derbesia sp.	ARS0602	Bryopsidales	Bryopsidineae	Derbesiaceae	X	Oahu, Hawaii, USA	2005	TS	--	na
KU361852	Derbesia sp.	TS0394	Bryopsidales	Bryopsidineae	Derbesiaceae		Offshore, Oahu, Hawaii, USA	--	HS	21°15'04"N 158°03'02"W	na
KU361853	Derbesia sp.	TS0974	Bryopsidales	Bryopsidineae	Derbesiaceae	X	Eilat coast (na), Israel	2011	TS	--	na
KU361854	Derbesia sp.	TS1380	Bryopsidales	Bryopsidineae	Derbesiaceae	X	Unarizaki, Iriomote, Japan	2012	TS	24°25'33"N 123°45'56"E	[SPS]
KU361855	Derbesia sp.	TS1495	Bryopsidales	Bryopsidineae	Derbesiaceae		Ueno, Miyako, Japan	2012	TS	24°43'28"N 125°21'33"E	[SPS]
KU361856	Derbesia sp.	TS1497	Bryopsidales	Bryopsidineae	Derbesiaceae		Ueno, Miyako, Japan	2012	TS	24°43'28"N 125°21'33"E	[SPS]
KU361857	Derbesia sp.	TS1526	Bryopsidales	Bryopsidineae	Derbesiaceae	X	Ikema Island, Miyako, Japan	2012	TS	24°55'53"N 125°13'59"E	[Corall.]
KU361858	Derbesia sp.	TS1596	Bryopsidales	Bryopsidineae	Derbesiaceae		Aragusuku, Miyako, Japan	2012	TS	24°45'37"N 125°25'30"E	[LPS]
KU361859	Derbesia sp.	TS1731	Bryopsidales	Bryopsidineae	Derbesiaceae	X	Walton Rocks Beach, Florida, USA	2013	TS	27°20'17"N 80°13'59"W	na
KU361860	Derbesia sp.	TS1759	Bryopsidales	Bryopsidineae	Derbesiaceae		Fort Pierce Inlet, Florida, USA	2013	TS	27°28'14"N 80°17'19"W	na
KU361861	Derbesia sp.	TS1898	Bryopsidales	Bryopsidineae	Derbesiaceae	X	Jupiter Lagoon, Florida, USA	2013	TS	26°57'24"N 80°04'41"W	na
KU361862	Derbesia sp.	TS1899	Bryopsidales	Bryopsidineae	Derbesiaceae	X	Jupiter Lagoon, Florida, USA	2013	TS	26°57'24"N 80°04'41"W	na
KU361863	Unres. Bryopsidineae[1]	TS0400	Bryopsidales	Bryopsidineae	Unresolved		Off Ala Moana Beach Park, Oahu, Hawaii, USA	2009	DP	21°16'54"N 157°51'15"W	na
KU361864	Unres. Bryopsidineae	TS1107	Bryopsidales	Bryopsidineae	Unresolved	X	Eilat, Israel	2011	TS	29°29'51"N 34°54'41"E	na
KU361865	Unres. Bryopsidineae	TS1217A	Bryopsidales	Bryopsidineae	Unresolved	X	Maeda, Okinawa, Japan	2012	TS	26°26'35"N 127°46'06"E	[LPS]
KU361866	Unres. Bryopsidineae	TS1638	Bryopsidales	Bryopsidineae	Unresolved	X	Chatan, Okinawa, Japan	2012	TS	26°19'43"N 127°44'38"E	na
KU361867	Unres. Bryopsidineae	TS1684	Bryopsidales	Bryopsidineae	Unresolved		Smithsonian Aquarium Museum, Florida, USA	--	TS	--	na
KU361868	Unres. Bryopsidineae	TS1780	Bryopsidales	Bryopsidineae	Unresolved		Jupiter, Florida, USA	2013	TS	26°57'51"N 80°04'40"W	na
KU361869	Unres. Bryopsidineae	TS1900	Bryopsidales	Bryopsidineae	Unresolved	X	Jupiter Reef, Florida, USA	2013	TS	26°57'51"N 80°04'41"W	na
KU361870	Unres. Bryopsidineae[2]	TS1903	Bryopsidales	Bryopsidineae	Unresolved	X	Aquarium, Ewing Bank, offshore Louisiana, USA	2013	TS	--	na
KU361871	Flabellia petiolata	TS0542	Bryopsidales	Halimedineae	aff_Udoteaceae		Cap d'Antibes, France	2010	TS	43°33'06"N 7°07'10"E	na
KU361872	Tydemania expeditionis	TS1168	Bryopsidales	Halimedineae	aff_Udoteaceae		Tokashki, Kerama Islands, Okinawa, Japan	2012	TS	26°10'09"N 127°20'33"E	na
KU361873	Caulerpa 'ambigua'	TS0481	Bryopsidales	Halimedineae	Caulerpaceae		Playa del Rodadero, Santa Marta, Colombia	2010	TS	11°12'01"N 74°13'43"W	na
KU361874	Caulerpa 'ambigua'	TS1263	Bryopsidales	Halimedineae	Caulerpaceae		Hamahiga, Okinawa, Japan	2012	TS	26°19'12"N 127°57'59"E	na
KU361875	Caulerpa 'ambigua'	TS1341	Bryopsidales	Halimedineae	Caulerpaceae		Unarizaki, Iriomote, Japan	2012	TS	24°25'33"N 123°45'56"E	na
KU361876	'Pseudochlorodesmis' sp./[Caulerpa sp.]	TS1639	Bryopsidales	Halimedineae	Caulerpaceae		Chatan, Okinawa, Japan	2012	TS	26°19'43"N 127°44'38"E	na
KU361877	Caulerpa 'ambigua'	TS1754	Bryopsidales	Halimedineae	Caulerpaceae		Fort Pierce Inlet, Florida, USA	2013	TS	27°28'14"N 80°17'19"W	na
KU361878	Caulerpa 'ambigua'	TS1794	Bryopsidales	Halimedineae	Caulerpaceae		BathTub beach, Florida, USA	2013	TS	27°11'06"N 80°09'35"W	na
KU361879	Avrainvillea sp.	TS1256	Bryopsidales	Halimedineae	Dichotomosiphonaceae		Hamahiga, Okinawa, Japan	2012	TS	26°19'12"N 127°57'59"E	na
KU361880	Avrainvillea sp.	TS1330	Bryopsidales	Halimedineae	Dichotomosiphonaceae		Sonai, Iriomote, Japan	2012	TS	24°23'20"N 123°44'45"E	na
KU361881	'Pseudochlorodesmis' sp./[Avrainvillea sp.]	TS1504	Bryopsidales	Halimedineae	Dichotomosiphonaceae	X	Sonai, Iriomote, Japan	2012	TS	24°23'20"N 123°44'45"E	na
KU361882	Avrainvillea sp.	TS1819	Bryopsidales	Halimedineae	Dichotomosiphonaceae		Venture Key, Florida, USA	2013	TS	24°38'46"N 81°27'59"W	na
KU361883	'Pseudochlorodesmis' sp./[Avrainvillea sp.]	TS1943	Bryopsidales	Halimedineae	Dichotomosiphonaceae	X	Florida Keys, Florida, USA	--	TS	--	na
KU361884	'Pseudochlorodesmis' sp./[Halimeda sp.]	TS0399	Bryopsidales	Halimedineae	Halimedaceae	X	Makai Pier, Oahu, Hawaii, USA	2009	TS	21°19'11"N 157°40'10"W	na
KU361885	'Pseudochlorodesmis' sp./[Halimeda sp.]	TS0477	Bryopsidales	Halimedineae	Halimedaceae		INVEMAR Aquarium, Santa Marta, Colombia	2010	MDR	--	na
KU361886	Halimeda sp.	TS1244	Bryopsidales	Halimedineae	Halimedaceae		Hamahiga, Okinawa, Japan	2012	TS	26°19'12"N 127°57'59"E	na
KU361887	Halimeda discoidea	TS1245	Bryopsidales	Halimedineae	Halimedaceae		Hamahiga, Okinawa, Japan	2012	TS	26°19'12"N 127°57'59"E	na
KU361888	Halimeda sp.	TS1246	Bryopsidales	Halimedineae	Halimedaceae		Hamahiga, Okinawa, Japan	2012	TS	26°19'12"N 127°57'59"E	na
KU361889	Halimeda borneensis	TS1247	Bryopsidales	Halimedineae	Halimedaceae		Hamahiga, Okinawa, Japan	2012	TS	26°19'12"N 127°57'59"E	na
KU361890	Halimeda sp.	TS1355	Bryopsidales	Halimedineae	Halimedaceae		Haemida, Iriomote, Japan	2012	TS	24°16'15"N 123°49'52"E	na
KU361891	Halimeda sp.	TS1444	Bryopsidales	Halimedineae	Halimedaceae		Ikema Island, Miyako, Japan	2012	TS	24°55'53"N 125°13'59"E	na

GenBank	Genbank Identifier	Collection #	Order	Suborder	Family	Cult.	Geographical origin	Year	Coll.	GPS coordinates	Habitat
KU361892	Halimeda discoidea	TS1448	Bryopsidales	Halimedineae	Halimedaceae		Kurima Island, Miyako, Japan	2012	TS	24°43'03"N 125°14'24"E	na
KU361893	Halimeda fragilis	TS1456	Bryopsidales	Halimedineae	Halimedaceae		Kurima Island, Miyako, Japan	2012	TS	24°43'03"N 125°14'24"E	na
KU361894	Halimeda sp.	TS1457	Bryopsidales	Halimedineae	Halimedaceae		Kurima Island, Miyako,Japan	2012	TS	24°43'03"N 125°14'24"E	na
KU361895	Halimeda opuntia	TS1460	Bryopsidales	Halimedineae	Halimedaceae		Aragusuku, Miyako, Japan	2012	TS	24°45'37"N 125°25'30"E	na
KU361896	Halimeda sp.	TS1461	Bryopsidales	Halimedineae	Halimedaceae		Aragusuku, Miyako, Japan	2012	TS	24°45'37"N 125°25'30"E	na
KU361897	'Pseudochlorodesmis' sp./[Halimeda sp.][1]	TS1906	Bryopsidales	Halimedineae	Halimedaceae		Garden Key, Dry Tortugas, Florida, USA	2014	TS	24°37'47"N 82°52'17"W	[CaCO3]
KU361898	'Pseudochlorodesmis' sp./[Pseudochlo.]	TS0250	Bryopsidales	Halimedineae	'Pseudochlorodesmidaceae'		Paiko Lagoon, Oahu, Hawaii, USA	2008	TS	21°16'39"N 157°43'25"W	na
KU361899	'Pseudochlorodesmis' sp./[Pseudochlo.]	TS0539	Bryopsidales	Halimedineae	'Pseudochlorodesmidaceae'		Cap d'Antibes, France	2010	TS	43°33'06"N 7°07'10"E	na
KU361900	'Pseudochlorodesmis' sp./[Pseudochlo.]	TS0540	Bryopsidales	Halimedineae	'Pseudochlorodesmidaceae'		Cap d'Antibes, France	2010	TS	43°33'06"N 7°07'10"E	na
KU361901	'Pseudochlorodesmis' sp./[Pseudochlo.]	TS1523	Bryopsidales	Halimedineae	'Pseudochlorodesmidaceae'		Unarizaki, Iriomote, Japan	2012	TS	24°25'33"N 123°45'56"E	na
KU361902	'Pseudochlorodesmis' sp./[Pseudochlo.]	TS1650	Bryopsidales	Halimedineae	'Pseudochlorodesmidaceae'		Chatan, Okinawa, Japan	2012	TS	26°19'43"N 127°44'38"E	na
KU361903	'Pseudochlorodesmis' sp./[Pseudochlo.]	TS1697	Bryopsidales	Halimedineae	'Pseudochlorodesmidaceae'		Jupiter, Florida, USA	2013	TS	26°57'09"N 80°04'41"W	na
KU361904	'Ostreobium' sp./[Pseudostreob.][3]	L38	Bryopsidales	Halimedineae	'Pseudostreobiaceae'		Offshore, Maui, Hawaii, USA	2011	HS	20°45'56N 156°40'12W	[SPS]
KU361905	'Ostreobium' sp./[Pseudostreob.]	TS1498	Bryopsidales	Halimedineae	'Pseudostreobiaceae'		Ueno, Miyako, Japan	2012	TS	24°43'28"N 125°21'33"E	[SPS]
KU361906	'Pseudochlorodesmis' sp./[Pseudostreob.]	TS1552	Bryopsidales	Halimedineae	'Pseudostreobiaceae'		Sesoko Island, Okinawa, Japan	2012	TS	26°39'00"N 127°52'25"E	[CaCO3]
KU361907	'Ostreobium' sp./[Pseudostreob.]	TS1553A	Bryopsidales	Halimedineae	'Pseudostreobiaceae'		Sesoko Island, Okinawa, Japan	2012	TS	26°39'00"N 127°52'25"E	[SPS]
KU361908	'Ostreobium' sp./[Pseudostreob.]	TS1569	Bryopsidales	Halimedineae	'Pseudostreobiaceae'		Ikema Island, Miyako, Japan	2012	TS	24°55'53"N 125°13'59"E	[LPS]
KU361909	'Ostreobium' sp./[Pseudostreob.]	TS1570	Bryopsidales	Halimedineae	'Pseudostreobiaceae'		Ikema Island, Miyako, Japan	2012	TS	24°55'53"N 125°13'59"E	[LPS]
KU361910	'Ostreobium' sp./[Pseudostreob.]	TS1587	Bryopsidales	Halimedineae	'Pseudostreobiaceae'		Akashi, Ishigaki, Japan	2012	TS	24°32'15"N 124°18'15"E	[CaCO3]
KU361911	'Pseudochlorodesmis' sp./[Rhip.]	LAF6030	Bryopsidales	Halimedineae	Rhipiliaceae		El Tor, Egypt	2012	TS	28°14'04"N 33°36'10"E	na
KU361912	'Pseudochlorodesmis' sp./[Rhip.]	TS1149	Bryopsidales	Halimedineae	Rhipiliaceae	X	Coral Beach, Eilat, Israel	2011	TS	29°30'13"N 34°55'08"E	na
KU361913	'Pseudochlorodesmis' sp./[Rhip.]	TS1264	Bryopsidales	Halimedineae	Rhipiliaceae		Hamahiga, Okinawa, Japan	2012	TS	26°19'12"N 127°57'59"E	na
KU361914	'Pseudochlorodesmis' sp./[Rhip.]	TS1338	Bryopsidales	Halimedineae	Rhipiliaceae		Sonai, Iriomote, Japan	2012	TS	24°23'20"N 123°44'45"E	na
KU361915	Rhipilia sp.	TS1467	Bryopsidales	Halimedineae	Rhipiliaceae		Ikema Island, Miyako, Japan	2012	TS	24°55'53"N 125°13'59"E	na
KU361916	'Pseudochlorodesmis' sp./[Rhip.]	TS1470	Bryopsidales	Halimedineae	Rhipiliaceae		Ikema Island, Miyako, Japan	2012	TS	24°55'53"N 125°13'59"E	na
KU361917	Rhipilia sp.	TS1483	Bryopsidales	Halimedineae	Rhipiliaceae		Kurima Island, Miyako, Japan	2012	TS	24°43'03"N 125°14'24"E	na
KU361918	Rhipilia sp.[1]	TS1920	Bryopsidales	Halimedineae	Rhipiliaceae		Aquarium, Offshore Dry Tortugas, USA	2012	TS	--	na
KU361919	'Pseudochlorodesmis' sp./[Rhip.][1]	TS1942	Bryopsidales	Halimedineae	Rhipiliaceae	X	Gulf of Mexico	--	TS	--	na
KU361920	'Pseudochlorodesmis' sp./[Siphono.]	LAF6031	Bryopsidales	Halimedineae	'Siphonogramenaceae'		El Tor, Egypt	2012	TS	28°14'04"N 33°36'10"E	na
KU361921	'Pseudochlorodesmis' sp./[Siphono.][2]	TS1098	Bryopsidales	Halimedineae	'Siphonogramenaceae'	X	Aquarium, Ewing Bank, offshore Louisiana, USA	2011	TS	--	na
KU361922	'Pseudochlorodesmis' sp./[Siphono.][2]	TS1144	Bryopsidales	Halimedineae	'Siphonogramenaceae'	X	Aquarium, Ewing Bank, offshore Louisiana, USA	2011	TS	--	na
KU361923	'Pseudochlorodesmis' sp./[Siphono.][2]	TS1151	Bryopsidales	Halimedineae	'Siphonogramenaceae'	X	Aquarium, Ewing Bank, offshore Louisiana, USA	2011	TS	--	na
KU361924	'Pseudochlorodesmis' sp./[Siphono.]	TS1918	Bryopsidales	Halimedineae	'Siphonogramenaceae'		El Tor, Egypt	2012	WES	28°14'04"N 33°36'10"E	[Corall.]
KU361925	Poropsis sp.	ARS2753	Bryopsidales	Halimedineae	Udoteaceae		Honolua Bay, Maui, Hawaii, USA	2007	TS	21°00'50"N 156°38'19"W	na
KU361926	Penicillus capitatus	TFP08_27/TS0551	Bryopsidales	Halimedineae	Udoteaceae		STRI, Bocas del Toro, (Caribbean) Panama	2008	TS	9°21'04"N 82°15'25"W	na
KU361927	Espera/[Penicillus capitatus]	TFP08_429/TS0552	Bryopsidales	Halimedineae	Udoteaceae		STRI, Bocas del Toro, (Caribbean) Panama	2008	TS	9°21'04"N 82°15'25"W	na
KU361928	Poropsis sp.	TS0090/ARS1457	Bryopsidales	Halimedineae	Udoteaceae		Kahala, Oahu, Hawaii, USA	2006	TS	21°15'35"N 157°47'14"W	na
KU361929	'Rhipidosiphon' sp.	TS0097/ARS1649	Bryopsidales	Halimedineae	Udoteaceae		Oahu, Hawaii, USA	2006	TS	--	na
KU361930	'Rhipidosiphon' sp.	TS0392	Bryopsidales	Halimedineae	Udoteaceae		Waikiki, Oahu, Hawaii, USA	2009	TS	21°15'50"N 157°49'24"W	na
KU361931	Poropsis sp.	TS0397	Bryopsidales	Halimedineae	Udoteaceae		Hamoa Bay, Maui, Hawaii, USA	2009	TS	20°43'08"N 155°59'11"W	na
KU361932	Udotea sp.	TS0398	Bryopsidales	Halimedineae	Udoteaceae		Offshore, Oahu, Hawaii, USA	--	HS	21°15'04"N 158°03'02"W	na
KU361933	Poropsis sp.	TS1097	Bryopsidales	Halimedineae	Udoteaceae		Hosh Hanikra South, Israel	2011	TS	33°02'33"N 35°05'57"E	na
KU361934	'Rhipidosiphon' sp.	TS1179	Bryopsidales	Halimedineae	Udoteaceae		Tokashki, Kerama Islands, Okinawa, Japan	2012	TS	26°10'09"N 127°20'33"E	na
KU361935	'Rhipidosiphon' sp.	TS1181	Bryopsidales	Halimedineae	Udoteaceae		Tokashki, Kerama Islands, Okinawa, Japan	2012	TS	26°12'07"N 127°22'20"E	na
KU361936	'Rhipidosiphon' sp.	TS1224	Bryopsidales	Halimedineae	Udoteaceae		Maeda, Okinawa, Japan	2012	TS	26°26'35"N 127°46'06"E	na
KU361937	Chlorodesmis sp.	TS1241	Bryopsidales	Halimedineae	Udoteaceae		Hamahiga, Okinawa, Japan	2012	TS	26°19'12"N 127°57'59"E	na
KU361938	'Rhipidosiphon' sp.	TS1308	Bryopsidales	Halimedineae	Udoteaceae		Uganzaki, Ishigaki, Japan	2012	TS	24°21'70"N 124°04'51"E	na
KU361939	Chlorodesmis sp.	TS1322	Bryopsidales	Halimedineae	Udoteaceae		Maesato, Ishigaki, Japan	2012	TS	24°19'54"N 124°11'00"E	na
KU361940	'Rhipidosiphon' sp.	TS1324	Bryopsidales	Halimedineae	Udoteaceae		Shiraho, Ishigaki, Japan	2012	TS	24°21'07"N 124°14'40"E	na
KU361941	'Rhipidosiphon' sp.	TS1469	Bryopsidales	Halimedineae	Udoteaceae		Ikema Island, Miyako, Japan	2012	TS	24°55'53"N 125°13'59"E	na
KU361942	'Rhipidosiphon' sp.	TS1476	Bryopsidales	Halimedineae	Udoteaceae		Aragusuku, Miyako, Japan	2012	TS	24°45'37"N 125°25'30"E	na
KU361943	Unres. Udoteaceae	TS1503	Bryopsidales	Halimedineae	Udoteaceae	X	Hamahiga, Okinawa, Japan	2012	TS	26°19'12"N 127°57'59"E	na
KU361944	'Rhipidosiphon' sp.	TS1544	Bryopsidales	Halimedineae	Udoteaceae		Sesoko Island, Okinawa, Japan	2012	TS	26°39'00"N 127°52'25"E	na
KU361945	'Rhipidosiphon' sp.	TS1547	Bryopsidales	Halimedineae	Udoteaceae		Sesoko Island, Okinawa, Japan	2012	TS	26°39'00"N 127°52'25"E	na
KU361946	Rhipocephalus sp.	TS1793	Bryopsidales	Halimedineae	Udoteaceae		BathTub beach, Florida, USA	2013	TS	27°11'06"N 80°09'35"W	na
KU361947	Rhipocephalus sp.	TS1800/TS1802	Bryopsidales	Halimedineae	Udoteaceae		Summerland Key, Florida, USA	2013	TS	24°39'46"N 81°27'51"W	na
KU361948	Rhipocephalus sp.	TS1822	Bryopsidales	Halimedineae	Udoteaceae		Venture Key, Florida, USA	2013	TS	24°38'46"N 81°27'59"W	na
KU361949	'Rhipidosiphon' sp.	TS1901	Bryopsidales	Halimedineae	Udoteaceae		Big Pine Shoals, Florida, USA	2013	TS	--	na

GenBank	Genbank Identifier	Collection #	Order	Suborder	Family	Cult.	Geographical origin	Year	Coll.	GPS coordinates	Habitat
KU361950	'Rhipidosiphon' sp./[Ripocephalus sp.][1]	TS1905	Bryopsidales	Halimedineae	Udoteaceae	X	Garden Key, Dry Tortugas, Florida, USA	2014	TS	24°37'47"N 82°52'17"W	na
KU361951	Rhipiliopsis' sp./[Unres.]	TS1178	Bryopsidales	Halimedineae	Unresolved		Tokashki, Kerama Islands, Okinawa, Japan	2012	TS	26°10'09"N 127°20'33"E	na
KU361952	'Pseudochlorodesmis' sp./[Unres.]	TS1604	Bryopsidales	Halimedineae	Unresolved		Aragusuku, Miyako, Japan	2012	TS	24°45'37"N 125°25'30"E	na
KU361953	Rhipiliopsis' sp./[Unres.]	TS1632	Bryopsidales	Halimedineae	Unresolved		Urazoko, Ishigaki, Japan	2012	TS	24°27'15"N 124°13'14"E	[LPS]
KU361954	Rhipiliopsis' sp./[Unres.]	TS1651	Bryopsidales	Halimedineae	Unresolved		Chatan, Okinawa, Japan	2012	TS	26°19'43"N 127°44'38"E	na
KU361955	'Ostreobium' sp./[Hamid.]	TS0407A	Bryopsidales	Ostreobidineae	'Hamidaceae'	X	Makai Pier, Oahu, Hawaii, USA	2009	TS	21°19'11"N 157°40'10"W	[SPS]
KU361956	'Ostreobium' sp./[Hamid.]	TS0938	Bryopsidales	Ostreobidineae	'Hamidaceae'	X	Sharm el Sheikh (na), Egypt	2011	TS	--	na
KU361957	'Ostreobium' sp./[Hamid.]	TS1385	Bryopsidales	Ostreobidineae	'Hamidaceae'	X	Haemida, Iriomote, Japan	2012	TS	24°16'15"N 123°49'52"E	[LPS]
KU361958	'Ostreobium' sp./[Hamid.]	TS1500	Bryopsidales	Ostreobidineae	'Hamidaceae'		Ueno, Miyako, Japan	2012	TS	24°43'28"N 125°21'33"E	[SPS]
KU361959	'Ostreobium' sp./[Hamid.]	TS1606	Bryopsidales	Ostreobidineae	'Hamidaceae'		Aragusuku, Miyako, Japan	2012	TS	24°45'37"N 125°25'30"E	na
KU361960	'Ostreobium' sp./[Hamid.]	TS1655	Bryopsidales	Ostreobidineae	'Hamidaceae'	X	Commercial Oyster, Atlantic USA	--	MS	--	[Shell]
KU361961	'Ostreobium' sp./[Hamid.]	TS1659	Bryopsidales	Ostreobidineae	'Hamidaceae'		Chatan, Okinawa, Japan		TS	26°19'43"N 127°44'38"E	[Peyss.]
KU361962	'Ostreobium' sp./[Hamid.]	TS1692	Bryopsidales	Ostreobidineae	'Hamidaceae'	X	Jupiter, Florida, USA	2013	TS	26°57'09"N 80°04'41"W	[Shell]
KU361963	'Ostreobium' sp./[Hamid.]	TS1694A	Bryopsidales	Ostreobidineae	'Hamidaceae'	X	Jupiter, Florida, USA	2013	TS	26°57'09"N 80°04'41"W	[Shell]
KU361964	'Ostreobium' sp./[Hamid.]	TS1694B	Bryopsidales	Ostreobidineae	'Hamidaceae'	X	Jupiter, Florida, USA	2013	TS	26°57'09"N 80°04'41"W	[Shell]
KU361965	'Ostreobium' sp./[Hamid.]	TS1695	Bryopsidales	Ostreobidineae	'Hamidaceae'	X	Jupiter, Florida, USA	2013	TS	26°57'09"N 80°04'41"W	[Shell]
KU361966	'Ostreobium' sp./[Hamid.]	TS1846	Bryopsidales	Ostreobidineae	'Hamidaceae'	X	Jupiter, Florida, USA	2013	TS	26°57'08"N 80°04'41"W	[Shell]
KU361967	'Ostreobium' sp./[Hamid.]	TS1876	Bryopsidales	Ostreobidineae	'Hamidaceae'	X	Manatee River, Florida, USA	2013	TS	--	[Shell]
KU361968	'Ostreobium' sp./[Hamid.]	TS1878	Bryopsidales	Ostreobidineae	'Hamidaceae'	X	Venice Jetty, Florida, USA	2013	TS	--	[Shell]
KU361969	'Ostreobium' sp./[Maed.][3]	L30	Bryopsidales	Ostreobidineae	'Maedaceae'		Offshore, Maui, Hawaii, USA	2011	HS	20°46'40"N 156°40'39"W	na
KU361970	'Ostreobium' sp./[Maed.][3]	L36	Bryopsidales	Ostreobidineae	'Maedaceae'		Offshore, Maui, Hawaii, USA	2011	HS	20°46'30"N 156°40'19"W	na
KU361971	'Ostreobium' sp./[Maed.][2]	L64	Bryopsidales	Ostreobidineae	'Maedaceae'		Offshore, Maui, Hawaii, USA	2011	HS	20°48'50"N 156°43'09"W	na
KU361972	'Ostreobium' sp./[Maed.]	TS0711	Bryopsidales	Ostreobidineae	'Maedaceae'	X	IUI beach, Eilat, Israel	2011	TS	29°30'07"N 34°55'05"E	na
KU361973	'Ostreobium' sp./[Maed.]	TS0713	Bryopsidales	Ostreobidineae	'Maedaceae'	X	IUI beach, Eilat, Israel	2011	TS	29°30'07"N 34°55'05"E	na
KU361974	'Ostreobium' sp./[Maed.]	TS0979	Bryopsidales	Ostreobidineae	'Maedaceae'	X	Coral Beach, Eilat, Israel	2011	TS	29°30'13"N 34°55'08"E	na
KU361975	'Ostreobium' sp./[Maed.]	TS0980	Bryopsidales	Ostreobidineae	'Maedaceae'	X	Coral Beach, Eilat, Israel	2011	TS	29°30'13"N 34°55'08"E	na
KU361976	'Ostreobium' sp./[Maed.]	TS0991	Bryopsidales	Ostreobidineae	'Maedaceae'	X	IUI beach, Eilat, Israel	2011	TS	29°30'07"N 34°55'05"E	na
KU361977	'Ostreobium' sp./[Maed.]	TS1101	Bryopsidales	Ostreobidineae	'Maedaceae'	X	--	--	TS	--	na
KU361978	'Ostreobium' sp./[Maed.]	TS1265	Bryopsidales	Ostreobidineae	'Maedaceae'		Maeda, Okinawa, Japan	2012	TS	26°26'35"N 127°46'06"E	[CaCO3]
KU361979	'Ostreobium' sp./[Maed.]	TS1269	Bryopsidales	Ostreobidineae	'Maedaceae'	X	Odo, Okinawa, Japan	2012	TS	26°05'21"N 127°42'38"E	[LPS]
KU361980	'Ostreobium' sp./[Maed.]	TS1274	Bryopsidales	Ostreobidineae	'Maedaceae'	X	Odo, Okinawa, Japan	2012	TS	26°05'21"N 127°42'38"E	[LPS]
KU361981	'Ostreobium' sp./[Maed.]	TS1366	Bryopsidales	Ostreobidineae	'Maedaceae'	X	Unarizaki, Iriomote, Japan	2012	TS	24°25'33"N 123°45'56"E	[LPS]
KU361982	'Ostreobium' sp./[Maed.]	TS1410B	Bryopsidales	Ostreobidineae	'Maedaceae'	X	Sonai, Iriomote, Japan	2012	TS	24°23'20"N 123°44'45"E	na
KU361983	'Ostreobium' sp./[Maed.]	TS1410C	Bryopsidales	Ostreobidineae	'Maedaceae'	X	Sonai, Iriomote, Japan	2012	TS	24°23'20"N 123°44'45"E	na
KU361984	'Ostreobium' sp./[Maed.]	TS1416	Bryopsidales	Ostreobidineae	'Maedaceae'		Shiraho, Ishigaki, Japan	2012	TS	24°21'07"N 124°14'40"E	[SPS]
KU361985	'Ostreobium' sp./[Maed.]	TS1438	Bryopsidales	Ostreobidineae	'Maedaceae'	X	Akashi, Ishigaki, Japan	2012	TS	24°32'15"N 124°18'15"E	[LPS]
KU361986	'Ostreobium' sp./[Maed.]	TS1441	Bryopsidales	Ostreobidineae	'Maedaceae'		Uganzaki, Ishigaki, Japan	2012	TS	24°21'70"N 124°04'51"E	[SPS]
KU361987	'Ostreobium' sp./[Maed.]	TS1506	Bryopsidales	Ostreobidineae	'Maedaceae'		Ueno, Miyako, Japan	2012	TS	24°43'28"N 125°21'33"E	[LPS]
KU361988	'Ostreobium' sp./[Maed.]	TS1573	Bryopsidales	Ostreobidineae	'Maedaceae'		Aragusuku, Miyako, Japan	2012	TS	24°45'37"N 125°25'30"E	na
KU361989	'Ostreobium' sp./[Maed.]	TS1592	Bryopsidales	Ostreobidineae	'Maedaceae'		Aragusuku, Miyako, Japan	2012	TS	24°45'37"N 125°25'30"E	[SPS]
KU361990	'Ostreobium' sp./[Maed.]	TS1597	Bryopsidales	Ostreobidineae	'Maedaceae'		Aragusuku, Miyako, Japan	2012	TS	24°45'37"N 125°25'30"E	[LPS]
KU361991	'Ostreobium' sp./[Maed.]	TS1599	Bryopsidales	Ostreobidineae	'Maedaceae'		Aragusuku, Miyako, Japan	2012	TS	24°45'37"N 125°25'30"E	na
KU361992	'Ostreobium' sp./[Maed.]	TS1614	Bryopsidales	Ostreobidineae	'Maedaceae'		Kurima Island, Miyako, Japan	2012	TS	24°43'03"N 125°14'24"E	[SPS]
KU361993	'Ostreobium' sp./[Maed.]	TS1660	Bryopsidales	Ostreobidineae	'Maedaceae'		Unarizaki, Iriomote, Japan	2012	TS	24°25'33"N 123°45'56"E	[Peyss.]
KU361994	'Ostreobium' sp./[Maed.]	TS1849	Bryopsidales	Ostreobidineae	'Maedaceae'	X	West Palm Beach Inlet, Florida, USA	2013	TS	26°32'39"N 80°02'40"W	[Shell]
KU361995	'Ostreobium' sp./[Maed.][2]	TS1916	Bryopsidales	Ostreobidineae	'Maedaceae'		Sackett Bank, offshore Louisiana, USA	2012	JR	28°38'12"N 89°32'55"W	[Corall.]
KU361996	'Ostreobium' sp./[Maed.][2]	TS1917	Bryopsidales	Ostreobidineae	'Maedaceae'		Sackett Bank, offshore Louisiana, USA	2011	JR	28°38'15"N 89°33'15"W	[Corall.]
KU361997	'Ostreobium' sp./[Odoa.][1]	TS0098	Bryopsidales	Ostreobidineae	'Odoaceae'	X	Offshore, Maui, Hawaii, USA	2006	TS	--	[SPS]
KU361998	'Ostreobium' sp./[Odoa.]	TS0407B	Bryopsidales	Ostreobidineae	'Odoaceae'	X	Makai Pier, Oahu, Hawaii, USA	2009	TS	21°19'11"N 157°40'10"W	[SPS]
KU361999	'Ostreobium' sp./[Odoa.]	TS0408	Bryopsidales	Ostreobidineae	'Odoaceae'	X	Moku-o-loe Island, Oahu, Hawaii, USA	2009	TS	21°25'51"N 157°47'19"W	[SPS]
KU362000	'Ostreobium' sp./[Odoa.]	TS1216	Bryopsidales	Ostreobidineae	'Odoaceae'		Maeda, Okinawa, Japan	2012	TS	26°26'35"N 127°46'06"E	[LPS]
KU362001	'Ostreobium' sp./[Odoa.]	TS1217B	Bryopsidales	Ostreobidineae	'Odoaceae'	X	Maeda, Okinawa, Japan	2012	TS	26°26'35"N 127°46'06"E	[LPS]
KU362002	'Ostreobium' sp./[Odoa.]	TS1267	Bryopsidales	Ostreobidineae	'Odoaceae'	X	Odo, Okinawa, Japan	2012	TS	26°05'21"N 127°42'38"E	[LPS]
KU362003	'Ostreobium' sp./[Odoa.]	TS1273	Bryopsidales	Ostreobidineae	'Odoaceae'	X	Odo, Okinawa, Japan	2012	TS	26°05'21"N 127°42'38"E	[LPS]
KU362004	'Ostreobium' sp./[Odoa.]	TS1275	Bryopsidales	Ostreobidineae	'Odoaceae'	X	Odo, Okinawa, Japan	2012	TS	26°05'21"N 127°42'38"E	[LPS]
KU362005	'Ostreobium' sp./[Odoa.]	TS1281	Bryopsidales	Ostreobidineae	'Odoaceae'		Odo, Okinawa, Japan	2012	TS	26°05'21"N 127°42'38"E	[SPS]
KU362006	'Ostreobium' sp./[Odoa.]	TS1283	Bryopsidales	Ostreobidineae	'Odoaceae'	X	Odo, Okinawa, Japan	2012	TS	26°05'21"N 127°42'38"E	[SPS]
KU362007	'Ostreobium' sp./[Odoa.]	TS1367	Bryopsidales	Ostreobidineae	'Odoaceae'	X	Unarizaki, Iriomote, Japan	2012	TS	24°25'33"N 123°45'56"E	[SPS]

GenBank	Genbank Identifier	Collection #	Order	Suborder	Family	Cult.	Geographical origin	Year	Coll.	GPS coordinates	Habitat
KU362008	'Ostreobium' sp./[Odoa.]	TS1368	Bryopsidales	Ostreobidineae	'Odoaceae'		Unarizaki, Iriomote, Japan	2012	TS	24°25'33"N 123°45'56"E	[LPS]
KU362009	'Ostreobium' sp./[Odoa.]	TS1372	Bryopsidales	Ostreobidineae	'Odoaceae'	X	Unarizaki, Iriomote, Japan	2012	TS	24°25'33"N 123°45'56"E	[SPS]
KU362010	'Ostreobium' sp./[Odoa.]	TS1374	Bryopsidales	Ostreobidineae	'Odoaceae'	X	Unarizaki, Iriomote, Japan	2012	TS	24°25'33"N 123°45'56"E	[SPS]
KU362011	'Ostreobium' sp./[Odoa.]	TS1383	Bryopsidales	Ostreobidineae	'Odoaceae'	X	Haemida, Iriomote, Japan	2012	TS	24°16'15"N 123°49'52"E	[LPS]
KU362012	'Ostreobium' sp./[Odoa.]	TS1387	Bryopsidales	Ostreobidineae	'Odoaceae'		Haemida, Iriomote, Japan	2012	TS	24°16'15"N 123°49'52"E	[LPS]
KU362013	'Ostreobium' sp./[Odoa.]	TS1390	Bryopsidales	Ostreobidineae	'Odoaceae'		Haemida, Iriomote, Japan	2012	TS	24°16'15"N 123°49'52"E	[LPS]
KU362014	'Ostreobium' sp./[Odoa.]	TS1391	Bryopsidales	Ostreobidineae	'Odoaceae'	X	Haemida, Iriomote, Japan	2012	TS	24°16'15"N 123°49'52"E	[LPS]
KU362015	'Ostreobium' sp./[Odoa.]	TS1408	Bryopsidales	Ostreobidineae	'Odoaceae'	X	Sonai, Iriomote, Japan	2012	TS	24°23'20"N 123°44'45"E	[SPS]
KU362016	'Ostreobium' sp./[Odoa.]	TS1415	Bryopsidales	Ostreobidineae	'Odoaceae'		Sonai, Iriomote, Japan	2012	TS	24°23'20"N 123°44'45"E	[SPS]
KU362017	'Ostreobium' sp./[Odoa.]	TS1418	Bryopsidales	Ostreobidineae	'Odoaceae'	X	Shiraho, Ishigaki, Japan	2012	TS	24°21'07"N 124°14'40"E	[LPS]
KU362018	'Ostreobium' sp./[Odoa.]	TS1420A	Bryopsidales	Ostreobidineae	'Odoaceae'		Shiraho, Ishigaki, Japan	2012	TS	24°21'07"N 124°14'40"E	[LPS]
KU362019	'Ostreobium' sp./[Odoa.]	TS1420B	Bryopsidales	Ostreobidineae	'Odoaceae'		Shiraho, Ishigaki, Japan	2012	TS	24°21'07"N 124°14'40"E	[LPS]
KU362020	'Ostreobium' sp./[Odoa.]	TS1421	Bryopsidales	Ostreobidineae	'Odoaceae'		Shiraho, Ishigaki, Japan	2012	TS	24°21'07"N 124°14'40"E	[SPS]
KU362021	'Ostreobium' sp./[Odoa.]	TS1422	Bryopsidales	Ostreobidineae	'Odoaceae'	X	Shiraho, Ishigaki, Japan	2012	TS	24°21'07"N 124°14'40"E	[SPS]
KU362022	'Ostreobium' sp./[Odoa.]	TS1426	Bryopsidales	Ostreobidineae	'Odoaceae'	X	Maesato, Ishigaki, Japan	2012	TS	24°19'54"N 124°11'00"E	[LPS]
KU362023	'Ostreobium' sp./[Odoa.]	TS1427	Bryopsidales	Ostreobidineae	'Odoaceae'	X	Maesato, Ishigaki, Japan	2012	TS	24°19'54"N 124°11'00"E	[LPS]
KU362024	'Ostreobium' sp./[Odoa.]	TS1429	Bryopsidales	Ostreobidineae	'Odoaceae'		Maesato, Ishigaki, Japan	2012	TS	24°19'54"N 124°11'00"E	[LPS]
KU362025	'Ostreobium' sp./[Odoa.]	TS1432	Bryopsidales	Ostreobidineae	'Odoaceae'	X	Maeda, Okinawa, Japan	2012	TS	26°26'35"N 127°46'06"E	[CaCO3]
KU362026	'Ostreobium' sp./[Odoa.]	TS1433	Bryopsidales	Ostreobidineae	'Odoaceae'		Akashi, Ishigaki, Japan	2012	TS	24°32'15"N 124°18'15"E	[SPS]
KU362027	'Ostreobium' sp./[Odoa.]	TS1434	Bryopsidales	Ostreobidineae	'Odoaceae'		Akashi, Ishigaki, Japan	2012	TS	24°32'15"N 124°18'15"E	[SPS]
KU362028	'Ostreobium' sp./[Odoa.]	TS1436	Bryopsidales	Ostreobidineae	'Odoaceae'		Akashi, Ishigaki, Japan	2012	TS	24°32'15"N 124°18'15"E	[SPS]
KU362029	'Ostreobium' sp./[Odoa.]	TS1509	Bryopsidales	Ostreobidineae	'Odoaceae'		Ikema Island, Miyako, Japan	2012	TS	24°55'53"N 125°13'59"E	[LPS]
KU362030	'Ostreobium' sp./[Odoa.]	TS1515	Bryopsidales	Ostreobidineae	'Odoaceae'		Ikema Island, Miyako, Japan	2012	TS	24°55'53"N 125°13'59"E	[SPS]
KU362031	'Ostreobium' sp./[Odoa.]	TS1519	Bryopsidales	Ostreobidineae	'Odoaceae'		Ikema Island, Miyako, Japan	2012	TS	24°55'53"N 125°13'59"E	[LPS]
KU362032	'Ostreobium' sp./[Odoa.]	TS1520	Bryopsidales	Ostreobidineae	'Odoaceae'		Ikema Island, Miyako, Japan	2012	TS	24°55'53"N 125°13'59"E	[LPS]
KU362033	'Ostreobium' sp./[Odoa.]	TS1521	Bryopsidales	Ostreobidineae	'Odoaceae'		Ikema Island, Miyako, Japan	2012	TS	24°55'53"N 125°13'59"E	[LPS]
KU362034	'Ostreobium' sp./[Odoa.]	TS1553B	Bryopsidales	Ostreobidineae	'Odoaceae'		Sesoko Island, Okinawa, Japan	2012	TS	26°39'00"N 127°52'25"E	[SPS]
KU362035	'Ostreobium' sp./[Odoa.]	TS1566	Bryopsidales	Ostreobidineae	'Odoaceae'		Ikema Island, Miyako, Japan	2012	TS	24°55'53"N 125°13'59"E	[SPS]
KU362036	'Ostreobium' sp./[Odoa.]	TS1574A	Bryopsidales	Ostreobidineae	'Odoaceae'		Aragusuku, Miyako, Japan	2012	TS	24°45'37"N 125°25'30"E	[LPS]
KU362037	'Ostreobium' sp./[Odoa.]	TS1574B	Bryopsidales	Ostreobidineae	'Odoaceae'		Aragusuku, Miyako, Japan	2012	TS	24°45'37"N 125°25'30"E	[LPS]
KU362038	'Ostreobium' sp./[Odoa.]	TS1574C	Bryopsidales	Ostreobidineae	'Odoaceae'		Aragusuku, Miyako, Japan	2012	TS	24°45'37"N 125°25'30"E	[LPS]
KU362039	'Ostreobium' sp./[Odoa.]	TS1591S	Bryopsidales	Ostreobidineae	'Odoaceae'	X	Aragusuku, Miyako, Japan	2012	TS	24°45'37"N 125°25'30"E	[SPS]
KU362040	'Ostreobium' sp./[Odoa.]	TS1594	Bryopsidales	Ostreobidineae	'Odoaceae'		Aragusuku, Miyako, Japan	2012	TS	24°45'37"N 125°25'30"E	[SPS]
KU362041	'Ostreobium' sp./[Odoa.]	TS1598	Bryopsidales	Ostreobidineae	'Odoaceae'		Aragusuku, Miyako, Japan	2012	TS	24°45'37"N 125°25'30"E	[LPS]
KU362042	'Ostreobium' sp./[Odoa.]	TS1600	Bryopsidales	Ostreobidineae	'Odoaceae'		Aragusuku, Miyako, Japan	2012	TS	24°45'37"N 125°25'30"E	[LPS]
KU362043	'Ostreobium' sp./[Odoa.]	TS1609	Bryopsidales	Ostreobidineae	'Odoaceae'		Kurima Island, Miyako, Japan	2012	TS	24°43'03"N 125°14'24"E	[LPS]
KU362044	'Ostreobium' sp./[Odoa.]	TS1613	Bryopsidales	Ostreobidineae	'Odoaceae'		Kurima Island, Miyako, Japan	2012	TS	24°43'03"N 125°14'24"E	[LPS]
KU362045	'Ostreobium' sp./[Odoa.]	TS1615	Bryopsidales	Ostreobidineae	'Odoaceae'	X	Kurima Island, Miyako, Japan	2012	TS	24°43'03"N 125°14'24"E	[SPS]
KU362046	'Ostreobium' sp./[Odoa.]	TS1630	Bryopsidales	Ostreobidineae	'Odoaceae'		Urazoko, Ishigaki, Japan	2012	TS	24°27'15"N 124°13'14"E	[LPS]
KU362047	'Ostreobium' sp./[Odoa.]	TS1633	Bryopsidales	Ostreobidineae	'Odoaceae'		Urazoko, Ishigaki, Japan	2012	TS	24°27'15"N 124°13'14"E	[LPS]
KU362048	'Ostreobium' sp./[Odoa.]	TS1634	Bryopsidales	Ostreobidineae	'Odoaceae'		Urazoko, Ishigaki, Japan	2012	TS	24°27'15"N 124°13'14"E	[LPS]
KU362049	'Ostreobium' sp./[Odoa.]	TS1635	Bryopsidales	Ostreobidineae	'Odoaceae'		Urazoko, Ishigaki, Japan	2012	TS	24°27'15"N 124°13'14"E	[LPS]
KU362050	'Ostreobium' sp./[Unar.]	ARS0603	Bryopsidales	Ostreobidineae	'Unarizakiaceae'	X	Moku-o-loe Island, Oahu, Hawaii, USA	2005	TS	21°25'51"N 157°47'19"W	[SPS]
KU362051	'Ostreobium' sp./[Unar.]	TS1369	Bryopsidales	Ostreobidineae	'Unarizakiaceae'	X	Unarizaki, Iriomote, Japan	2012	TS	24°25'33"N 123°45'56"E	[LPS]
KU362052	'Ostreobium' sp./[Unar.]	TS1602	Bryopsidales	Ostreobidineae	'Unarizakiaceae'		Aragusuku, Miyako, Japan	2012	TS	24°45'37"N 125°25'30"E	[LPS]
KU362053	Dasycladus vermicularis	TS1094	Dasycladales	--	Dasycladaceae		Hosh Hanikra South, Israel	2011	TS	33°02'33"N 35°05'57"E	na
KU362054	Batophora sp.	TS1812	Dasycladales	--	Dasycladaceae		Summerland Key, Florida, USA	2013	TS	24°39'46"N 81°27'51"W	na
KU362055	Parvocaulis parvulus	TS0026/ARS1083	Dasycladales	--	Polyphysaceae		Kahala, Oahu, Hawaii, USA	2006	NP	21°15'35"N 157°47'14"W	na
KU362056	Acetabularia sp.	TS1210	Dasycladales	--	Polyphysaceae		Hamahiga, Okinawa, Japan	2012	TS	26°19'11"N 127°58'00"E	na
KU362057	Parvocaulis parvulus	TS1590	Dasycladales	--	Polyphysaceae		Hamahiga,Okinawa, Japan	2012	TS	26°19'12"N 127°57'59"E	na
KU362058	Parvocaulis parvulus	TS1702	Dasycladales	--	Polyphysaceae		Jupiter, Florida, USA	2013	TS	26°57'09"N 80°04'41"W	na
KU362059	Acetabularia sp.	TS1704	Dasycladales	--	Polyphysaceae		Jupiter, Florida, USA	2013	TS	26°57'09"N 80°04'41"W	na
KU362060	Acetabularia sp.	TS1811	Dasycladales	--	Polyphysaceae		Summerland Key, Florida, USA	2013	TS	24°39'46"N 81°27'51"W	na
KU362061	Acetabularia sp.	TS1860	Dasycladales	--	Polyphysaceae		Howard Park, Tarpon Springs, Florida, USA	2013	TS	28°09'14"N 82°48'24"W	na
KU362062	Phaeophila sp.	TS1583	'Ulvales-Ulothrichales'	--	Phaeophilaceae	X	Sonai, Iriomote, Japan	2012	TS	24°23'20"N 123°44'45"E	[CaCO3]
KU362063	Ulva sp.	TS1729	'Ulvales-Ulothrichales'	--	Ulvaceae		Walton Rocks Beach, Florida, USA	2013	TS	27°20'17"N 80°13'59"W	na
KU362064	Ulva sp.[1]	TS1919	'Ulvales-Ulothrichales'	--	Ulvaceae		Aquarium, Offshore Dry Tortugas, USA	2012	TS	--	na
KU362065	Unres. Ulvellaceae[1]	TS0409	'Ulvales-Ulothrichales'	--	Ulvellaceae	X	Off Ala Moana Beach Park, Oahu, Hawaii, USA	2009	DP	21°16'54"N 157°51'15"W	[Avrainvillea]

GenBank	Genbank Identifier	Collection #	Order	Suborder	Family	Cult.	Geographical origin	Year	Coll.	GPS coordinates	Habitat
KU362066	Unres. Ulvellaceae	TS0410	'Ulvales-Ulothrichales'	--	Ulvellaceae	X	Moku-o-loe Island, Oahu, Hawaii, USA	2009	TS	21°25'51"N 157°47'19"W	na
KU362067	Unres. Ulvellaceae	TS1258	'Ulvales-Ulothrichales'	--	Ulvellaceae	X	Hamahiga, Okinawa, Japan	2012	TS	26°19'12"N 127°57'59"E	na
KU362068	Unres. Ulvellaceae	TS1522	'Ulvales-Ulothrichales'	--	Ulvellaceae	X	Akashi, Ishigaki, Japan	2012	TS	24°32'15"N 124°18'15"E	[SPS]
KU362069	Unres. Ulvellaceae	TS1548	'Ulvales-Ulothrichales'	--	Ulvellaceae	X	Akashi, Ishigaki, Japan	2012	TS	24°32'15"N 124°18'15"E	[SPS]
KU362070	Unres. Ulvellaceae	TS1637	'Ulvales-Ulothrichales'	--	Ulvellaceae	X	Akashi, Ishigaki, Japan	2012	TS	24°32'15"N 124°18'15"E	[SPS]
KU362071	Unres. Ulvellaceae	TS1848	'Ulvales-Ulothrichales'	--	Ulvellaceae	X	Jupiter, Florida, USA	2013	TS	26°57'08"N 80°04'41"W	na

[1] ~40 m depth range
[2] ~60 m depth range
[3] >100 m depth range
