# Supplementary material for: A metabarcoding framework for facilitated survey of endolithic phototrophs with tufA
Source: BMC Ecol. 2016 Mar 10;16:8. doi: 10.1186/s12898-016-0068-x (PMC4785743; doi:10.1186/s12898-016-0068-x)
Supplement: Supplementary file 2 — 10.1186/s12898-016-0068-x Barcoded Florideophyceae, Phaeophyceae and miscellaneous taxa. Collection information for specimens of the Florideophyceae and Phaeophyceae sequenced for tufA in the present study (as well as a miscellaneous Prasinophyceae). Polyphyletic orders or family and combined orders are indicated in between quotes. All specimens from shallow waters (0–10 m) otherwise indicated in footnotes. Collector (Coll.) initials as follows: CP = C. Pueschel, CS = C. Stoude, DG = D. Gabriel, DK = D. Krayesky, DWF = D.W. Freshwater, EC = E. Coppejans, ED = E. Deslandes, JC = J. Cabioch, JH = J. Hughey, JR = J. Richards, JRu = J. Rueness, JZ = J. Zertuche, MG = M. Guiry, MHH = M. H. Hommersand, MJW = M. J. Wynne, MY = M. Yoshizaki, OC = O. Camacho, SF = S. Fredericq. [file 12898_2016_68_MOESM2_ESM.pdf]

GenBank	Genbank Identifier	Collection #	Class	Order	Family	Geographical origin	Year	Collector	GPS coordinates
KU362073	Batrachospermum sp.	LAF7127	Florideophyceae	Batrachospermales	Batrachospermaceae	North Carolina, USA	1993	DWF	--
KU362074	Paralemanea annulata	LAF7143	Florideophyceae	Batrachospermales	Lemaneaceae	Orange County, North Carolina, USA	1993	MHH	--
KU362075	Bonnemaisonia asparagoides	LAF7129	Florideophyceae	Bonnemaisoniales	Bonnemaisoniaceae	Norway	--	JRu	--
KU362076	Acanthophora sp.	TS0788	Florideophyceae	Ceramiales	Rhodomelaceae	Suez, Egypt	2011	TS	29°57'07"N 32°33'51"E
KU362077	Spyridia hypnoides	TC2794	Florideophyceae	Ceramiales	Spyridiaceae	Reunion Rocks, Kwa-Zulu Natal, South Africa	2005	SF	29°58'56"S 30°57'55"E
KU362078	Spyridia cupressina	TC2740	Florideophyceae	Ceramiales	Spyridiaceae	Umdloti Beach, Kwa-Zulu Natal, South Africa	2005	SF	29°40'05"S 31°07'01"E
KU362079	Porolithon sp.[1]	LAF6641	Florideophyceae	'Corallinales-Hapalidiales'	Corallinaceae	Ewing Bank, offshore Louisiana, USA	2012	JR	28°05'50"N 91°01'37"W
KU362080	Neogoniolithon sp.	TS1944	Florideophyceae	'Corallinales-Hapalidiales'	Corallinaceae	Florida Keys, Florida, USA	2014	TS	24°38'13"N 81°23'39"W
KU362081	Litophyllum sp.	LAF4294	Florideophyceae	'Corallinales-Hapalidiales'	Corallinaceae	Sackett Bank, offshore Louisiana, USA	2011	JR	28°38'15"N 89°33'15"W
KU362082	Amphiroa sp.	LAF7125	Florideophyceae	'Corallinales-Hapalidiales'	Corallinaceae	South Africa	--	--	--
KU362083	Mesophyllum sp.	LAF6734	Florideophyceae	'Corallinales-Hapalidiales'	Hapalidiaceae	Aquarium, Ewing Bank, offshore Louisiana, USA	2012	SF	--
KU362084	Capreolia implexa	LAF7130	Florideophyceae	Gelidiales	Gelidiaceae	Australia	--	MG	--
KU362085	Gelidium sp.	LAF7136	Florideophyceae	Gelidiales	Gelidiaceae	Galveston, Texas, USA	2003	DK	--
KU362086	Ptilophora subcostata	LAF7128	Florideophyceae	Gelidiales	Gelidiaceae	Japan	--	DWF	--
KU362087	Unknown Gelidiaceae	LAF7149	Florideophyceae	Gelidiales	Gelidiaceae	Swakopmund, Namibia	1993	MHH	22°39'59"S 14°32'04"E
KU362088	Acrochaetium sagraeanum	LAF7126	Florideophyceae	Acrochaetiales	Acrochaetiaceae	--	--	--	--
KU362089	Callophycus oppositifolius	LAF0551	Florideophyceae	'Gigartinales' s. stricto	Areschougiaceae	Cervantes, Australia	1995	MHH	30°30'08"S 115°04'32"E
KU362090	Erythroclonium muelleri	LAF0455	Florideophyceae	'Gigartinales' s. stricto	Areschougiaceae	Kangaroo Island, Australia	1995	MHH	35°46'34"S 137°12'56"E
KU362091	Erythroclonium muelleri	LAF0474	Florideophyceae	'Gigartinales' s. stricto	Areschougiaceae	Kangaroo Island, Australia	1995	MHH	35°46'34"S 137°12'56"E
KU362092	Rhabdonia verticillata	LAF0485	Florideophyceae	'Gigartinales' s. stricto	Areschougiaceae	Kangaroo Island, Australia	1995	MHH	35°46'34"S 137°12'56"E
KU362093	Rhabdonia coccinea	LAF0497	Florideophyceae	'Gigartinales' s. stricto	Areschougiaceae	Kangaroo Island, Australia	1995	MHH	35°46'34"S 137°12'56"E
KU362094	Rhabdonia clavigera	LAF0536	Florideophyceae	'Gigartinales' s. stricto	Areschougiaceae	Kangaroo Island, Australia	1995	MHH	35°46'34"S 137°12'56"E
KU362095	Callophycus africanus	LAF0528	Florideophyceae	'Gigartinales' s. stricto	Areschougiaceae	Palm Beach, Kwa-Zulu Natal, South Africa	1993	MHH	30°58'52"S 30°16'23"E
KU362096	Callophycus laxua	LAF0537	Florideophyceae	'Gigartinales' s. stricto	Areschougiaceae	Warrnambool, Australia	1995	MHH	38°22'58"S 142°29'07"E
KU362097	Schmitzia' sp.	LAF7026	Florideophyceae	'Gigartinales' s. Lato	Calosiphoniaceae	Aquarium, Offshore Dry Tortugas, USA	2012	SF	--
KU362098	Dudresnaya crassa	LAF7131	Florideophyceae	'Gigartinales' s. lato	Dumontiaceae	Colombia	1997	--	--
KU362099	Gibsmithia sp.	DG0534	Florideophyceae	'Gigartinales' s. lato	Dumontiaceae	Guam, USA	--	TSc	--
KU362100	Gibsmithia sp.	LAF6136/DG488	Florideophyceae	'Gigartinales' s. lato	Dumontiaceae	Hurghada, Egypt	2012	DG	27°19'33"N 33°47'44"E
KU362101	Dumontia incrassata	K0258	Florideophyceae	'Gigartinales' s. lato	Dumontiaceae	Massachusetts, USA	1993	MHH	--
KU362102	Dilsea californica	K0257	Florideophyceae	'Gigartinales' s. lato	Dumontiaceae	Pigeon Point, California	1992	MHH	37°10'58"N 122°23'34"W
KU362103	Endocladia muricata	LAF7132	Florideophyceae	'Gigartinales' s. lato	Endocladiaceae	Bodega Head, California, USA	1992	MHH	38°19'02"N 123°03'49"W
KU362104	Endocladia muricata	LAF7133	Florideophyceae	'Gigartinales' s. lato	Endocladiaceae	Bodega Head, California, USA	1992	MHH	38°19'02"N 123°03'49"W
KU362105	Gloiopeltis furcata	LAF7137	Florideophyceae	'Gigartinales' s. lato	Endocladiaceae	Botany Bay, Vancouver I., British Columbia, Canada	1995	MJW	--
KU362106	Gloiopeltis furcata	LAF7138	Florideophyceae	'Gigartinales' s. lato	Endocladiaceae	Chiba, Japan	1993	MY	--
KU362107	Halarachnion ligulatum	LAF7140	Florideophyceae	'Gigartinales' s. stricto	Furcellariaceae	Roscoff, France	1996	JC	--
KU362108	Callophyllis pinnata	K0255	Florideophyceae	'Gigartinales' s. lato	Kallymeniaceae	Coquimbo, Chile	1995	SF	29°58'03"S 71°20'09"W
KU362109	Kallymenia cribrosa	K0254	Florideophyceae	'Gigartinales' s. lato	Kallymeniaceae	Tarcoola Beach, Australia	1995	MHH	28°48'31"S 114°37'11"E
KU362110	Portieria hornemannii	LAF7145	Florideophyceae	'Gigartinales' s. lato	Rhizophyllidaceae	Palm Beach, Kwa-Zulu Natal, South Africa	1993	MHH	30°58'52"S 30°16'23"E
KU362111	Rhizophyllis sp.	K0182	Florideophyceae	'Gigartinales' s. lato	Rhizophyllidaceae	Western Florida Shelf, USA	2006	SF	28°50'44"N 85°02'06"W
KU362112	Rhizophyllis sp.	K0184	Florideophyceae	'Gigartinales' s. lato	Rhizophyllidaceae	Western Florida Shelf, USA	2006	SF	28°10'16"N 84°01'57"W
KU362113	Tacanoosca uncinatum	LAF0481	Florideophyceae	'Gigartinales' s. stricto	Solieriaceae	Bahia de Los Angeles, Baja California, Mexico	1995	JZ	28°57'10"N 113°33'41"W
KU362114	Sarcodiotheca gaudichaudii	LAF0482	Florideophyceae	'Gigartinales' s. stricto	Solieriaceae	Coquimbo, Chile	1995	SF	29°58'03"S 71°20'09"W
KU362115	Sarcodiotheca furcata	LAF0448	Florideophyceae	'Gigartinales' s. stricto	Solieriaceae	Lopez I, Washington, USA	1993	CS	48°28'59"N 122°53'45"W
KU362116	Solieria chordalis	LAF0451	Florideophyceae	'Gigartinales' s. stricto	Solieriaceae	Rade de Brest, France	1995	ED	-- '-
KU362117	Eucheuma isiforme	LAF0446	Florideophyceae	'Gigartinales' s. stricto	Solieriaceae	Spanish Harbor Key, Florida, USA	1994	DWF	24°39'08"N 81°18'19"W
KU362118	Meristhotheca papulosa	LAF0450	Florideophyceae	'Gigartinales' s. stricto	Solieriaceae	Tateyana, Chiba, Japan	1993	SF	34°57'42"N 139°51'37"E
KU362119	Gracilaria sp.	LAF7139	Florideophyceae	Gracilariales	Gracilariaceae	Gulf of Caifornia, Mexico	1996	JH	--
KU362120	Hildenbrandia lecanellieri	LAF7141	Florideophyceae	Hildenbrandiales	Hildenbrandiaceae	Buffles Bay, Cape Peninsula, South Africa	1993	MHH	34°19'04"S 18°27'39"E
KU362121	Hildenbrandia rubra	LAF7142	Florideophyceae	Hildenbrandiales	Hildenbrandiaceae	Roscoff, France	1993	MHH	--
KU362122	Galaxaura sp.	LAF7134	Florideophyceae	Nemaliales	Galaxauraceae	Guadeloupe, France	1993	SF	--
KU362123	Galaxaura marginata	LAF7135	Florideophyceae	Nemaliales	Galaxauraceae	Taiwan	1993	SF	--
KU362124	Scinaia sp.	LAF7148	Florideophyceae	Nemaliales	Scinaiaceae	Florida Keys, Florida, USA	--	--	--

GenBank	Genbank Identifier	Collection #	Class	Order	Family	Geographical origin	Year	Collector	GPS coordinates
KU362125	Nothogenia fastigiata	LAF7144	Florideophyceae	Nemaliales	Scinaiaceae	Isla Negra, Prov. San Antonio, Chile	1994	SF	33°26'31"S 71°40'58"W
KU362126	Predaea sp.	LAF7027	Florideophyceae	Nemastomatales	Nemastomataceae	Aquarium, Offshore Dry Tortugas, USA	2011	SF	--
KU362127	Peyssonnelia sp.	LAF5451	Florideophyceae	Peyssonneliales	Peyssonneliaceae	Azuero Peninsula, Veraguas, (Pacific) Panama	2012	WES	--
KU362128	'Cruoriopsis' sp.	LAF6361	Florideophyceae	Peyssonneliales	Peyssonneliaceae	Chatan, Okinawa, Japan	2012	TS	26°19'43"N 127°44'38"E
KU362129	Peyssonnelia sp.	LAF6364	Florideophyceae	Peyssonneliales	Peyssonneliaceae	Chatan, Okinawa, Japan	2012	TS	26°19'43"N 127°44'38"E
KU362130	Peyssonnelia sp.	LAF6386	Florideophyceae	Peyssonneliales	Peyssonneliaceae	Haemida, Iriomote, Japan	2012	TS	24°16'15"N 123°49'52"E
KU362131	Peyssonnelia sp.	K0201	Florideophyceae	Peyssonneliales	Peyssonneliaceae	Offshore Louisiana, USA	2007	SF	27°57'03"N 92°01'49"W
KU362132	Peyssonnelia sp.	LAF4419	Florideophyceae	Peyssonneliales	Peyssonneliaceae	San San Beach, Okinawa, Japan	2011	TSc	26°10'41"N 127°49'44"E
KU362133	Peyssonnelia sp.	LAF6369	Florideophyceae	Peyssonneliales	Peyssonneliaceae	Sesoko Island, Okinawa, Japan	2012	TS	26°39'00"N 127°52'25"E
KU362134	Peyssonnelia sp.	LAF4376/TS0987	Florideophyceae	Peyssonneliales	Peyssonneliaceae	Yam Surf Beach, Israel	2011	TS	29°30'42"N 34°55'28"E
KU362135	Renouxia antillana	LAF6170	Florideophyceae	Rhodogorgonales	Rhodogorgonaceae	Hurghada, Egypt	2012	DG	27°18'39"N 33°47'57"E
KU362136	Rhodogorgon carriebowensis	LAF7147	Florideophyceae	Rhodogorgonales	Rhodogorgonaceae	Papua New Guinea	--	EC	--
KU362137	Renouxia antillana	LAF7146	Florideophyceae	Rhodogorgonales	Rhodogorgonaceae	St. Ann's Bay, Jamaica	1993	CP	18°26'18"N 77°12'05"W
KU362138	Chrysymenia pseudoventricosa[1]	WES0016	Florideophyceae	Rhodymeniales	Rhodymeniaceae	Campeche Banks, Mexico	2005	SF	22°10'42"N 91°09'55"W
KU362139	Chrysymenia nodulosa	WES0096	Florideophyceae	Rhodymeniales	Rhodymeniaceae	Isla de Culebra, Puerto Rico	1995	SF	18°20'26"N 65°20'53"W
KU362140	Chrysymenia nodulosa	WES0148	Florideophyceae	Rhodymeniales	Rhodymeniaceae	Isla de Culebra, Puerto Rico	1995	SF	18°20'26"N 65°20'53"W
KU362141	Leptosomia rosea	WES0112	Florideophyceae	Rhodymeniales	Rhodymeniaceae	Kangaroo Island, Australia	1995	MHH	35°46'34"S 137°12'56"E
KU362142	Sporolithon sp.	LAF5767	Florideophyceae	Sporolithales	Sporolithaceae	El Tor, Egypt	2012	WES	28°28'39"N 34°30'41"E
KU362143	Sporolithon ptychoides	LAF5846	Florideophyceae	Sporolithales	Sporolithaceae	El Tor, Egypt	2012	WES	28°14'04"N 33°36'10"E
KU362144	Sporolithon molle	LAF5848	Florideophyceae	Sporolithales	Sporolithaceae	El Tor, Egypt	2012	WES	28°14'04"N 33°36'10"E
KU362145	Uncultured Phaeophyceae[1]	TS1946	Phaeophyceae	--	--	Florida Middle Grounds, Florida, USA	2006	--	28°30'50"N 84°28'18"W
KU362146	Uncultured Phaeophyceae	TS1945	Phaeophyceae	--	--	Peru	--	--	--
KU362147	Lobophora sp.	LAF6459	Phaeophyceae	Dictyotales	Dictyotaceae	Aquarium, Ewing Bank, offshore Louisiana, USA	2012	TS	--
KU362148	Lobophora sp.	LAF5700	Phaeophyceae	Dictyotales	Dictyotaceae	Dahab, Egypt	2012	TS	28°28'39"N 34°30'13"E
KU362149	Sargassum sp.	TS0847	Phaeophyceae	Fucales	Sargassaceae	El Kheima (inside bay), Egypt	2011	TS	27°51'34"N 34°17'24"E
KU362150	Sargassum sp.	TS0873	Phaeophyceae	Fucales	Sargassaceae	Tiran Island, Egypt	2011	TS	27°59'42"N 34°29'44"E
KU362072	Verdigellas peltata	TS1941	Prasinophyceae	Palmophyllales	Palmophyllaceae	Offshore Louisiana, USA	2011	TS	--
