# Supplementary material for: A metabarcoding framework for facilitated survey of endolithic phototrophs with tufA
Source: BMC Ecol. 2016 Mar 10;16:8. doi: 10.1186/s12898-016-0068-x (PMC4785743; doi:10.1186/s12898-016-0068-x)
Supplement: Supplementary file 3 — 10.1186/s12898-016-0068-x Indexed forward primers used for metabarcoding. Indexes in bold. [file 12898_2016_68_MOESM3_ESM.pdf]

Sample	Indexed env_*tufA* F primer
**FL01**	5'- **CGTGGCTT**TGGGTDGAHAADATTTWYNMNYTRATGR-3'
**FL02**	5'- **CGTGCCAG**TGGGTDGAHAADATTTWYNMNYTRATGR-3'
**JP07**	5'- **CGTCTTTG**TGGGTDGAHAADATTTWYNMNYTRATGR-3'
**GM14**	5'- **CGTCTGTA**TGGGTDGAHAADATTTWYNMNYTRATGR-3'
