# Supplementary material for: A metabarcoding framework for facilitated survey of endolithic phototrophs with tufA
Source: BMC Ecol. 2016 Mar 10;16:8. doi: 10.1186/s12898-016-0068-x (PMC4785743; doi:10.1186/s12898-016-0068-x)
Supplement: Supplementary file 4 — 10.1186/s12898-016-0068-x Summary taxonomy and content of the non-redundant tufA database. Diversity and sequence counts for (A) Heterotrophs and Phototrophs, (B) Phyla and Classes for all phototrophs and (C) Orders/suborders and families for the Ulvophyceae (Genus taxonomy not shown for conciseness). Taxonomic groupings with unsettled naming such as combined orders, provisional families, and polyphyletic families (i.e. ‘Kornamanniaceae’ and ‘Ulothrichaceae’) are noted between quotes. [file 12898_2016_68_MOESM4_ESM.pdf]

A

Category		
**'Heterotrophs'**		2718
**'Phototrophs'**		1339
**Database**	**Σ**	**4057**

B

Phylum	Class	
**Charophyta**	Chlorokybophyceae	**2**
**'Chlorarachniophyta'**	'Chlorarachniophyceae'	**3**
**Chlorophyta**	Chlorophyceae	74
	Pedinophyceae	5
	Prasinophyceae	19
	Trebouxiophyceae	68
	Ulvophyceae	689
	**Σ**	855
**Cryptophyta**	Cryptophyceae	**12**
**Cyanophyta**	Cyanophyceae	**128**
**Euglenozoa**	Euglenophyceae	**36**
**Glaucophyta**	Glaucophyceae	**3**
**Haptophyta**	Coccolithophyceae	62
	Pavlovophyceae	5
	**Σ**	**67**
**Ochrophyta**	'Bacillariophyta'	29
	Chrysophyceae	1
	Eustigmatophyceae	11
	Pelagophyceae	5
	Phaeophyceae	22
	Raphidophyceae	4
	Xanthophyceae	3
	**Σ**	**75**
**Rhodophyta**	Bangiophyceae	12
	Compsopogonophyceae	3
	Cyanidiophyceae	9
	Florideophyceae	105
	Porphyridiophyceae	4
	Rhodellophyceae	2
	Stylonematophyceae	22
	Unres. Rhodophyta	1
	**Σ**	**158**
	**'Phototrophs' Σ**	**1339**

C

Order/Suborder	Family	
**Bryopsidales/Bryopsidineae**	Bryopsidaceae	34
	Codiaceae	28
	Derbesiaceae	15
	Unres. Bryopsidineae	9
	**Σ**	**86**
**Bryopsidales/Halimedineae**	Caulerpaceae	172
	Dichotomosiphonaceae	5
	Halimedaceae	112
	'Pseudochlorodesmidaceae'	9
	Pseudocodiaceae	8
	'Pseudostreobiaceae'	12
	Rhipiliaceae	19
	'Siphonogramenaceae'	9
	Udoteaceae	28
	Unre. Halimedianeae	3
	**Σ**	**377**
**Bryopsidales/Ostreobidineae**	'Hamidaceae'	11
	'Maedaceae'	22
	'Odoaceae'	11
	'Unarizakiaceae'	3
	**Σ**	**47**
**Dasycladales**	Dasycladaceae	2
	Polyphysaceae	8
	**Σ**	**10**
**Oltmannsiellopsidales**	Oltmannsiellopsidaceae	**1**
**'Ulvales-Ulothrichales'**	Bolbocolaceae	5
	Cloniophoraceae	1
	Gayraliaceae	1
	Halochlorococcumaceae	1
	'Kornmanniaceae'	8
	Monostromataceae	2
	Phaeophilaceae	15
	'Ulothrichaceae'	12
	Ulvaceae	60
	Ulvellaceae	58
	Unres. Ulothrichales-Ulvales	5
	**Σ**	**168**
	**Ulvophyceae Σ**	**689**
