# Supplementary material for: A metabarcoding framework for facilitated survey of endolithic phototrophs with tufA
Source: BMC Ecol. 2016 Mar 10;16:8. doi: 10.1186/s12898-016-0068-x (PMC4785743; doi:10.1186/s12898-016-0068-x)
Supplement: Supplementary file 5 — 10.1186/s12898-016-0068-x Clone taxonomy. Detailed reference taxonomy for 86 phototrophic clones from microfloras sampled in the Ryukyu archipelago (JP01, JP03, JP04, JP06, JP07, JP25), an aquarium window scrap (E09) and a Gulf of Mexico crustose coralline Lithophyllum sp. rhodolith. Taxonomic groupings with unsettled naming such as combined orders, provisional families, and polyphyletic or monophyletic species or genera complexes, are noted between quotes. [file 12898_2016_68_MOESM5_ESM.pdf]

Genbank	Genbank Identifier	Domain	Phyla	Class	Order	Family (Suborder)	Genus
KU362151	Uncultured environmental Phaeophilaceae clone JP1_C	Eukaryota	Chlorophyta	Ulvophyceae	'Ulvales-Ulothrichales'	Phaeophilaceae	Phaeophila
KU362152	Uncultured environmental Phaeophilaceae clone JP1_D	Eukaryota	Chlorophyta	Ulvophyceae	'Ulvales-Ulothrichales'	Phaeophilaceae	Phaeophila
KU362153	Uncultured environmental Phaeophilaceae clone JP1_E	Eukaryota	Chlorophyta	Ulvophyceae	'Ulvales-Ulothrichales'	Phaeophilaceae	Phaeophila
KU362154	Uncultured environmental Phaeophilaceae clone JP1_F	Eukaryota	Chlorophyta	Ulvophyceae	'Ulvales-Ulothrichales'	Phaeophilaceae	Phaeophila
KU362155	Uncultured environmental Phaeophilaceae clone JP1_G	Eukaryota	Chlorophyta	Ulvophyceae	'Ulvales-Ulothrichales'	Phaeophilaceae	Phaeophila
KU362156	Uncultured environmental Phaeophilaceae clone JP1_H	Eukaryota	Chlorophyta	Ulvophyceae	'Ulvales-Ulothrichales'	Phaeophilaceae	Phaeophila
KU362157	Uncultured environmental Phaeophilaceae clone JP25_B	Eukaryota	Chlorophyta	Ulvophyceae	'Ulvales-Ulothrichales'	Phaeophilaceae	Phaeophila
KU362158	Uncultured environmental Phaeophilaceae clone JP25_C	Eukaryota	Chlorophyta	Ulvophyceae	'Ulvales-Ulothrichales'	Phaeophilaceae	Phaeophila
KU362159	Uncultured environmental Phaeophilaceae clone JP3_I	Eukaryota	Chlorophyta	Ulvophyceae	'Ulvales-Ulothrichales'	Phaeophilaceae	Phaeophila
KU362160	Uncultured environmental Phaeophilaceae clone JP6_D	Eukaryota	Chlorophyta	Ulvophyceae	'Ulvales-Ulothrichales'	Phaeophilaceae	Phaeophila
KU362161	Uncultured environmental Ulvellaceae clone E09_A	Eukaryota	Chlorophyta	Ulvophyceae	'Ulvales-Ulothrichales'	Ulvellaceae	Ulvella
KU362162	Uncultured environmental 'Ostreobium' clone JP6_B	Eukaryota	Chlorophyta	Ulvophyceae	Bryopsidales	'Hamidaceae' (Ostreobidineae)	'Ostreobium'
KU362163	Uncultured environmental 'Ostreobium' clone JP7_G	Eukaryota	Chlorophyta	Ulvophyceae	Bryopsidales	'Hamidaceae' (Ostreobidineae)	'Ostreobium'
KU362164	Uncultured environmental 'Ostreobium' clone JP3_G	Eukaryota	Chlorophyta	Ulvophyceae	Bryopsidales	'Maedaceae' (Ostreobidineae)	'Ostreobium'
KU362165	Uncultured environmental 'Ostreobium' clone JP3_H	Eukaryota	Chlorophyta	Ulvophyceae	Bryopsidales	'Maedaceae' (Ostreobidineae)	'Ostreobium'
KU362166	Uncultured environmental 'Ostreobium' clone JP6_A	Eukaryota	Chlorophyta	Ulvophyceae	Bryopsidales	'Maedaceae' (Ostreobidineae)	'Ostreobium'
KU362167	Uncultured environmental 'Ostreobium' clone JP7_D	Eukaryota	Chlorophyta	Ulvophyceae	Bryopsidales	'Maedaceae' (Ostreobidineae)	'Ostreobium'
KU362168	Uncultured environmental 'Ostreobium' clone JP7_E	Eukaryota	Chlorophyta	Ulvophyceae	Bryopsidales	'Maedaceae' (Ostreobidineae)	'Ostreobium'
KU362169	Uncultured environmental 'Ostreobium' clone JP7_F	Eukaryota	Chlorophyta	Ulvophyceae	Bryopsidales	'Maedaceae' (Ostreobidineae)	'Ostreobium'
KU362170	Uncultured environmental 'Ostreobium' clone JP6_C	Eukaryota	Chlorophyta	Ulvophyceae	Bryopsidales	'Odoaceae' (Ostreobidineae)	'Ostreobium'
KU362171	Uncultured environmental 'Ostreobium' clone JP7_H	Eukaryota	Chlorophyta	Ulvophyceae	Bryopsidales	'Odoaceae' (Ostreobidineae)	'Ostreobium'
KU362172	Uncultured environmental 'Ostreobium' clone JP7_I	Eukaryota	Chlorophyta	Ulvophyceae	Bryopsidales	'Odoaceae' (Ostreobidineae)	'Ostreobium'
KU362173	Uncultured environmental 'Ostreobium' clone JP1_A	Eukaryota	Chlorophyta	Ulvophyceae	Bryopsidales	'Pseudostreobiaceae' (Halimedineae)	'Ostreobium'
KU362174	Uncultured environmental 'Ostreobium' clone JP25_A	Eukaryota	Chlorophyta	Ulvophyceae	Bryopsidales	'Pseudostreobiaceae' (Halimedineae)	'Ostreobium'
KU362175	Uncultured environmental 'Ostreobium' clone JP3_A	Eukaryota	Chlorophyta	Ulvophyceae	Bryopsidales	'Pseudostreobiaceae' (Halimedineae)	'Ostreobium'
KU362176	Uncultured environmental 'Ostreobium' clone JP3_B	Eukaryota	Chlorophyta	Ulvophyceae	Bryopsidales	'Pseudostreobiaceae' (Halimedineae)	'Ostreobium'
KU362177	Uncultured environmental 'Ostreobium' clone JP3_C	Eukaryota	Chlorophyta	Ulvophyceae	Bryopsidales	'Pseudostreobiaceae' (Halimedineae)	'Ostreobium'
KU362178	Uncultured environmental 'Ostreobium' clone JP7_A	Eukaryota	Chlorophyta	Ulvophyceae	Bryopsidales	'Pseudostreobiaceae' (Halimedineae)	'Ostreobium'
KU362179	Uncultured environmental 'Ostreobium' clone JP7_B	Eukaryota	Chlorophyta	Ulvophyceae	Bryopsidales	'Pseudostreobiaceae' (Halimedineae)	'Ostreobium'
KU362180	Uncultured environmental 'Pseudochlorodesmis' clone JP3_Q	Eukaryota	Chlorophyta	Ulvophyceae	Bryopsidales	'Pseudochlorodesmidaceae' (Halimedineae)	'Pseudochlorodesmis'
KU362181	Uncultured environmental 'Pseudochlorodesmis' clone S15_A	Eukaryota	Chlorophyta	Ulvophyceae	Bryopsidales	'Siphonogramenaceae' (Halimedineae)	'Pseudochlorodesmis'
KU362182	Uncultured environmental Bryopsis clone JP3_F	Eukaryota	Chlorophyta	Ulvophyceae	Bryopsidales	Bryopsidaceae (Bryopsidineae)	Bryopsis
KU362183	Uncultured environmental Halimeda clone JP3_D	Eukaryota	Chlorophyta	Ulvophyceae	Bryopsidales	Halimedaceae (Halimedineae)	Halimeda
KU362184	Uncultured environmental Rhipiliaceae clone JP3_E	Eukaryota	Chlorophyta	Ulvophyceae	Bryopsidales	Rhipiliaceae (Halimedineae)	--
KU362185	Uncultured environmental Rhipiliaceae clone JP7_C	Eukaryota	Chlorophyta	Ulvophyceae	Bryopsidales	Rhipiliaceae (Halimedineae)	--
KU362186	Uncultured environmental Polyphysaceae clone JP1_B	Eukaryota	Chlorophyta	Ulvophyceae	Dasycladales	Polyphysaceae	Parvocaulis
KU362187	Uncultured environmental Pedinophyceae clone JP7_J	Eukaryota	Chlorophyta	Pedinophyceae	--	--	--
KU362188	Uncultured environmental Bangiaceae clone JP1_I	Eukaryota	Rhodophyta	Bangiophyceae	Bangiales	Bangiaceae	--
KU362189	Uncultured environmental 'Corallinaceae/Hapalidiaceae' clone JP25_E	Eukaryota	Rhodophyta	Florideophyceae	'Corallinales-Hapalidiales'	'Corallinaceae-Hapalidiaceae'	--
KU362190	Uncultured environmental 'Corallinaceae/Hapalidiaceae' clone JP3_N	Eukaryota	Rhodophyta	Florideophyceae	'Corallinales-Hapalidiales'	'Corallinaceae-Hapalidiaceae'	--
KU362191	Uncultured environmental 'Corallinaceae/Hapalidiaceae' clone JP6_H	Eukaryota	Rhodophyta	Florideophyceae	'Corallinales-Hapalidiales'	'Corallinaceae-Hapalidiaceae'	--
KU362192	Uncultured environmental 'Corallinaceae/Hapalidiaceae' clone JP6_I	Eukaryota	Rhodophyta	Florideophyceae	'Corallinales-Hapalidiales'	'Corallinaceae-Hapalidiaceae'	--

Genbank	Genbank Identifier	Domain	Phyla	Class	Order	Family (Suborder)	Genus
KU362193	Uncultured environmental 'Corallinaceae/Hapalidiaceae' clone JP6_J	Eukaryota	Rhodophyta	Florideophyceae	'Corallinales-Hapalidiales'	'Corallinaceae-Hapalidiaceae'	--
KU362194	Uncultured environmental 'Corallinaceae/Hapalidiaceae' clone JP6_K	Eukaryota	Rhodophyta	Florideophyceae	'Corallinales-Hapalidiales'	'Corallinaceae-Hapalidiaceae'	--
KU362195	Uncultured environmental 'Corallinaceae/Hapalidiaceae' clone JP3_M	Eukaryota	Rhodophyta	Florideophyceae	'Corallinales-Hapalidiales'	'Corallinaceae-Hapalidiaceae'	--
KU362196	Uncultured environmental 'Corallinaceae/Hapalidiaceae' clone JP4_C	Eukaryota	Rhodophyta	Florideophyceae	'Corallinales-Hapalidiales'	'Corallinaceae-Hapalidiaceae'	--
KU362197	Uncultured environmental Kallymeniaceae clone S15_C	Eukaryota	Rhodophyta	Florideophyceae	Gigartinales' s. lato	Kallymeniaceae	--
KU362198	Uncultured environmental Ceramiales clone JP3_L	Eukaryota	Rhodophyta	Florideophyceae	Ceramiales		--
KU362199	Uncultured environmental Peyssonneliaceae clone E09_C	Eukaryota	Rhodophyta	Florideophyceae	Peyssonneliales	Peyssonneliaceae	--
KU362200	Uncultured environmental Peyssonneliaceae clone JP25_O	Eukaryota	Rhodophyta	Florideophyceae	Peyssonneliales	Peyssonneliaceae	--
KU362201	Uncultured environmental Florideophyceae clone JP7_K	Eukaryota	Rhodophyta	Florideophyceae	--	--	--
KU362202	Uncultured environmental Rhodophyta clone S15_B	Eukaryota	Rhodophyta	--	--	--	--
KU362203	Uncultured environmental Cyanophyceae clone E09_D	Prokaryota	Cyanophyta	Cyanophyceae	--	--	--
KU362204	Uncultured environmental Cyanophyceae clone E09_E	Prokaryota	Cyanophyta	Cyanophyceae	--	--	--
KU362205	Uncultured environmental Cyanophyceae clone JP1_J	Prokaryota	Cyanophyta	Cyanophyceae	--	--	--
KU362206	Uncultured environmental Cyanophyceae clone JP1_K	Prokaryota	Cyanophyta	Cyanophyceae	--	--	--
KU362207	Uncultured environmental Cyanophyceae clone JP25_F	Prokaryota	Cyanophyta	Cyanophyceae	--	--	--
KU362208	Uncultured environmental Cyanophyceae clone JP25_G	Prokaryota	Cyanophyta	Cyanophyceae	--	--	--
KU362209	Uncultured environmental Cyanophyceae clone JP25_H	Prokaryota	Cyanophyta	Cyanophyceae	--	--	--
KU362210	Uncultured environmental Cyanophyceae clone JP25_I	Prokaryota	Cyanophyta	Cyanophyceae	--	--	--
KU362211	Uncultured environmental Cyanophyceae clone JP25_J	Prokaryota	Cyanophyta	Cyanophyceae	--	--	--
KU362212	Uncultured environmental Cyanophyceae clone JP25_K	Prokaryota	Cyanophyta	Cyanophyceae	--	--	--
KU362213	Uncultured environmental Cyanophyceae clone JP25_L	Prokaryota	Cyanophyta	Cyanophyceae	--	--	--
KU362214	Uncultured environmental Cyanophyceae clone JP25_M	Prokaryota	Cyanophyta	Cyanophyceae	--	--	--
KU362215	Uncultured environmental Cyanophyceae clone JP25_N	Prokaryota	Cyanophyta	Cyanophyceae	--	--	--
KU362216	Uncultured environmental Cyanophyceae clone JP25_P	Prokaryota	Cyanophyta	Cyanophyceae	--	--	--
KU362217	Uncultured environmental Cyanophyceae clone JP3_O	Prokaryota	Cyanophyta	Cyanophyceae	--	--	--
KU362218	Uncultured environmental Cyanophyceae clone JP3_P	Prokaryota	Cyanophyta	Cyanophyceae	--	--	--
KU362219	Uncultured environmental Cyanophyceae clone JP6_L	Prokaryota	Cyanophyta	Cyanophyceae	--	--	--
KU362220	Uncultured environmental Cyanophyceae clone JP6_M	Prokaryota	Cyanophyta	Cyanophyceae	--	--	--
KU362221	Uncultured environmental Cyanophyceae clone JP6_N	Prokaryota	Cyanophyta	Cyanophyceae	--	--	--
KU362222	Uncultured environmental Cyanophyceae clone JP6_O	Prokaryota	Cyanophyta	Cyanophyceae	--	--	--
KU362223	Uncultured environmental Cyanophyceae clone JP7_L	Prokaryota	Cyanophyta	Cyanophyceae	--	--	--
KU362224	Uncultured environmental Cyanophyceae clone JP7_M	Prokaryota	Cyanophyta	Cyanophyceae	--	--	--
KU362225	Uncultured environmental Cyanophyceae clone JP7_N	Prokaryota	Cyanophyta	Cyanophyceae	--	--	--
KU362226	Uncultured environmental Cyanophyceae clone JP7_O	Prokaryota	Cyanophyta	Cyanophyceae	--	--	--
KU362227	Uncultured environmental Cyanophyceae clone JP7_P	Prokaryota	Cyanophyta	Cyanophyceae	--	--	--
KU362228	Uncultured environmental Pavlovophyceae clone JP3_K	Eukaryota	Haptophyta	Pavlovophyceae	--	--	--
KU362229	Uncultured environmental 'Bacillariophyta' clone E09_B	Eukaryota	Ochrophyta	'Bacillariophyta'	--	--	--
KU362230	Uncultured environmental 'Bacillariophyta' clone JP25_D	Eukaryota	Ochrophyta	'Bacillariophyta'	--	--	--
KU362231	Uncultured environmental 'Bacillariophyta' clone JP4_A	Eukaryota	Ochrophyta	'Bacillariophyta'	--	--	--
KU362232	Uncultured environmental 'Bacillariophyta' clone JP4_B	Eukaryota	Ochrophyta	'Bacillariophyta'	--	--	--
KU362233	Uncultured environmental 'Bacillariophyta' clone JP6_F	Eukaryota	Ochrophyta	'Bacillariophyta'	--	--	--
KU362234	Uncultured environmental 'Bacillariophyta' clone JP6_G	Eukaryota	Ochrophyta	'Bacillariophyta'	--	--	--

Genbank	Genbank Identifier	Domain	Phyla	Class	Order	Family (Suborder)	Genus
KU362235	Uncultured environmental Pelagophyceae clone JP3_J	Eukaryota	Ochrophyta	Pelagophyceae	--	--	--
KU362236	Uncultured environmental Pelagophyceae clone JP6_E	Eukaryota	Ochrophyta	Pelagophyceae	--	--	--
