# Supplementary figures and images for: A metabarcoding framework for facilitated survey of endolithic phototrophs with tufA
Source: BMC Ecol. 2016 Mar 10;16:8. doi: 10.1186/s12898-016-0068-x (PMC4785743; doi:10.1186/s12898-016-0068-x)

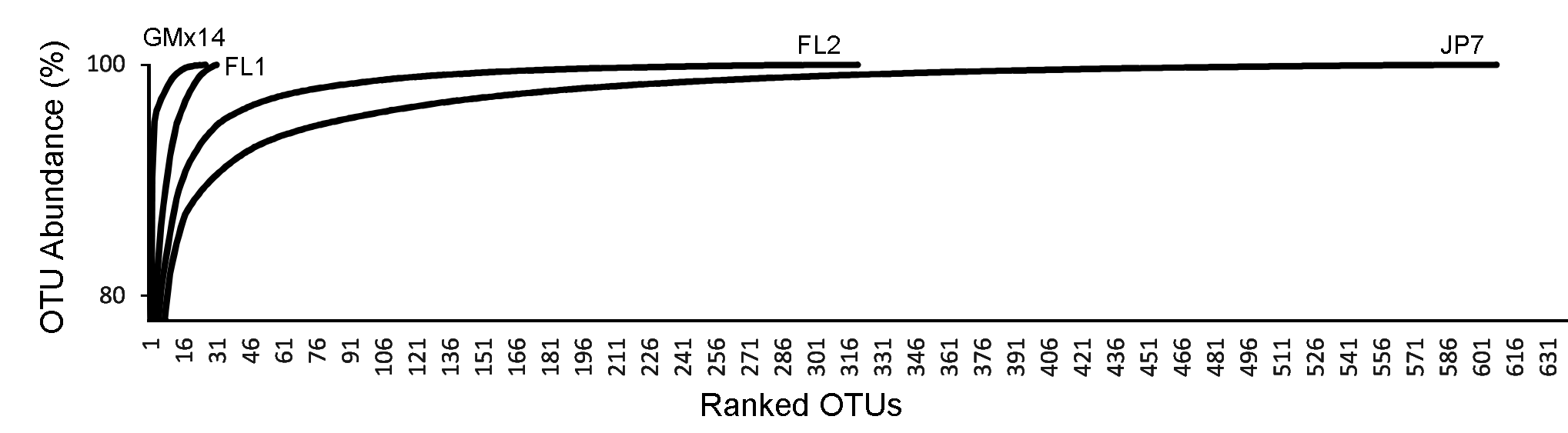

Supplement: Supplementary file 6 — 10.1186/s12898-016-0068-x Cumulative OTU-ranked abundance curves. Note the much larger OTU diversity in densely colonized JP07 and FL02 vs. lightly colonized microfloras FL01 and GM14. Cumulative abundances values below 80 % not displayed for figure succinctness. [file 12898_2016_68_MOESM6_ESM.tif]

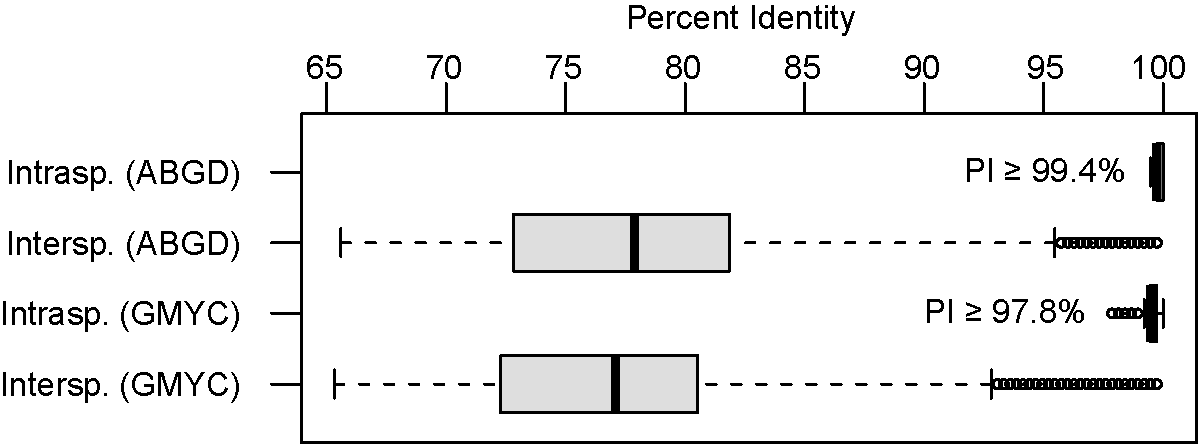

Supplement: Supplementary file 10 — 10.1186/s12898-016-0068-x Molecular species divergence in the Ulvophyceae. Distribution of intra- and interspecific divergence for molecular species of the orders Bryopsidales and ‘Ulvales-Ulothrichales’ delimited with ABGD and GMYC. Percent identity values were computed based on an alignment of 375 bp of the tufA metabarcode. Maximum thresholds for molecular species classification/annotation are printed. PI percent identity. [file 12898_2016_68_MOESM10_ESM.tif]

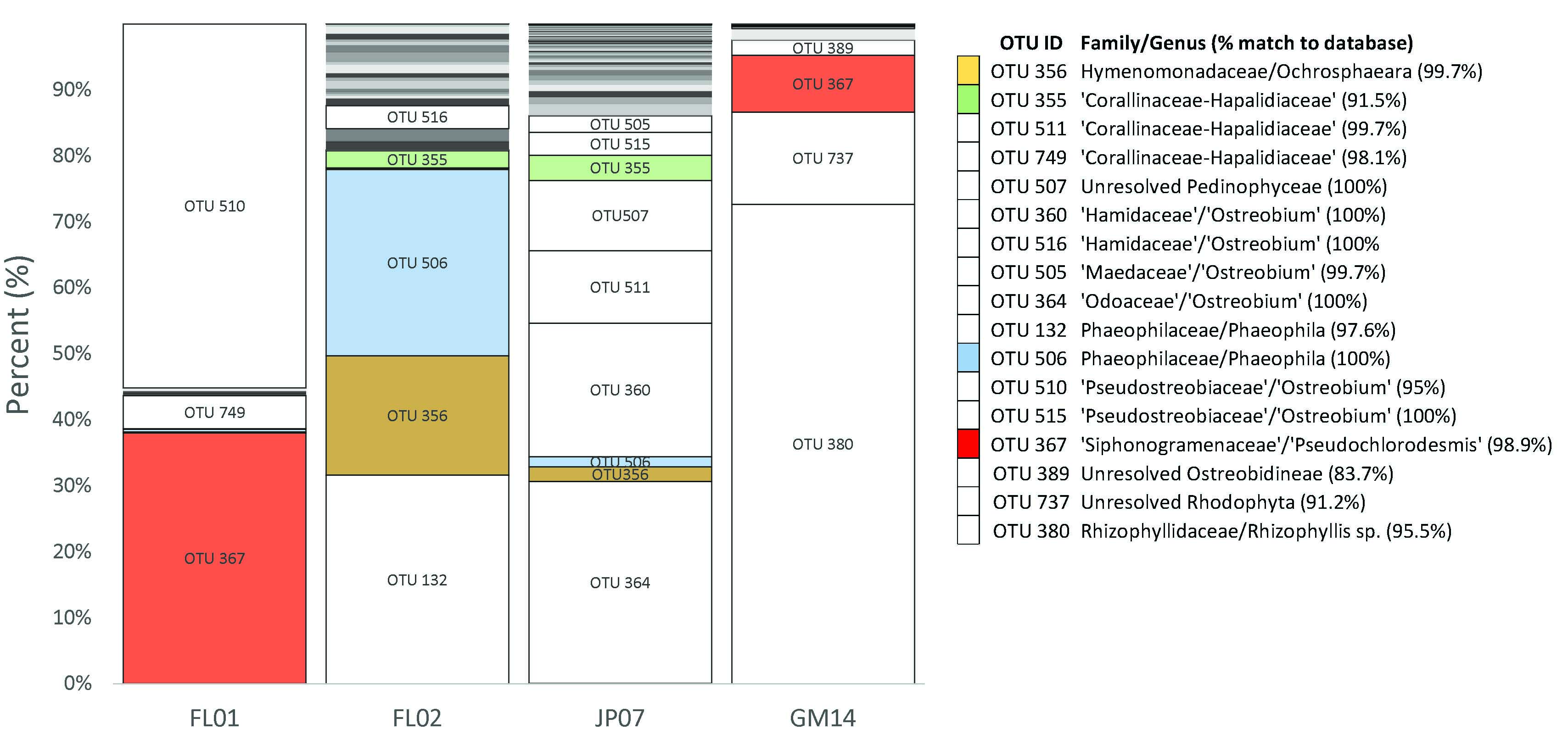

Supplement: Supplementary file 11 — 10.1186/s12898-016-0068-x OTU abundance profiles. OTUs underlying abundance patterns in Fig. 13. OTUs found in large read abundance across multiple microflora samples are color-coded in blue, green, orange and red. Other abundant OTUs are color-coded in white and those below 2 % abundance in shades of grey. The OTUs low-level taxonomy and their percent match to the database are listed in the legend. [file 12898_2016_68_MOESM11_ESM.jpg]

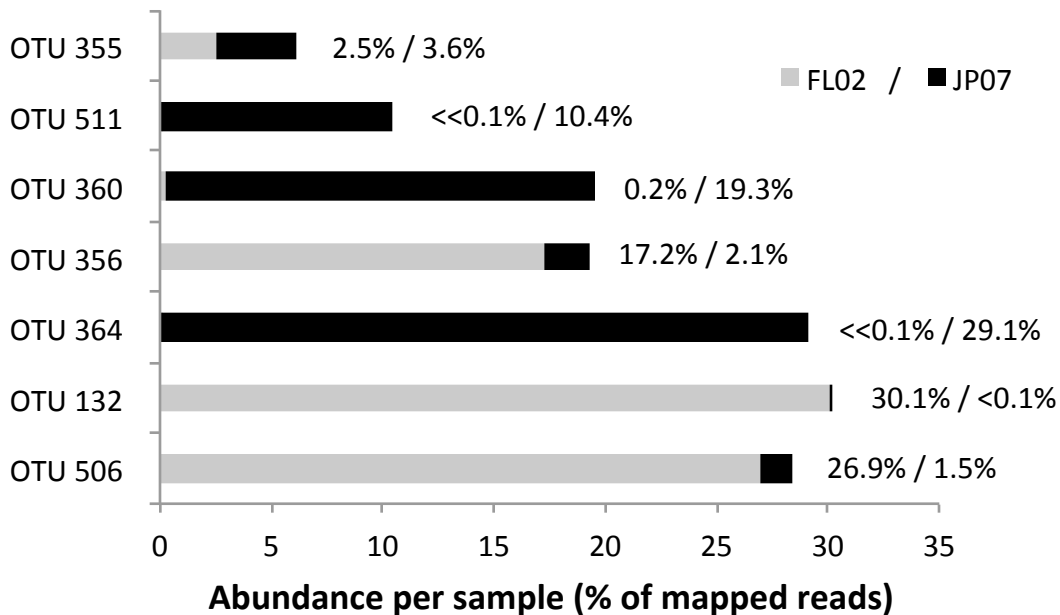

Supplement: Supplementary file 12 — 10.1186/s12898-016-0068-x Example of putative index tag jumping or tag error. Comparative read abundance for seven OTUs demultiplexed (i.e. assigned) to both FL02 and JP07. For illustration purpose, only OTUs with >5 % of mapped reads in either FL02 or JP07 are shown (these correspond to seven out of the 141 common OTUs, see Additional file 9: Figure S2). Note the drastic abundance disparity illustrative of or potential of false assignment via index tag jumping or tag error (e.g. OTU 511 representing ≪0.1 % reads in FL02 and 10.4 % of reads in JP07). By contrast, note the more balanced abundance of OTU 355, 356, and 506, which may reflect the true presence of common OTUs in both samples (i.e. pantropical OTUs). [file 12898_2016_68_MOESM12_ESM.pdf]

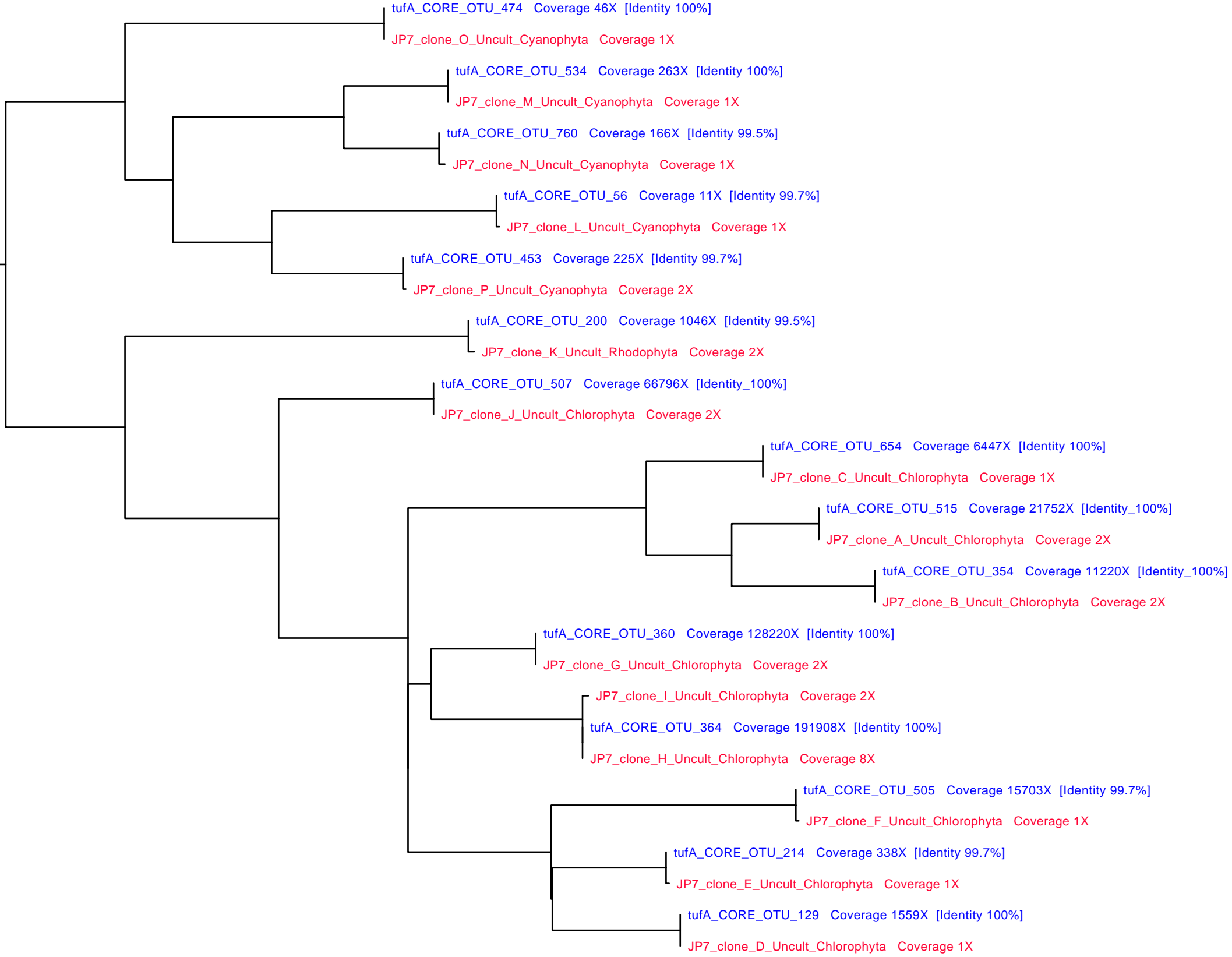

0.07

Supplement: Supplementary file 13 — 10.1186/s12898-016-0068-x OTUs vs. clone identity and coverage. Comparison of all phototrophic clones obtained from JP07 with their matching OTUs. Note high identity of clones and OTUs based on 375 bp, with 0, 1 or 2 base pair variation (100, 99.7 and 99.5 % respectively). Also note that clone H and its corresponding OTU_364 share the highest coverage, although further cloning would be necessary to accurately assess congruence in sequence coverage between the two methods. [file 12898_2016_68_MOESM13_ESM.pdf]

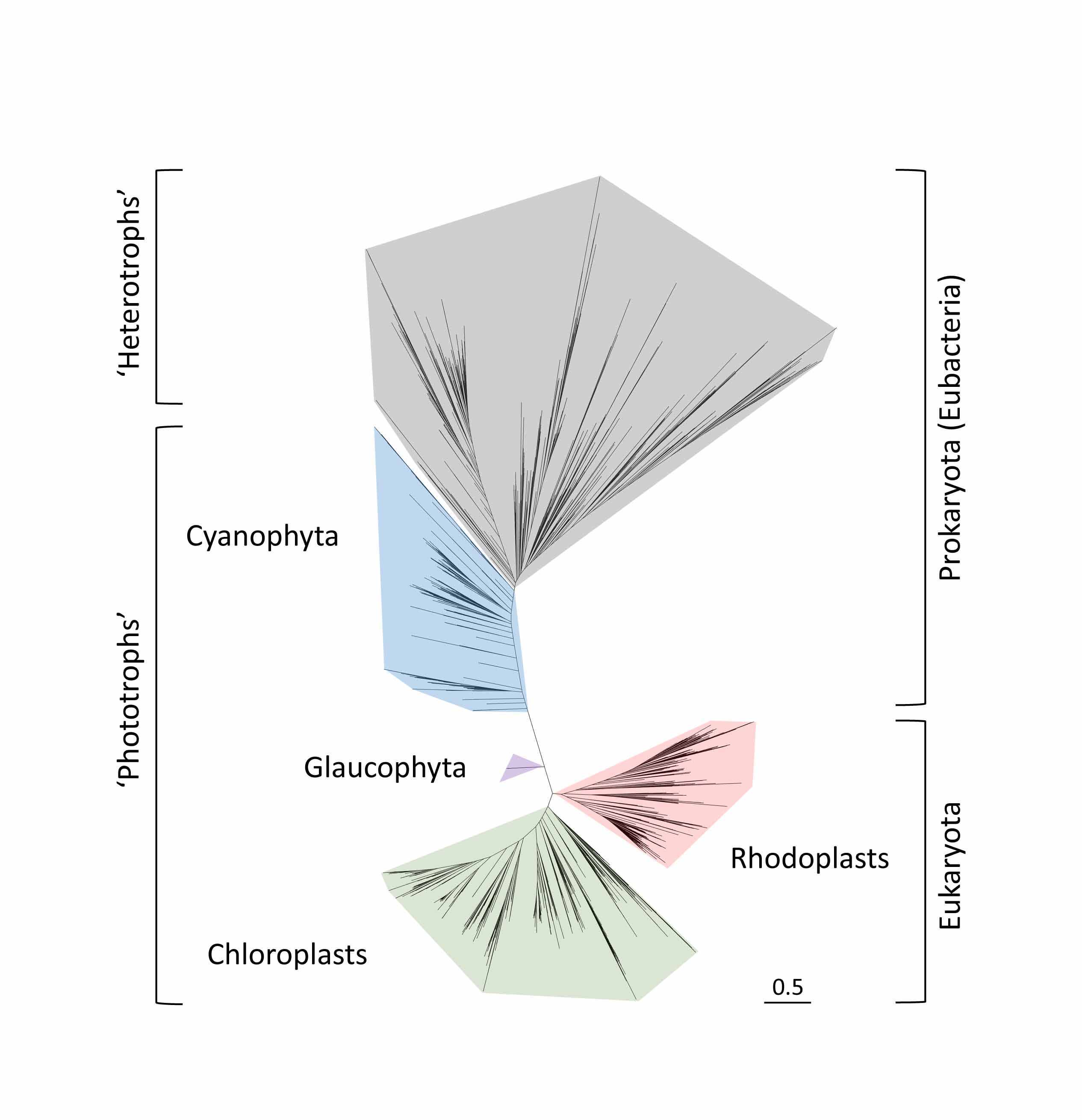

Supplement: Supplementary file 14 — 10.1186/s12898-016-0068-x tufA database and higher taxonomy overview. Unrooted RAxML tree comprising 556 ‘Heterotrophs’and 2141 ‘Phototrophs’ tufA sequences aligned on 891 bp. ‘Heterotrophs’ include multiple phyla of the Eubacteria. ‘Phototrophs’ include the prokaryotic eubacterial phylum Cyanophyta and several eukaryotic phyla, including the Archaeplastida (Chlorophyta, Glaucophyta and Rhodophyta) and secondary endosymbiotic phyla. ‘Chloroplasts’ include the Chlorophyta and the secondary endosymbiotic phyla Euglenophyta and Chlorarachniophyta. ‘Rhodoplasts’ include the Rhodophyta and the secondary endosymbiotic phyla of the Cryptophyta, Haptophyta and Ochrophyta. [file 12898_2016_68_MOESM14_ESM.jpg]
