# Supplementary material for: A metabarcoding framework for facilitated survey of endolithic phototrophs with tufA
Source: BMC Ecol. 2016 Mar 10;16:8. doi: 10.1186/s12898-016-0068-x (PMC4785743; doi:10.1186/s12898-016-0068-x)
Supplement: Supplementary file 7 — 10.1186/s12898-016-0068-x Summary of microfloras richness and abundance at multiple taxonomic levels. (A–B) OTU counts and read abundance for ‘Phototrophs’ vs. ‘Heterotrophs’ group. (C–D) OTU counts and read abundance for phototrophic phyla and classes. (E–F) OTU counts and read abundance for Ulvophyceaen families. Note that OTUs may be found more than once across samples. [file 12898_2016_68_MOESM7_ESM.pdf]

A

Category		All	FL01	FL02	JP07	GM14	All%	FL01%	FL02%	JP07%	GM14%
'Heterotrophs'		184	4	62	122	14	22.9	15.4	19.4	20.1	46.7
'Phototrophs'		618	22	258	486	16	77.1	84.6	80.6	79.9	53.3
	**Database Σ**	**802**	**26**	**320**	**608**	**30**	**100**	**100**	**100**	**100**	**100**

B

Category		All	FL01	FL02	JP07	GM14	All%	FL01%	FL02%	JP07%	GM14%
'Heterotrophs'		26233	139	10845	14176	1073	2.1	0.6	1.9	2.1	16.8
'Phototrophs'		1234574	23646	547658	657943	5327	97.9	99.4	98.1	97.9	83.2
	**Database Σ**	**1260807**	**23785**	**558503**	**672119**	**6400**	**100**	**100**	**100**	**100**	**100**

C

Phylum	Class	All	FL01	FL02	JP07	GM14	All%	FL01%	FL02%	JP07%	GM14%
'Chlorarachniophyta'	'Chlorarachniophyceae'	9	0	6	6	0	1.5	0.0	2.3	1.2	0.0
Chlorophyta	Pedinophyceae	1	0	1	1	0	0.2	0.0	0.4	0.2	0.0
	Prasinophyceae	7	0	6	2	0	1.1	0.0	2.3	0.4	0.0
	Ulvophyceae	97	11	48	84	11	15.7	50.0	18.6	17.3	68.8
	Unres. Chlorophyta	1	0	0	0	1	0.2	0.0	0.0	0.0	6.3
	Σ	106	11	55	87	12	17.2	50.0	21.3	17.9	75.0
Cryptophyta	Cryptophyceae	2	0	0	2	0	0.3	0.0	0.0	0.4	0.0
Cyanophyta	Cyanophyceae	267	4	76	231	0	43.2	18.2	29.5	47.5	0.0
Haptophyta	Pavlovophyceae	1	0	1	0	0	0.2	0.0	0.4	0.0	0.0
	Coccolithophyceae	2	1	1	2	1	0.3	4.5	0.4	0.4	6.3
	Unres. Haptophyta	1	0	1	0	0	0.2	0.0	0.4	0.0	0.0
	Σ	4	1	3	2	1	0.6	4.5	1.2	0.4	6.3
Ochrophyta	'Bacillariophyta'	162	1	89	113	0	26.2	4.5	34.5	23.3	0.0
	Phaeophyceae	12	0	4	9	0	1.9	0.0	1.6	1.9	0.0
	Unres. Ochrophyta	18	0	10	11	0	2.9	0.0	3.9	2.3	0.0
	Σ	192	1	103	133	0	31.1	4.5	39.9	27.4	0.0
Rhodophyta	Bangiophyceae	1	0	0	1	0	0.2	0.0	0.0	0.2	0.0
	Compsopogonophyceae	1	0	1	0	0	0.2	0.0	0.4	0.0	0.0
	Florideophyceae	33	5	13	22	2	5.3	22.7	5.0	4.5	12.5
	Stylonematophyceae	1	0	1	1	0	0.2	0.0	0.4	0.2	0.0
	Unres. Rhodophyta	2	0	0	1	1	0.3	0.0	0.0	0.2	6.3
	Σ	38	5	15	25	3	6.1	22.7	5.8	5.1	18.8
	'Phototrophs' Σ	618	22	258	486	16	100	100	100	100	100

D

Phylum	Class	All	FL01	FL02	JP07	GM14	All%	FL01%	FL02%	JP07%	GM14%
'Chlorarachniophyta'	'Chlorarachniophyceae'	3598	0	3116	482	0	0.3	0.0	0.6	0.1	0.0
Chlorophyta	Pedinophyceae	66796	0	52	66744	0	5.4	0.0	0.0	10.1	0.0
	Prasinophyceae	825	0	766	59	0	0.1	0.0	0.1	0.0	0.0
	Ulvophyceae	824135	22083	374177	427067	808	66.8	93.4	68.3	64.9	15.2
	Unres. Chlorophyta	22	0	0	0	22	0.0	0.0	0.0	0.0	0.4
	Σ	891778	22083	374995	493870	830	72.2	93.4	68.5	75.1	15.6
Cryptophyta	Cryptophyceae	43	0	0	43	0	0.0	0.0	0.0	0.0	0.0
Cyanophyta	Cyanophyceae	57912	143	29735	28034	0	4.7	0.6	5.4	4.3	0.0
Haptophyta	Pavlovophyceae	36	0	36	0	0	0.0	0.0	0.0	0.0	0.0
	Coccolithophyceae	108211	33	94473	13697	8	8.8	0.1	17.3	2.1	0.2
	Unres. Haptophyta	138	0	138	0	0	0.0	0.0	0.0	0.0	0.0
	Σ	108385	33	94647	13697	8	8.8	0.1	17.3	2.1	0.2
Ochrophyta	'Bacillariophyta'	22845	42	9326	13477	0	1.9	0.2	1.7	2.0	0.0
	Phaeophyceae	2429	0	408	2021	0	0.2	0.0	0.1	0.3	0.0
	Unres. Ochrophyta	2099	0	544	1555	0	0.2	0.0	0.1	0.2	0.0
	Σ	27373	42	10278	17053	0	2.2	0.2	1.9	2.6	0.0
Rhodophyta	Bangiophyceae	623	0	0	623	0	0.1	0.0	0.0	0.1	0.0
	Compsopogonophyceae	43	0	43	0	0	0.0	0.0	0.0	0.0	0.0
	Florideophyceae	142371	1345	34840	102409	3777	11.5	5.7	6.4	15.6	70.9
	Stylonematophyceae	1533	0	4	1529	0	0.1	0.0	0.0	0.2	0.0
	Unres. Rhodophyta	915	0	0	203	712	0.1	0.0	0.0	0.0	13.4
	Σ	145485	1345	34887	104764	4489	11.8	5.7	6.4	15.9	84.3
	'Phototrophs' Σ	1234574	23646	547658	657943	5327	100	100	100	100	100

E

Order/Suborder	Family	All	FL01	FL02	JP07	GM14	All%	FL01%	FL02%	JP07%	GM14%
Bryopsidales/Bryopsidineae	Bryopsidaceae	6	0	5	7	0	6.2	0.0	10.4	8.3	0.0
	Derbesiaceae	1	0	0	6	0	1.0	0.0	0.0	7.1	0.0
	Σ	14	0	5	13	0	14.4	0.0	10.4	15.5	0.0
Bryopsidales/Halimedineae	'Pseudochlorodesmidaceae'	1	0	2	2	0	1.0	0.0	4.2	2.4	0.0
	'Pseudostreobiaceae'	1	2	4	9	0	1.0	18.2	8.3	10.7	0.0
	'Siphonogramenaceae'	8	1	0	0	1	8.2	9.1	0.0	0.0	9.1
	Caulerpaceae	3	0	1	1	0	3.1	0.0	2.1	1.2	0.0
	Halimedaceae	1	0	0	1	0	1.0	0	0	1.2	0
	Rhipiliaceae	10	0	2	3	0	10.3	0.0	4.2	3.6	0.0
	Σ	17	3	9	16	1	17.5	27.3	18.8	19.0	9.1
Bryopsidales/Ostreobidineae	'Hamidaceae'	2	1	3	2	0	2.1	9.1	6.3	2.4	0.0
	'Maedaceae'	2	5	14	28	3	2.1	45.5	29.2	33.3	27.3
	'Odoaceae'	9	0	1	2	1	9.3	0.0	2.1	2.4	9.1
	'Unarizakiaceae'	1	0	0	1	0	1.0	0.0	0.0	1.2	0.0
	Unres. Ostreobidineae	11	0	2	6	4	11.3	0.0	4.2	7.1	36.4
	Σ	48	6	20	39	8	49.5	54.5	41.7	46.4	72.7
'Ulvales-Ulothrichales'	Phaeophilaceae	11	2	3	4	2	11.3	18.2	6.3	4.8	18.2
	Ulvaceae	11	0	1	1	0	11.3	0.0	2.1	1.2	0.0
	Ulvellaceae	3	0	8	10	0	3.1	0.0	16.7	11.9	0.0
	Unres. 'Ulothrichales-Ulvales'	3	0	2	1	0	3.1	0.0	4.2	1.2	0.0
	Σ	18	2	14	16	2	18.6	18.2	29.2	19.0	18.2
	Ulvophyceae Σ	97	11	48	84	11	100	100	100	100	100

F

Order/Suborder	Family	All	FL01	FL02	JP07	GM14	All%	FL01%	FL02%	JP07%	GM14%
Bryopsidales/Bryopsidineae	Bryopsidaceae	8423	0	2391	6032	0	1.0	0.0	0.6	1.4	0.0
	Derbesiaceae	720	0	0	720	0	0.1	0.0	0.0	0.2	0.0
	Σ	9143	0	2391	6752	0	1.1	0.0	0.6	1.6	0.0
Bryopsidales/Halimedineae	'Pseudochlorodesmidaceae'	6017	0	17	6000	0	0.7	0.0	0.0	1.4	0.0
	'Pseudostreobiaceae'	52498	12715	51	39732	0	6.4	57.6	0.0	9.3	0.0
	'Siphonogramenaceae'	9183	8748	0	0	435	1.1	39.6	0.0	0.0	53.8
	Caulerpaceae	4696	0	8	4688	0	0.6	0.0	0.0	1.1	0.0
	Halimedaceae	25	0	0	25	0	0.0	0.0	0.0	0.0	0.0
	Rhipiliaceae	8057	0	16	8041	0	1.0	0.0	0.0	1.9	0.0
	Σ	80476	21463	92	58486	435	9.8	97.2	0.0	13.7	53.8
Bryopsidales/Ostreobidineae	'Hamidaceae'	146827	4	19495	127328	0	17.8	0.0	5.2	29.8	0.0
	'Maedaceae'	56579	510	26550	29409	110	6.9	2.3	7.1	6.9	13.6
	'Odoaceae'	192851	0	55	192787	9	23.4	0.0	0.0	45.1	1.1
	'Unarizakiaceae'	49	0	0	49	0	0.0	0.0	0.0	0.0	0.0
	Unres. Ostreobidineae	1487	0	39	1213	235	0.2	0.0	0.0	0.3	29.1
	Σ	397793	514	46139	350786	354	48.3	2.3	12.3	82.1	43.8
'Ulvales-Ulothrichales'	Phaeophilaceae	323815	106	313724	9966	19	39.3	0.5	83.8	2.3	2.4
	Ulvaceae	183	0	57	126	0	0.0	0.0	0.0	0.0	0.0
	Ulvellaceae	12562	0	11671	891	0	1.5	0.0	3.1	0.2	0.0
	Unres. 'Ulothrichales-Ulvales'	163	0	103	60	0	0.0	0.0	0.0	0.0	0.0
	Σ	336723	106	325555	11043	19	40.9	0.5	87.0	2.6	2.4
	Ulvophyceae Σ	824135	22083	374177	427067	808	100	100	100	100	100
