# Supplementary material for: A metabarcoding framework for facilitated survey of endolithic phototrophs with tufA
Source: BMC Ecol. 2016 Mar 10;16:8. doi: 10.1186/s12898-016-0068-x (PMC4785743; doi:10.1186/s12898-016-0068-x)
Supplement: Supplementary file 8 — 10.1186/s12898-016-0068-x OTUs taxonomy and their microflora assignment. Detailed reference taxonomy for 802 core OTUs as determined from their phylogenetic position (see Fig. 6). Taxonomic groupings with unsettled naming such as combined orders, provisional families, and polyphyletic or monophyletic species or genera complexes, are noted between quotes. [file 12898_2016_68_MOESM8_ESM.pdf]

Identifier	Function	Domain	Phyla	Class	Order	Family (Suborder)	Genus	FL01	FL02	JP07	GM14
OTU 529	Photot.	Euk.	'Chlorarachniophyta'	'Chlorarachniophyceae'	--	--	--		+	+	
OTU 676	Photot.	Euk.	'Chlorarachniophyta'	'Chlorarachniophyceae'	--	--	--		+	+	
OTU 745	Photot.	Euk.	'Chlorarachniophyta'	'Chlorarachniophyceae'	--	--	--		+	+	
OTU 241	Photot.	Euk.	'Chlorarachniophyta'	'Chlorarachniophyceae'	--	--	--			+	
OTU 604	Photot.	Euk.	'Chlorarachniophyta'	'Chlorarachniophyceae'	--	--	--			+	
OTU 318	Photot.	Euk.	'Chlorarachniophyta'	'Chlorarachniophyceae'	--	--	--			+	
OTU 272	Photot.	Euk.	'Chlorarachniophyta'	'Chlorarachniophyceae'	--	--	--		+		
OTU 013	Photot.	Euk.	'Chlorarachniophyta'	'Chlorarachniophyceae'	--	--	--		+		
OTU 107	Photot.	Euk.	'Chlorarachniophyta'	'Chlorarachniophyceae'	--	--	--		+		
OTU 507	Photot.	Euk.	Chlorophyta	Pedinophyceae	--	--	--		+	+	
OTU 195	Photot.	Euk.	Chlorophyta	Prasinophyceae	--	--	--		+	+	
OTU 089	Photot.	Euk.	Chlorophyta	Prasinophyceae	--	--	--				+
OTU 659	Photot.	Euk.	Chlorophyta	Prasinophyceae	--	--	--			+	
OTU 580	Photot.	Euk.	Chlorophyta	Prasinophyceae	--	--	--		+		
OTU 712	Photot.	Euk.	Chlorophyta	Prasinophyceae	--	--	--		+		
OTU 221	Photot.	Euk.	Chlorophyta	Prasinophyceae	--	--	--		+		
OTU 185	Photot.	Euk.	Chlorophyta	Prasinophyceae	--	--	--		+		
OTU 393	Photot.	Euk.	Chlorophyta	Prasinophyceae	--	--	--		+		
OTU 614	Photot.	Euk.	Chlorophyta	Ulvophyceae	'Ulvales-Ulothrichales'	--	--			+	
OTU 010	Photot.	Euk.	Chlorophyta	Ulvophyceae	'Ulvales-Ulothrichales'	--	--		+		
OTU 048	Photot.	Euk.	Chlorophyta	Ulvophyceae	'Ulvales-Ulothrichales'	--	--		+		
OTU 493	Photot.	Euk.	Chlorophyta	Ulvophyceae	'Ulvales-Ulothrichales'	Phaeophilaceae	Phaeophila		+	+	
OTU 506	Photot.	Euk.	Chlorophyta	Ulvophyceae	'Ulvales-Ulothrichales'	Phaeophilaceae	Phaeophila	+	+	+	+
OTU 132	Photot.	Euk.	Chlorophyta	Ulvophyceae	'Ulvales-Ulothrichales'	Phaeophilaceae	Phaeophila	+	+	+	+
OTU 625	Photot.	Euk.	Chlorophyta	Ulvophyceae	'Ulvales-Ulothrichales'	Phaeophilaceae	Phaeophila			+	
OTU 040	Photot.	Euk.	Chlorophyta	Ulvophyceae	'Ulvales-Ulothrichales'	Ulvaceae	Ulva		+	+	
OTU 114	Photot.	Euk.	Chlorophyta	Ulvophyceae	'Ulvales-Ulothrichales'	Ulvellaceae	Ulvella		+	+	
OTU 440	Photot.	Euk.	Chlorophyta	Ulvophyceae	'Ulvales-Ulothrichales'	Ulvellaceae	Ulvella		+	+	
OTU 209	Photot.	Euk.	Chlorophyta	Ulvophyceae	'Ulvales-Ulothrichales'	Ulvellaceae	Ulvella		+	+	
OTU 757	Photot.	Euk.	Chlorophyta	Ulvophyceae	'Ulvales-Ulothrichales'	Ulvellaceae	Ulvella		+	+	
OTU 340	Photot.	Euk.	Chlorophyta	Ulvophyceae	'Ulvales-Ulothrichales'	Ulvellaceae	Ulvella		+	+	
OTU 766	Photot.	Euk.	Chlorophyta	Ulvophyceae	'Ulvales-Ulothrichales'	Ulvellaceae	Ulvella		+	+	
OTU 508	Photot.	Euk.	Chlorophyta	Ulvophyceae	'Ulvales-Ulothrichales'	Ulvellaceae	Ulvella		+	+	
OTU 135	Photot.	Euk.	Chlorophyta	Ulvophyceae	'Ulvales-Ulothrichales'	Ulvellaceae	Ulvella		+	+	
OTU 538	Photot.	Euk.	Chlorophyta	Ulvophyceae	'Ulvales-Ulothrichales'	Ulvellaceae	Ulvella			+	
OTU 478	Photot.	Euk.	Chlorophyta	Ulvophyceae	'Ulvales-Ulothrichales'	Ulvellaceae	Ulvella			+	
OTU 360	Photot.	Euk.	Chlorophyta	Ulvophyceae	Bryopsidales	'Hamidaceae' (Ostreobidineae)	'Ostreobium'	+	+	+	
OTU 516	Photot.	Euk.	Chlorophyta	Ulvophyceae	Bryopsidales	'Hamidaceae' (Ostreobidineae)	'Ostreobium'		+	+	
OTU 183	Photot.	Euk.	Chlorophyta	Ulvophyceae	Bryopsidales	'Hamidaceae' (Ostreobidineae)	'Ostreobium'		+		
OTU 127	Photot.	Euk.	Chlorophyta	Ulvophyceae	Bryopsidales	'Maedaceae' (Ostreobidineae)	'Ostreobium'		+	+	
OTU 335	Photot.	Euk.	Chlorophyta	Ulvophyceae	Bryopsidales	'Maedaceae' (Ostreobidineae)	'Ostreobium'		+	+	
OTU 129	Photot.	Euk.	Chlorophyta	Ulvophyceae	Bryopsidales	'Maedaceae' (Ostreobidineae)	'Ostreobium'		+	+	
OTU 376	Photot.	Euk.	Chlorophyta	Ulvophyceae	Bryopsidales	'Maedaceae' (Ostreobidineae)	'Ostreobium'		+	+	
OTU 505	Photot.	Euk.	Chlorophyta	Ulvophyceae	Bryopsidales	'Maedaceae' (Ostreobidineae)	'Ostreobium'		+	+	
OTU 248	Photot.	Euk.	Chlorophyta	Ulvophyceae	Bryopsidales	'Maedaceae' (Ostreobidineae)	'Ostreobium'		+	+	
OTU 329	Photot.	Euk.	Chlorophyta	Ulvophyceae	Bryopsidales	'Maedaceae' (Ostreobidineae)	'Ostreobium'		+	+	
OTU 370	Photot.	Euk.	Chlorophyta	Ulvophyceae	Bryopsidales	'Maedaceae' (Ostreobidineae)	'Ostreobium'	+	+	+	+
OTU 512	Photot.	Euk.	Chlorophyta	Ulvophyceae	Bryopsidales	'Maedaceae' (Ostreobidineae)	'Ostreobium'		+	+	
OTU 491	Photot.	Euk.	Chlorophyta	Ulvophyceae	Bryopsidales	'Maedaceae' (Ostreobidineae)	'Ostreobium'	+	+	+	
OTU 134	Photot.	Euk.	Chlorophyta	Ulvophyceae	Bryopsidales	'Maedaceae' (Ostreobidineae)	'Ostreobium'		+	+	
OTU 377	Photot.	Euk.	Chlorophyta	Ulvophyceae	Bryopsidales	'Maedaceae' (Ostreobidineae)	'Ostreobium'	+	+	+	
OTU 795	Photot.	Euk.	Chlorophyta	Ulvophyceae	Bryopsidales	'Maedaceae' (Ostreobidineae)	'Ostreobium'	+	+	+	
OTU 488	Photot.	Euk.	Chlorophyta	Ulvophyceae	Bryopsidales	'Maedaceae' (Ostreobidineae)	'Ostreobium'	+		+	
OTU 298	Photot.	Euk.	Chlorophyta	Ulvophyceae	Bryopsidales	'Maedaceae' (Ostreobidineae)	'Ostreobium'				+
OTU 398	Photot.	Euk.	Chlorophyta	Ulvophyceae	Bryopsidales	'Maedaceae' (Ostreobidineae)	'Ostreobium'				+
OTU 212	Photot.	Euk.	Chlorophyta	Ulvophyceae	Bryopsidales	'Maedaceae' (Ostreobidineae)	'Ostreobium'			+	
OTU 736	Photot.	Euk.	Chlorophyta	Ulvophyceae	Bryopsidales	'Maedaceae' (Ostreobidineae)	'Ostreobium'			+	
OTU 418	Photot.	Euk.	Chlorophyta	Ulvophyceae	Bryopsidales	'Maedaceae' (Ostreobidineae)	'Ostreobium'			+	
OTU 214	Photot.	Euk.	Chlorophyta	Ulvophyceae	Bryopsidales	'Maedaceae' (Ostreobidineae)	'Ostreobium'			+	
OTU 410	Photot.	Euk.	Chlorophyta	Ulvophyceae	Bryopsidales	'Maedaceae' (Ostreobidineae)	'Ostreobium'			+	
OTU 406	Photot.	Euk.	Chlorophyta	Ulvophyceae	Bryopsidales	'Maedaceae' (Ostreobidineae)	'Ostreobium'			+	
OTU 688	Photot.	Euk.	Chlorophyta	Ulvophyceae	Bryopsidales	'Maedaceae' (Ostreobidineae)	'Ostreobium'			+	
OTU 781	Photot.	Euk.	Chlorophyta	Ulvophyceae	Bryopsidales	'Maedaceae' (Ostreobidineae)	'Ostreobium'			+	
OTU 574	Photot.	Euk.	Chlorophyta	Ulvophyceae	Bryopsidales	'Maedaceae' (Ostreobidineae)	'Ostreobium'			+	
OTU 622	Photot.	Euk.	Chlorophyta	Ulvophyceae	Bryopsidales	'Maedaceae' (Ostreobidineae)	'Ostreobium'			+	
OTU 586	Photot.	Euk.	Chlorophyta	Ulvophyceae	Bryopsidales	'Maedaceae' (Ostreobidineae)	'Ostreobium'			+	

Identifier	Function	Domain	Phyla	Class	Order	Family (Suborder)	Genus	FL01	FL02	JP07	GM14
OTU 456	Photot.	Euk.	Chlorophyta	Ulvophyceae	Bryopsidales	'Maedaceae' (Ostreobidineae)	'Ostreobium'			+	
OTU 451	Photot.	Euk.	Chlorophyta	Ulvophyceae	Bryopsidales	'Maedaceae' (Ostreobidineae)	'Ostreobium'			+	
OTU 057	Photot.	Euk.	Chlorophyta	Ulvophyceae	Bryopsidales	'Maedaceae' (Ostreobidineae)	'Ostreobium'			+	
OTU 409	Photot.	Euk.	Chlorophyta	Ulvophyceae	Bryopsidales	'Maedaceae' (Ostreobidineae)	'Ostreobium'		+		
OTU 364	Photot.	Euk.	Chlorophyta	Ulvophyceae	Bryopsidales	'Odoaceae' (Ostreobidineae)	'Ostreobium'		+	+	+
OTU 202	Photot.	Euk.	Chlorophyta	Ulvophyceae	Bryopsidales	'Odoaceae' (Ostreobidineae)	'Ostreobium'			+	
OTU 330	Photot.	Euk.	Chlorophyta	Ulvophyceae	Bryopsidales	Unresolved (Ostreobidineae)	'Ostreobium'		+	+	
OTU 698	Photot.	Euk.	Chlorophyta	Ulvophyceae	Bryopsidales	Unresolved (Ostreobidineae)	'Ostreobium'				+
OTU 227	Photot.	Euk.	Chlorophyta	Ulvophyceae	Bryopsidales	Unresolved (Ostreobidineae)	'Ostreobium'				+
OTU 385	Photot.	Euk.	Chlorophyta	Ulvophyceae	Bryopsidales	Unresolved (Ostreobidineae)	'Ostreobium'				+
OTU 389	Photot.	Euk.	Chlorophyta	Ulvophyceae	Bryopsidales	Unresolved (Ostreobidineae)	'Ostreobium'				+
OTU 499	Photot.	Euk.	Chlorophyta	Ulvophyceae	Bryopsidales	Unresolved (Ostreobidineae)	'Ostreobium'			+	
OTU 754	Photot.	Euk.	Chlorophyta	Ulvophyceae	Bryopsidales	Unresolved (Ostreobidineae)	'Ostreobium'			+	
OTU 476	Photot.	Euk.	Chlorophyta	Ulvophyceae	Bryopsidales	Unresolved (Ostreobidineae)	'Ostreobium'			+	
OTU 002	Photot.	Euk.	Chlorophyta	Ulvophyceae	Bryopsidales	Unresolved (Ostreobidineae)	'Ostreobium'			+	
OTU 072	Photot.	Euk.	Chlorophyta	Ulvophyceae	Bryopsidales	Unresolved (Ostreobidineae)	'Ostreobium'			+	
OTU 558	Photot.	Euk.	Chlorophyta	Ulvophyceae	Bryopsidales	Unresolved (Ostreobidineae)	'Ostreobium'		+		
OTU 384	Photot.	Euk.	Chlorophyta	Ulvophyceae	Bryopsidales	'Unarizakiaceae' (Ostreobidineae)	'Ostreobium'			+	
OTU 517	Photot.	Euk.	Chlorophyta	Ulvophyceae	Bryopsidales	'Pseudostreobiaceae' (Halimedineae)	'Ostreobium'	+	+	+	
OTU 510	Photot.	Euk.	Chlorophyta	Ulvophyceae	Bryopsidales	'Pseudostreobiaceae' (Halimedineae)	'Ostreobium'	+	+	+	
OTU 515	Photot.	Euk.	Chlorophyta	Ulvophyceae	Bryopsidales	'Pseudostreobiaceae' (Halimedineae)	'Ostreobium'		+	+	
OTU 354	Photot.	Euk.	Chlorophyta	Ulvophyceae	Bryopsidales	'Pseudostreobiaceae' (Halimedineae)	'Ostreobium'		+	+	
OTU 500	Photot.	Euk.	Chlorophyta	Ulvophyceae	Bryopsidales	'Pseudostreobiaceae' (Halimedineae)	'Ostreobium'			+	
OTU 184	Photot.	Euk.	Chlorophyta	Ulvophyceae	Bryopsidales	'Pseudostreobiaceae' (Halimedineae)	'Ostreobium'			+	
OTU 434	Photot.	Euk.	Chlorophyta	Ulvophyceae	Bryopsidales	'Pseudostreobiaceae' (Halimedineae)	'Ostreobium'			+	
OTU 663	Photot.	Euk.	Chlorophyta	Ulvophyceae	Bryopsidales	'Pseudostreobiaceae' (Halimedineae)	'Ostreobium'			+	
OTU 236	Photot.	Euk.	Chlorophyta	Ulvophyceae	Bryopsidales	'Pseudostreobiaceae' (Halimedineae)	'Ostreobium'			+	
OTU 750	Photot.	Euk.	Chlorophyta	Ulvophyceae	Bryopsidales	'Pseudochlorodesmidaceae' (Halimedineae)	'Pseudochlorodesmis'		+	+	
OTU 136	Photot.	Euk.	Chlorophyta	Ulvophyceae	Bryopsidales	'Pseudochlorodesmidaceae' (Halimedineae)	'Pseudochlorodesmis'		+	+	
OTU 367	Photot.	Euk.	Chlorophyta	Ulvophyceae	Bryopsidales	'Siphonogramenaceae' (Halimedineae)	'Pseudochlorodesmis'	+			+
OTU 486	Photot.	Euk.	Chlorophyta	Ulvophyceae	Bryopsidales	aff. Bryopsidaceae (Bryopsidineae)	--			+	
OTU 375	Photot.	Euk.	Chlorophyta	Ulvophyceae	Bryopsidales	Bryopsidaceae (Bryopsidineae)	Bryopsis		+	+	
OTU 198	Photot.	Euk.	Chlorophyta	Ulvophyceae	Bryopsidales	Bryopsidaceae (Bryopsidineae)	Bryopsis		+	+	
OTU 333	Photot.	Euk.	Chlorophyta	Ulvophyceae	Bryopsidales	Bryopsidaceae (Bryopsidineae)	Bryopsis		+	+	
OTU 368	Photot.	Euk.	Chlorophyta	Ulvophyceae	Bryopsidales	Bryopsidaceae (Bryopsidineae)	Bryopsis		+	+	
OTU 790	Photot.	Euk.	Chlorophyta	Ulvophyceae	Bryopsidales	Bryopsidaceae (Bryopsidineae)	Bryopsis			+	
OTU 087	Photot.	Euk.	Chlorophyta	Ulvophyceae	Bryopsidales	Bryopsidaceae (Bryopsidineae)	Bryopsis			+	
OTU 353	Photot.	Euk.	Chlorophyta	Ulvophyceae	Bryopsidales	Bryopsidaceae (Bryopsidineae)	Bryopsis		+		
OTU 509	Photot.	Euk.	Chlorophyta	Ulvophyceae	Bryopsidales	Caulerpaceae (Halimedineae)	Caulerpa 'ambigua'		+	+	
OTU 213	Photot.	Euk.	Chlorophyta	Ulvophyceae	Bryopsidales	Derbesiaceae (Bryopsidineae)	Derbesia			+	
OTU 170	Photot.	Euk.	Chlorophyta	Ulvophyceae	Bryopsidales	Derbesiaceae (Bryopsidineae)	Derbesia			+	
OTU 268	Photot.	Euk.	Chlorophyta	Ulvophyceae	Bryopsidales	Derbesiaceae (Bryopsidineae)	Derbesia			+	
OTU 436	Photot.	Euk.	Chlorophyta	Ulvophyceae	Bryopsidales	Derbesiaceae (Bryopsidineae)	Derbesia			+	
OTU 154	Photot.	Euk.	Chlorophyta	Ulvophyceae	Bryopsidales	Derbesiaceae (Bryopsidineae)	Derbesia			+	
OTU 323	Photot.	Euk.	Chlorophyta	Ulvophyceae	Bryopsidales	Derbesiaceae (Bryopsidineae)	Derbesia			+	
OTU 144	Photot.	Euk.	Chlorophyta	Ulvophyceae	Bryopsidales	Halimedaceae (Halimedineae)	Halimeda			+	
OTU 338	Photot.	Euk.	Chlorophyta	Ulvophyceae	Bryopsidales	Rhipiliaceae (Halimedineae)	--		+	+	
OTU 654	Photot.	Euk.	Chlorophyta	Ulvophyceae	Bryopsidales	Rhipiliaceae (Halimedineae)	--		+	+	
OTU 334	Photot.	Euk.	Chlorophyta	Ulvophyceae	Bryopsidales	Rhipiliaceae (Halimedineae)	--			+	
OTU 647	Photot.	Euk.	Cryptophyta	Cryptophyceae	--	--	--			+	
OTU 292	Photot.	Euk.	Cryptophyta	Cryptophyceae	--	--	--			+	
OTU 356	Photot.	Euk.	Haptophyta	Coccolithophyceae	Coccolithales	Hymenomonadaceae	Ochrosphaera	+	+	+	+
OTU 609	Photot.	Euk.	Haptophyta	Coccolithophyceae	Coccolithales	Hymenomonadaceae	--			+	
OTU 624	Photot.	Euk.	Haptophyta	Pavlovophyceae	--	--	--		+		
OTU 169	Photot.	Euk.	Haptophyta	Pavlovophyceae	--	--	--		+		
OTU 619	Photot.	Euk.	Ochrophyta	--	--	--	--			+	
OTU 261	Photot.	Euk.	Ochrophyta	--	--	--	--			+	
OTU 143	Photot.	Euk.	Ochrophyta	--	--	--	--			+	
OTU 627	Photot.	Euk.	Ochrophyta	--	--	--	--		+		
OTU 599	Photot.	Euk.	Ochrophyta	--	--	--	--		+		
OTU 145	Photot.	Euk.	Ochrophyta	--	--	--	--		+		
OTU 295	Photot.	Euk.	Ochrophyta	--	--	--	--		+		
OTU 792	Photot.	Euk.	Ochrophyta	'Bacillariophyta'	--	--	--		+	+	
OTU 497	Photot.	Euk.	Ochrophyta	'Bacillariophyta'	--	--	--		+	+	
OTU 518	Photot.	Euk.	Ochrophyta	'Bacillariophyta'	--	--	--		+	+	
OTU 396	Photot.	Euk.	Ochrophyta	'Bacillariophyta'	--	--	--		+	+	

Identifier	Function	Domain	Phyla	Class	Order	Family (Suborder)	Genus	FL01	FL02	JP07	GM14
OTU 575	Photot.	Euk.	Ochrophyta	'Bacillariophyta'	--	--	--		+	+	
OTU 077	Photot.	Euk.	Ochrophyta	'Bacillariophyta'	--	--	--		+	+	
OTU 288	Photot.	Euk.	Ochrophyta	'Bacillariophyta'	--	--	--		+	+	
OTU 763	Photot.	Euk.	Ochrophyta	'Bacillariophyta'	--	--	--		+	+	
OTU 225	Photot.	Euk.	Ochrophyta	'Bacillariophyta'	--	--	--		+	+	
OTU 142	Photot.	Euk.	Ochrophyta	'Bacillariophyta'	--	--	--		+	+	
OTU 067	Photot.	Euk.	Ochrophyta	'Bacillariophyta'	--	--	--	+	+	+	
OTU 038	Photot.	Euk.	Ochrophyta	'Bacillariophyta'	--	--	--		+	+	
OTU 043	Photot.	Euk.	Ochrophyta	'Bacillariophyta'	--	--	--		+	+	
OTU 204	Photot.	Euk.	Ochrophyta	'Bacillariophyta'	--	--	--		+	+	
OTU 452	Photot.	Euk.	Ochrophyta	'Bacillariophyta'	--	--	--		+	+	
OTU 369	Photot.	Euk.	Ochrophyta	'Bacillariophyta'	--	--	--		+	+	
OTU 108	Photot.	Euk.	Ochrophyta	'Bacillariophyta'	--	--	--		+	+	
OTU 102	Photot.	Euk.	Ochrophyta	'Bacillariophyta'	--	--	--		+	+	
OTU 296	Photot.	Euk.	Ochrophyta	'Bacillariophyta'	--	--	--		+	+	
OTU 753	Photot.	Euk.	Ochrophyta	'Bacillariophyta'	--	--	--		+	+	
OTU 441	Photot.	Euk.	Ochrophyta	'Bacillariophyta'	--	--	--		+	+	
OTU 722	Photot.	Euk.	Ochrophyta	'Bacillariophyta'	--	--	--		+	+	
OTU 223	Photot.	Euk.	Ochrophyta	'Bacillariophyta'	--	--	--		+	+	
OTU 234	Photot.	Euk.	Ochrophyta	'Bacillariophyta'	--	--	--		+	+	
OTU 477	Photot.	Euk.	Ochrophyta	'Bacillariophyta'	--	--	--		+	+	
OTU 780	Photot.	Euk.	Ochrophyta	'Bacillariophyta'	--	--	--		+	+	
OTU 026	Photot.	Euk.	Ochrophyta	'Bacillariophyta'	--	--	--		+	+	
OTU 208	Photot.	Euk.	Ochrophyta	'Bacillariophyta'	--	--	--		+	+	
OTU 306	Photot.	Euk.	Ochrophyta	'Bacillariophyta'	--	--	--		+	+	
OTU 422	Photot.	Euk.	Ochrophyta	'Bacillariophyta'	--	--	--		+	+	
OTU 740	Photot.	Euk.	Ochrophyta	'Bacillariophyta'	--	--	--		+	+	
OTU 252	Photot.	Euk.	Ochrophyta	'Bacillariophyta'	--	--	--		+	+	
OTU 487	Photot.	Euk.	Ochrophyta	'Bacillariophyta'	--	--	--		+	+	
OTU 411	Photot.	Euk.	Ochrophyta	'Bacillariophyta'	--	--	--		+	+	
OTU 429	Photot.	Euk.	Ochrophyta	'Bacillariophyta'	--	--	--		+	+	
OTU 522	Photot.	Euk.	Ochrophyta	'Bacillariophyta'	--	--	--		+	+	
OTU 194	Photot.	Euk.	Ochrophyta	'Bacillariophyta'	--	--	--		+	+	
OTU 420	Photot.	Euk.	Ochrophyta	'Bacillariophyta'	--	--	--		+	+	
OTU 521	Photot.	Euk.	Ochrophyta	'Bacillariophyta'	--	--	--		+	+	
OTU 503	Photot.	Euk.	Ochrophyta	'Bacillariophyta'	--	--	--		+	+	
OTU 126	Photot.	Euk.	Ochrophyta	'Bacillariophyta'	--	--	--		+	+	
OTU 203	Photot.	Euk.	Ochrophyta	'Bacillariophyta'	--	--	--			+	
OTU 742	Photot.	Euk.	Ochrophyta	'Bacillariophyta'	--	--	--			+	
OTU 211	Photot.	Euk.	Ochrophyta	'Bacillariophyta'	--	--	--			+	
OTU 346	Photot.	Euk.	Ochrophyta	'Bacillariophyta'	--	--	--			+	
OTU 777	Photot.	Euk.	Ochrophyta	'Bacillariophyta'	--	--	--			+	
OTU 544	Photot.	Euk.	Ochrophyta	'Bacillariophyta'	--	--	--			+	
OTU 210	Photot.	Euk.	Ochrophyta	'Bacillariophyta'	--	--	--			+	
OTU 794	Photot.	Euk.	Ochrophyta	'Bacillariophyta'	--	--	--			+	
OTU 770	Photot.	Euk.	Ochrophyta	'Bacillariophyta'	--	--	--			+	
OTU 746	Photot.	Euk.	Ochrophyta	'Bacillariophyta'	--	--	--			+	
OTU 783	Photot.	Euk.	Ochrophyta	'Bacillariophyta'	--	--	--			+	
OTU 461	Photot.	Euk.	Ochrophyta	'Bacillariophyta'	--	--	--			+	
OTU 180	Photot.	Euk.	Ochrophyta	'Bacillariophyta'	--	--	--			+	
OTU 787	Photot.	Euk.	Ochrophyta	'Bacillariophyta'	--	--	--			+	
OTU 788	Photot.	Euk.	Ochrophyta	'Bacillariophyta'	--	--	--			+	
OTU 246	Photot.	Euk.	Ochrophyta	'Bacillariophyta'	--	--	--			+	
OTU 433	Photot.	Euk.	Ochrophyta	'Bacillariophyta'	--	--	--			+	
OTU 179	Photot.	Euk.	Ochrophyta	'Bacillariophyta'	--	--	--			+	
OTU 397	Photot.	Euk.	Ochrophyta	'Bacillariophyta'	--	--	--			+	
OTU 471	Photot.	Euk.	Ochrophyta	'Bacillariophyta'	--	--	--			+	
OTU 182	Photot.	Euk.	Ochrophyta	'Bacillariophyta'	--	--	--			+	
OTU 674	Photot.	Euk.	Ochrophyta	'Bacillariophyta'	--	--	--			+	
OTU 682	Photot.	Euk.	Ochrophyta	'Bacillariophyta'	--	--	--			+	
OTU 382	Photot.	Euk.	Ochrophyta	'Bacillariophyta'	--	--	--			+	
OTU 025	Photot.	Euk.	Ochrophyta	'Bacillariophyta'	--	--	--			+	
OTU 032	Photot.	Euk.	Ochrophyta	'Bacillariophyta'	--	--	--			+	
OTU 606	Photot.	Euk.	Ochrophyta	'Bacillariophyta'	--	--	--			+	
OTU 036	Photot.	Euk.	Ochrophyta	'Bacillariophyta'	--	--	--			+	
OTU 250	Photot.	Euk.	Ochrophyta	'Bacillariophyta'	--	--	--			+	

Identifier	Function	Domain	Phyla	Class	Order	Family (Suborder)	Genus	FL01	FL02	JP07	GM14
OTU 701	Photot.	Euk.	Ochrophyta	'Bacillariophyta'	--	--	--			+	
OTU 576	Photot.	Euk.	Ochrophyta	'Bacillariophyta'	--	--	--			+	
OTU 708	Photot.	Euk.	Ochrophyta	'Bacillariophyta'	--	--	--			+	
OTU 637	Photot.	Euk.	Ochrophyta	'Bacillariophyta'	--	--	--			+	
OTU 003	Photot.	Euk.	Ochrophyta	'Bacillariophyta'	--	--	--			+	
OTU 090	Photot.	Euk.	Ochrophyta	'Bacillariophyta'	--	--	--			+	
OTU 681	Photot.	Euk.	Ochrophyta	'Bacillariophyta'	--	--	--			+	
OTU 020	Photot.	Euk.	Ochrophyta	'Bacillariophyta'	--	--	--			+	
OTU 226	Photot.	Euk.	Ochrophyta	'Bacillariophyta'	--	--	--			+	
OTU 557	Photot.	Euk.	Ochrophyta	'Bacillariophyta'	--	--	--			+	
OTU 600	Photot.	Euk.	Ochrophyta	'Bacillariophyta'	--	--	--			+	
OTU 691	Photot.	Euk.	Ochrophyta	'Bacillariophyta'	--	--	--			+	
OTU 595	Photot.	Euk.	Ochrophyta	'Bacillariophyta'	--	--	--			+	
OTU 661	Photot.	Euk.	Ochrophyta	'Bacillariophyta'	--	--	--			+	
OTU 148	Photot.	Euk.	Ochrophyta	'Bacillariophyta'	--	--	--			+	
OTU 616	Photot.	Euk.	Ochrophyta	'Bacillariophyta'	--	--	--			+	
OTU 258	Photot.	Euk.	Ochrophyta	'Bacillariophyta'	--	--	--			+	
OTU 720	Photot.	Euk.	Ochrophyta	'Bacillariophyta'	--	--	--			+	
OTU 088	Photot.	Euk.	Ochrophyta	'Bacillariophyta'	--	--	--			+	
OTU 612	Photot.	Euk.	Ochrophyta	'Bacillariophyta'	--	--	--			+	
OTU 590	Photot.	Euk.	Ochrophyta	'Bacillariophyta'	--	--	--			+	
OTU 694	Photot.	Euk.	Ochrophyta	'Bacillariophyta'	--	--	--			+	
OTU 059	Photot.	Euk.	Ochrophyta	'Bacillariophyta'	--	--	--			+	
OTU 222	Photot.	Euk.	Ochrophyta	'Bacillariophyta'	--	--	--			+	
OTU 239	Photot.	Euk.	Ochrophyta	'Bacillariophyta'	--	--	--			+	
OTU 146	Photot.	Euk.	Ochrophyta	'Bacillariophyta'	--	--	--			+	
OTU 316	Photot.	Euk.	Ochrophyta	'Bacillariophyta'	--	--	--			+	
OTU 554	Photot.	Euk.	Ochrophyta	'Bacillariophyta'	--	--	--			+	
OTU 244	Photot.	Euk.	Ochrophyta	'Bacillariophyta'	--	--	--			+	
OTU 277	Photot.	Euk.	Ochrophyta	'Bacillariophyta'	--	--	--			+	
OTU 276	Photot.	Euk.	Ochrophyta	'Bacillariophyta'	--	--	--			+	
OTU 095	Photot.	Euk.	Ochrophyta	'Bacillariophyta'	--	--	--			+	
OTU 141	Photot.	Euk.	Ochrophyta	'Bacillariophyta'	--	--	--			+	
OTU 282	Photot.	Euk.	Ochrophyta	'Bacillariophyta'	--	--	--			+	
OTU 565	Photot.	Euk.	Ochrophyta	'Bacillariophyta'	--	--	--			+	
OTU 550	Photot.	Euk.	Ochrophyta	'Bacillariophyta'	--	--	--			+	
OTU 611	Photot.	Euk.	Ochrophyta	'Bacillariophyta'	--	--	--			+	
OTU 274	Photot.	Euk.	Ochrophyta	'Bacillariophyta'	--	--	--			+	
OTU 559	Photot.	Euk.	Ochrophyta	'Bacillariophyta'	--	--	--			+	
OTU 285	Photot.	Euk.	Ochrophyta	'Bacillariophyta'	--	--	--			+	
OTU 317	Photot.	Euk.	Ochrophyta	'Bacillariophyta'	--	--	--			+	
OTU 071	Photot.	Euk.	Ochrophyta	'Bacillariophyta'	--	--	--			+	
OTU 279	Photot.	Euk.	Ochrophyta	'Bacillariophyta'	--	--	--			+	
OTU 634	Photot.	Euk.	Ochrophyta	'Bacillariophyta'	--	--	--			+	
OTU 725	Photot.	Euk.	Ochrophyta	'Bacillariophyta'	--	--	--	+	+		
OTU 362	Photot.	Euk.	Ochrophyta	'Bacillariophyta'	--	--	--		+		
OTU 704	Photot.	Euk.	Ochrophyta	'Bacillariophyta'	--	--	--		+		
OTU 153	Photot.	Euk.	Ochrophyta	'Bacillariophyta'	--	--	--		+		
OTU 628	Photot.	Euk.	Ochrophyta	'Bacillariophyta'	--	--	--		+		
OTU 116	Photot.	Euk.	Ochrophyta	'Bacillariophyta'	--	--	--		+		
OTU 147	Photot.	Euk.	Ochrophyta	'Bacillariophyta'	--	--	--		+		
OTU 118	Photot.	Euk.	Ochrophyta	'Bacillariophyta'	--	--	--		+		
OTU 631	Photot.	Euk.	Ochrophyta	'Bacillariophyta'	--	--	--		+		
OTU 218	Photot.	Euk.	Ochrophyta	'Bacillariophyta'	--	--	--		+		
OTU 610	Photot.	Euk.	Ochrophyta	'Bacillariophyta'	--	--	--		+		
OTU 152	Photot.	Euk.	Ochrophyta	'Bacillariophyta'	--	--	--		+		
OTU 633	Photot.	Euk.	Ochrophyta	'Bacillariophyta'	--	--	--		+		
OTU 104	Photot.	Euk.	Ochrophyta	'Bacillariophyta'	--	--	--		+		
OTU 711	Photot.	Euk.	Ochrophyta	'Bacillariophyta'	--	--	--		+		
OTU 004	Photot.	Euk.	Ochrophyta	'Bacillariophyta'	--	--	--		+		
OTU 325	Photot.	Euk.	Ochrophyta	'Bacillariophyta'	--	--	--		+		
OTU 561	Photot.	Euk.	Ochrophyta	'Bacillariophyta'	--	--	--		+		
OTU 656	Photot.	Euk.	Ochrophyta	'Bacillariophyta'	--	--	--		+		
OTU 564	Photot.	Euk.	Ochrophyta	'Bacillariophyta'	--	--	--		+		
OTU 015	Photot.	Euk.	Ochrophyta	'Bacillariophyta'	--	--	--		+		
OTU 247	Photot.	Euk.	Ochrophyta	'Bacillariophyta'	--	--	--		+		

Identifier	Function	Domain	Phyla	Class	Order	Family (Suborder)	Genus	FL01	FL02	JP07	GM14
OTU 219	Photot.	Euk.	Ochrophyta	'Bacillariophyta'	--	--	--		+		
OTU 689	Photot.	Euk.	Ochrophyta	'Bacillariophyta'	--	--	--		+		
OTU 673	Photot.	Euk.	Ochrophyta	'Bacillariophyta'	--	--	--		+		
OTU 029	Photot.	Euk.	Ochrophyta	'Bacillariophyta'	--	--	--		+		
OTU 669	Photot.	Euk.	Ochrophyta	'Bacillariophyta'	--	--	--		+		
OTU 714	Photot.	Euk.	Ochrophyta	'Bacillariophyta'	--	--	--		+		
OTU 468	Photot.	Euk.	Ochrophyta	'Bacillariophyta'	--	--	--		+		
OTU 463	Photot.	Euk.	Ochrophyta	'Bacillariophyta'	--	--	--		+		
OTU 593	Photot.	Euk.	Ochrophyta	'Bacillariophyta'	--	--	--		+		
OTU 014	Photot.	Euk.	Ochrophyta	'Bacillariophyta'	--	--	--		+		
OTU 047	Photot.	Euk.	Ochrophyta	'Bacillariophyta'	--	--	--		+		
OTU 255	Photot.	Euk.	Ochrophyta	'Bacillariophyta'	--	--	--		+		
OTU 789	Photot.	Euk.	Ochrophyta	'Bacillariophyta'	--	--	--		+		
OTU 670	Photot.	Euk.	Ochrophyta	'Bacillariophyta'	--	--	--		+		
OTU 253	Photot.	Euk.	Ochrophyta	'Bacillariophyta'	--	--	--		+		
OTU 392	Photot.	Euk.	Ochrophyta	'Bacillariophyta'	--	--	--		+		
OTU 444	Photot.	Euk.	Ochrophyta	'Bacillariophyta'	--	--	--		+		
OTU 051	Photot.	Euk.	Ochrophyta	'Bacillariophyta'	--	--	--		+		
OTU 457	Photot.	Euk.	Ochrophyta	'Bacillariophyta'	--	--	--		+		
OTU 259	Photot.	Euk.	Ochrophyta	'Bacillariophyta'	--	--	--		+		
OTU 445	Photot.	Euk.	Ochrophyta	'Bacillariophyta'	--	--	--		+		
OTU 798	Photot.	Euk.	Ochrophyta	'Bacillariophyta'	--	--	--		+		
OTU 414	Photot.	Euk.	Ochrophyta	'Bacillariophyta'	--	--	--		+		
OTU 177	Photot.	Euk.	Ochrophyta	'Bacillariophyta'	--	--	--		+		
OTU 759	Photot.	Euk.	Ochrophyta	'Bacillariophyta'	--	--	--		+		
OTU 540	Photot.	Euk.	Ochrophyta	'Bacillariophyta'	--	--	--		+		
OTU 417	Photot.	Euk.	Ochrophyta	'Bacillariophyta'	--	--	--		+		
OTU 150	Photot.	Euk.	Ochrophyta	Pelagophyceae	--	--	--		+	+	
OTU 578	Photot.	Euk.	Ochrophyta	Pelagophyceae	--	--	--		+	+	
OTU 197	Photot.	Euk.	Ochrophyta	Pelagophyceae	--	--	--		+	+	
OTU 523	Photot.	Euk.	Ochrophyta	Pelagophyceae	--	--	--			+	
OTU 697	Photot.	Euk.	Ochrophyta	Pelagophyceae	--	--	--			+	
OTU 596	Photot.	Euk.	Ochrophyta	Pelagophyceae	--	--	--			+	
OTU 589	Photot.	Euk.	Ochrophyta	Pelagophyceae	--	--	--			+	
OTU 570	Photot.	Euk.	Ochrophyta	Pelagophyceae	--	--	--			+	
OTU 242	Photot.	Euk.	Ochrophyta	Pelagophyceae	--	--	--		+		
OTU 254	Photot.	Euk.	Ochrophyta	Pelagophyceae	--	--	--		+		
OTU 405	Photot.	Euk.	Ochrophyta	Pelagophyceae	--	--	--		+		
OTU 519	Photot.	Euk.	Ochrophyta	Phaeophyceae	--	--	--		+	+	
OTU 336	Photot.	Euk.	Ochrophyta	Phaeophyceae	--	--	--			+	
OTU 327	Photot.	Euk.	Ochrophyta	Phaeophyceae	--	--	--			+	
OTU 351	Photot.	Euk.	Ochrophyta	Phaeophyceae	--	--	--			+	
OTU 690	Photot.	Euk.	Ochrophyta	Phaeophyceae	--	--	--			+	
OTU 729	Photot.	Euk.	Ochrophyta	Phaeophyceae	--	--	--			+	
OTU 605	Photot.	Euk.	Ochrophyta	Phaeophyceae	--	--	--			+	
OTU 724	Photot.	Euk.	Ochrophyta	Phaeophyceae	--	--	--			+	
OTU 100	Photot.	Euk.	Ochrophyta	Phaeophyceae	--	--	--		+		
OTU 044	Photot.	Euk.	Ochrophyta	Phaeophyceae	Ectocarpales	Ectocarpaceae	--			+	
OTU 140	Photot.	Euk.	Ochrophyta	Phaeophyceae	Ectocarpales	Ectocarpaceae	--		+		
OTU 187	Photot.	Euk.	Ochrophyta	Phaeophyceae	Ectocarpales	Ectocarpaceae	--		+		
OTU 737	Photot.	Euk.	Rhodophyta	--	--	--	--				+
OTU 345	Photot.	Euk.	Rhodophyta	--	--	--	--			+	
OTU 743	Photot.	Euk.	Rhodophyta	Bangiophyceae	Bangiales	Bangiaceae	--			+	
OTU 256	Photot.	Euk.	Rhodophyta	Compsopogonophyceae	--	--	--		+		
OTU 391	Photot.	Euk.	Rhodophyta	Florideophyceae	--	--	--	+			
OTU 439	Photot.	Euk.	Rhodophyta	Florideophyceae	--	--	--	+			
OTU 034	Photot.	Euk.	Rhodophyta	Florideophyceae	--	--	--				+
OTU 200	Photot.	Euk.	Rhodophyta	Florideophyceae	--	--	--			+	
OTU 525	Photot.	Euk.	Rhodophyta	Florideophyceae	--	--	--			+	
OTU 119	Photot.	Euk.	Rhodophyta	Florideophyceae	--	--	--			+	
OTU 235	Photot.	Euk.	Rhodophyta	Florideophyceae	--	--	--		+		
OTU 357	Photot.	Euk.	Rhodophyta	Florideophyceae	'Corallinales-Hapalidiales'	'Corallinaceae-Hapalidiaceae'	--		+	+	
OTU 511	Photot.	Euk.	Rhodophyta	Florideophyceae	'Corallinales-Hapalidiales'	'Corallinaceae-Hapalidiaceae'	--	+	+	+	
OTU 495	Photot.	Euk.	Rhodophyta	Florideophyceae	'Corallinales-Hapalidiales'	'Corallinaceae-Hapalidiaceae'	--		+	+	
OTU 359	Photot.	Euk.	Rhodophyta	Florideophyceae	'Corallinales-Hapalidiales'	'Corallinaceae-Hapalidiaceae'	--		+	+	
OTU 131	Photot.	Euk.	Rhodophyta	Florideophyceae	'Corallinales-Hapalidiales'	'Corallinaceae-Hapalidiaceae'	--		+	+	

Identifier	Function	Domain	Phyla	Class	Order	Family (Suborder)	Genus	FL01	FL02	JP07	GM14
OTU 355	Photot.	Euk.	Rhodophyta	Florideophyceae	'Corallinales-Hapalidiales'	'Corallinaceae-Hapalidiaceae'	--		+	+	
OTU 749	Photot.	Euk.	Rhodophyta	Florideophyceae	'Corallinales-Hapalidiales'	'Corallinaceae-Hapalidiaceae'	--	+		+	
OTU 514	Photot.	Euk.	Rhodophyta	Florideophyceae	'Corallinales-Hapalidiales'	'Corallinaceae-Hapalidiaceae'	--			+	
OTU 216	Photot.	Euk.	Rhodophyta	Florideophyceae	'Corallinales-Hapalidiales'	'Corallinaceae-Hapalidiaceae'	--			+	
OTU 341	Photot.	Euk.	Rhodophyta	Florideophyceae	'Corallinales-Hapalidiales'	'Corallinaceae-Hapalidiaceae'	--			+	
OTU 419	Photot.	Euk.	Rhodophyta	Florideophyceae	'Corallinales-Hapalidiales'	'Corallinaceae-Hapalidiaceae'	--			+	
OTU 744	Photot.	Euk.	Rhodophyta	Florideophyceae	'Corallinales-Hapalidiales'	'Corallinaceae-Hapalidiaceae'	--			+	
OTU 041	Photot.	Euk.	Rhodophyta	Florideophyceae	'Corallinales-Hapalidiales'	'Corallinaceae-Hapalidiaceae'	--			+	
OTU 021	Photot.	Euk.	Rhodophyta	Florideophyceae	'Corallinales-Hapalidiales'	'Corallinaceae-Hapalidiaceae'	--			+	
OTU 039	Photot.	Euk.	Rhodophyta	Florideophyceae	'Corallinales-Hapalidiales'	'Corallinaceae-Hapalidiaceae'	--		+		
OTU 739	Photot.	Euk.	Rhodophyta	Florideophyceae	'Corallinales-Hapalidiales'	'Corallinaceae-Hapalidiaceae'	--		+		
OTU 178	Photot.	Euk.	Rhodophyta	Florideophyceae	'Corallinales-Hapalidiales'	Corallinaceae	--		+		
OTU 191	Photot.	Euk.	Rhodophyta	Florideophyceae	Ceramiales	--	--			+	
OTU 472	Photot.	Euk.	Rhodophyta	Florideophyceae	Ceramiales	--	--			+	
OTU 542	Photot.	Euk.	Rhodophyta	Florideophyceae	Gigartinales' s. lato	Dumontiaceae	--		+		
OTU 380	Photot.	Euk.	Rhodophyta	Florideophyceae	Gigartinales' s. lato	Rhizophyllidaceae	Rhizophyllis	+			+
OTU 215	Photot.	Euk.	Rhodophyta	Florideophyceae	Peyssonneliales	Peyssonneliaceae	--			+	
OTU 646	Photot.	Euk.	Rhodophyta	Florideophyceae	Peyssonneliales	Peyssonneliaceae	--			+	
OTU 149	Photot.	Euk.	Rhodophyta	Florideophyceae	Peyssonneliales	Peyssonneliaceae	--			+	
OTU 407	Photot.	Euk.	Rhodophyta	Florideophyceae	Peyssonneliales	Peyssonneliaceae	--		+		
OTU 556	Photot.	Euk.	Rhodophyta	Florideophyceae	Peyssonneliales	Peyssonneliaceae	Riquetophycus		+		
OTU 374	Photot.	Euk.	Rhodophyta	Stylonematophyceae	Stylonematales	Stylonemataceae	--		+	+	
OTU 190	Photot.	Prok.	Cyanophyta	Cyanophyceae	--	--	--		+	+	
OTU 009	Photot.	Prok.	Cyanophyta	Cyanophyceae	--	--	--		+	+	
OTU 453	Photot.	Prok.	Cyanophyta	Cyanophyceae	--	--	--		+	+	
OTU 128	Photot.	Prok.	Cyanophyta	Cyanophyceae	--	--	--		+	+	
OTU 201	Photot.	Prok.	Cyanophyta	Cyanophyceae	--	--	--		+	+	
OTU 594	Photot.	Prok.	Cyanophyta	Cyanophyceae	--	--	--		+	+	
OTU 373	Photot.	Prok.	Cyanophyta	Cyanophyceae	--	--	--		+	+	
OTU 130	Photot.	Prok.	Cyanophyta	Cyanophyceae	--	--	--		+	+	
OTU 052	Photot.	Prok.	Cyanophyta	Cyanophyceae	--	--	--		+	+	
OTU 082	Photot.	Prok.	Cyanophyta	Cyanophyceae	--	--	--		+	+	
OTU 189	Photot.	Prok.	Cyanophyta	Cyanophyceae	--	--	--		+	+	
OTU 537	Photot.	Prok.	Cyanophyta	Cyanophyceae	--	--	--		+	+	
OTU 313	Photot.	Prok.	Cyanophyta	Cyanophyceae	--	--	--		+	+	
OTU 651	Photot.	Prok.	Cyanophyta	Cyanophyceae	--	--	--		+	+	
OTU 723	Photot.	Prok.	Cyanophyta	Cyanophyceae	--	--	--		+	+	
OTU 348	Photot.	Prok.	Cyanophyta	Cyanophyceae	--	--	--		+	+	
OTU 205	Photot.	Prok.	Cyanophyta	Cyanophyceae	--	--	--		+	+	
OTU 481	Photot.	Prok.	Cyanophyta	Cyanophyceae	--	--	--		+	+	
OTU 707	Photot.	Prok.	Cyanophyta	Cyanophyceae	--	--	--		+	+	
OTU 683	Photot.	Prok.	Cyanophyta	Cyanophyceae	--	--	--		+	+	
OTU 352	Photot.	Prok.	Cyanophyta	Cyanophyceae	--	--	--		+	+	
OTU 793	Photot.	Prok.	Cyanophyta	Cyanophyceae	--	--	--		+	+	
OTU 693	Photot.	Prok.	Cyanophyta	Cyanophyceae	--	--	--		+	+	
OTU 796	Photot.	Prok.	Cyanophyta	Cyanophyceae	--	--	--		+	+	
OTU 524	Photot.	Prok.	Cyanophyta	Cyanophyceae	--	--	--		+	+	
OTU 771	Photot.	Prok.	Cyanophyta	Cyanophyceae	--	--	--		+	+	
OTU 752	Photot.	Prok.	Cyanophyta	Cyanophyceae	--	--	--		+	+	
OTU 526	Photot.	Prok.	Cyanophyta	Cyanophyceae	--	--	--		+	+	
OTU 349	Photot.	Prok.	Cyanophyta	Cyanophyceae	--	--	--		+	+	
OTU 531	Photot.	Prok.	Cyanophyta	Cyanophyceae	--	--	--		+	+	
OTU 735	Photot.	Prok.	Cyanophyta	Cyanophyceae	--	--	--		+	+	
OTU 501	Photot.	Prok.	Cyanophyta	Cyanophyceae	--	--	--		+	+	
OTU 193	Photot.	Prok.	Cyanophyta	Cyanophyceae	--	--	--		+	+	
OTU 339	Photot.	Prok.	Cyanophyta	Cyanophyceae	--	--	--		+	+	
OTU 337	Photot.	Prok.	Cyanophyta	Cyanophyceae	--	--	--		+	+	
OTU 378	Photot.	Prok.	Cyanophyta	Cyanophyceae	--	--	--		+	+	
OTU 372	Photot.	Prok.	Cyanophyta	Cyanophyceae	--	--	--		+	+	
OTU 379	Photot.	Prok.	Cyanophyta	Cyanophyceae	--	--	--		+	+	
OTU 504	Photot.	Prok.	Cyanophyta	Cyanophyceae	--	--	--		+	+	
OTU 801	Photot.	Prok.	Cyanophyta	Cyanophyceae	--	--	--		+	+	
OTU 358	Photot.	Prok.	Cyanophyta	Cyanophyceae	--	--	--		+	+	
OTU 155	Photot.	Prok.	Cyanophyta	Cyanophyceae	--	--	--	+		+	
OTU 404	Photot.	Prok.	Cyanophyta	Cyanophyceae	--	--	--	+		+	
OTU 717	Photot.	Prok.	Cyanophyta	Cyanophyceae	--	--	--	+		+	

Identifier	Function	Domain	Phyla	Class	Order	Family (Suborder)	Genus	FL01	FL02	JP07	GM14
OTU 733	Photot.	Prok.	Cyanophyta	Cyanophyceae	--	--	--	+			
OTU 494	Photot.	Prok.	Cyanophyta	Cyanophyceae	--	--	--			+	
OTU 332	Photot.	Prok.	Cyanophyta	Cyanophyceae	--	--	--			+	
OTU 748	Photot.	Prok.	Cyanophyta	Cyanophyceae	--	--	--			+	
OTU 331	Photot.	Prok.	Cyanophyta	Cyanophyceae	--	--	--			+	
OTU 498	Photot.	Prok.	Cyanophyta	Cyanophyceae	--	--	--			+	
OTU 196	Photot.	Prok.	Cyanophyta	Cyanophyceae	--	--	--			+	
OTU 328	Photot.	Prok.	Cyanophyta	Cyanophyceae	--	--	--			+	
OTU 536	Photot.	Prok.	Cyanophyta	Cyanophyceae	--	--	--			+	
OTU 535	Photot.	Prok.	Cyanophyta	Cyanophyceae	--	--	--			+	
OTU 786	Photot.	Prok.	Cyanophyta	Cyanophyceae	--	--	--			+	
OTU 776	Photot.	Prok.	Cyanophyta	Cyanophyceae	--	--	--			+	
OTU 207	Photot.	Prok.	Cyanophyta	Cyanophyceae	--	--	--			+	
OTU 151	Photot.	Prok.	Cyanophyta	Cyanophyceae	--	--	--			+	
OTU 412	Photot.	Prok.	Cyanophyta	Cyanophyceae	--	--	--			+	
OTU 534	Photot.	Prok.	Cyanophyta	Cyanophyceae	--	--	--			+	
OTU 428	Photot.	Prok.	Cyanophyta	Cyanophyceae	--	--	--			+	
OTU 217	Photot.	Prok.	Cyanophyta	Cyanophyceae	--	--	--			+	
OTU 343	Photot.	Prok.	Cyanophyta	Cyanophyceae	--	--	--			+	
OTU 520	Photot.	Prok.	Cyanophyta	Cyanophyceae	--	--	--			+	
OTU 747	Photot.	Prok.	Cyanophyta	Cyanophyceae	--	--	--			+	
OTU 528	Photot.	Prok.	Cyanophyta	Cyanophyceae	--	--	--			+	
OTU 764	Photot.	Prok.	Cyanophyta	Cyanophyceae	--	--	--			+	
OTU 762	Photot.	Prok.	Cyanophyta	Cyanophyceae	--	--	--			+	
OTU 173	Photot.	Prok.	Cyanophyta	Cyanophyceae	--	--	--			+	
OTU 167	Photot.	Prok.	Cyanophyta	Cyanophyceae	--	--	--			+	
OTU 541	Photot.	Prok.	Cyanophyta	Cyanophyceae	--	--	--			+	
OTU 761	Photot.	Prok.	Cyanophyta	Cyanophyceae	--	--	--			+	
OTU 758	Photot.	Prok.	Cyanophyta	Cyanophyceae	--	--	--			+	
OTU 755	Photot.	Prok.	Cyanophyta	Cyanophyceae	--	--	--			+	
OTU 381	Photot.	Prok.	Cyanophyta	Cyanophyceae	--	--	--			+	
OTU 174	Photot.	Prok.	Cyanophyta	Cyanophyceae	--	--	--			+	
OTU 462	Photot.	Prok.	Cyanophyta	Cyanophyceae	--	--	--			+	
OTU 176	Photot.	Prok.	Cyanophyta	Cyanophyceae	--	--	--			+	
OTU 760	Photot.	Prok.	Cyanophyta	Cyanophyceae	--	--	--			+	
OTU 172	Photot.	Prok.	Cyanophyta	Cyanophyceae	--	--	--			+	
OTU 408	Photot.	Prok.	Cyanophyta	Cyanophyceae	--	--	--			+	
OTU 175	Photot.	Prok.	Cyanophyta	Cyanophyceae	--	--	--			+	
OTU 421	Photot.	Prok.	Cyanophyta	Cyanophyceae	--	--	--			+	
OTU 791	Photot.	Prok.	Cyanophyta	Cyanophyceae	--	--	--			+	
OTU 802	Photot.	Prok.	Cyanophyta	Cyanophyceae	--	--	--			+	
OTU 168	Photot.	Prok.	Cyanophyta	Cyanophyceae	--	--	--			+	
OTU 402	Photot.	Prok.	Cyanophyta	Cyanophyceae	--	--	--			+	
OTU 413	Photot.	Prok.	Cyanophyta	Cyanophyceae	--	--	--			+	
OTU 775	Photot.	Prok.	Cyanophyta	Cyanophyceae	--	--	--			+	
OTU 383	Photot.	Prok.	Cyanophyta	Cyanophyceae	--	--	--			+	
OTU 425	Photot.	Prok.	Cyanophyta	Cyanophyceae	--	--	--			+	
OTU 483	Photot.	Prok.	Cyanophyta	Cyanophyceae	--	--	--			+	
OTU 426	Photot.	Prok.	Cyanophyta	Cyanophyceae	--	--	--			+	
OTU 432	Photot.	Prok.	Cyanophyta	Cyanophyceae	--	--	--			+	
OTU 489	Photot.	Prok.	Cyanophyta	Cyanophyceae	--	--	--			+	
OTU 430	Photot.	Prok.	Cyanophyta	Cyanophyceae	--	--	--			+	
OTU 326	Photot.	Prok.	Cyanophyta	Cyanophyceae	--	--	--			+	
OTU 530	Photot.	Prok.	Cyanophyta	Cyanophyceae	--	--	--			+	
OTU 803	Photot.	Prok.	Cyanophyta	Cyanophyceae	--	--	--			+	
OTU 484	Photot.	Prok.	Cyanophyta	Cyanophyceae	--	--	--			+	
OTU 437	Photot.	Prok.	Cyanophyta	Cyanophyceae	--	--	--			+	
OTU 403	Photot.	Prok.	Cyanophyta	Cyanophyceae	--	--	--			+	
OTU 431	Photot.	Prok.	Cyanophyta	Cyanophyceae	--	--	--			+	
OTU 774	Photot.	Prok.	Cyanophyta	Cyanophyceae	--	--	--			+	
OTU 438	Photot.	Prok.	Cyanophyta	Cyanophyceae	--	--	--			+	
OTU 773	Photot.	Prok.	Cyanophyta	Cyanophyceae	--	--	--			+	
OTU 394	Photot.	Prok.	Cyanophyta	Cyanophyceae	--	--	--			+	
OTU 424	Photot.	Prok.	Cyanophyta	Cyanophyceae	--	--	--			+	
OTU 620	Photot.	Prok.	Cyanophyta	Cyanophyceae	--	--	--			+	
OTU 399	Photot.	Prok.	Cyanophyta	Cyanophyceae	--	--	--			+	

Identifier	Function	Domain	Phyla	Class	Order	Family (Suborder)	Genus	FL01	FL02	JP07	GM14
OTU 267	Photot.	Prok.	Cyanophyta	Cyanophyceae	--	--	--			+	
OTU 732	Photot.	Prok.	Cyanophyta	Cyanophyceae	--	--	--			+	
OTU 181	Photot.	Prok.	Cyanophyta	Cyanophyceae	--	--	--			+	
OTU 582	Photot.	Prok.	Cyanophyta	Cyanophyceae	--	--	--			+	
OTU 049	Photot.	Prok.	Cyanophyta	Cyanophyceae	--	--	--			+	
OTU 679	Photot.	Prok.	Cyanophyta	Cyanophyceae	--	--	--			+	
OTU 680	Photot.	Prok.	Cyanophyta	Cyanophyceae	--	--	--			+	
OTU 037	Photot.	Prok.	Cyanophyta	Cyanophyceae	--	--	--			+	
OTU 027	Photot.	Prok.	Cyanophyta	Cyanophyceae	--	--	--			+	
OTU 591	Photot.	Prok.	Cyanophyta	Cyanophyceae	--	--	--			+	
OTU 390	Photot.	Prok.	Cyanophyta	Cyanophyceae	--	--	--			+	
OTU 470	Photot.	Prok.	Cyanophyta	Cyanophyceae	--	--	--			+	
OTU 050	Photot.	Prok.	Cyanophyta	Cyanophyceae	--	--	--			+	
OTU 466	Photot.	Prok.	Cyanophyta	Cyanophyceae	--	--	--			+	
OTU 618	Photot.	Prok.	Cyanophyta	Cyanophyceae	--	--	--			+	
OTU 678	Photot.	Prok.	Cyanophyta	Cyanophyceae	--	--	--			+	
OTU 685	Photot.	Prok.	Cyanophyta	Cyanophyceae	--	--	--			+	
OTU 031	Photot.	Prok.	Cyanophyta	Cyanophyceae	--	--	--			+	
OTU 667	Photot.	Prok.	Cyanophyta	Cyanophyceae	--	--	--			+	
OTU 401	Photot.	Prok.	Cyanophyta	Cyanophyceae	--	--	--			+	
OTU 030	Photot.	Prok.	Cyanophyta	Cyanophyceae	--	--	--			+	
OTU 270	Photot.	Prok.	Cyanophyta	Cyanophyceae	--	--	--			+	
OTU 684	Photot.	Prok.	Cyanophyta	Cyanophyceae	--	--	--			+	
OTU 571	Photot.	Prok.	Cyanophyta	Cyanophyceae	--	--	--			+	
OTU 046	Photot.	Prok.	Cyanophyta	Cyanophyceae	--	--	--			+	
OTU 675	Photot.	Prok.	Cyanophyta	Cyanophyceae	--	--	--			+	
OTU 687	Photot.	Prok.	Cyanophyta	Cyanophyceae	--	--	--			+	
OTU 716	Photot.	Prok.	Cyanophyta	Cyanophyceae	--	--	--			+	
OTU 299	Photot.	Prok.	Cyanophyta	Cyanophyceae	--	--	--			+	
OTU 311	Photot.	Prok.	Cyanophyta	Cyanophyceae	--	--	--			+	
OTU 474	Photot.	Prok.	Cyanophyta	Cyanophyceae	--	--	--			+	
OTU 721	Photot.	Prok.	Cyanophyta	Cyanophyceae	--	--	--			+	
OTU 231	Photot.	Prok.	Cyanophyta	Cyanophyceae	--	--	--			+	
OTU 655	Photot.	Prok.	Cyanophyta	Cyanophyceae	--	--	--			+	
OTU 023	Photot.	Prok.	Cyanophyta	Cyanophyceae	--	--	--			+	
OTU 045	Photot.	Prok.	Cyanophyta	Cyanophyceae	--	--	--			+	
OTU 106	Photot.	Prok.	Cyanophyta	Cyanophyceae	--	--	--			+	
OTU 626	Photot.	Prok.	Cyanophyta	Cyanophyceae	--	--	--			+	
OTU 098	Photot.	Prok.	Cyanophyta	Cyanophyceae	--	--	--			+	
OTU 547	Photot.	Prok.	Cyanophyta	Cyanophyceae	--	--	--			+	
OTU 635	Photot.	Prok.	Cyanophyta	Cyanophyceae	--	--	--			+	
OTU 728	Photot.	Prok.	Cyanophyta	Cyanophyceae	--	--	--			+	
OTU 220	Photot.	Prok.	Cyanophyta	Cyanophyceae	--	--	--			+	
OTU 266	Photot.	Prok.	Cyanophyta	Cyanophyceae	--	--	--			+	
OTU 572	Photot.	Prok.	Cyanophyta	Cyanophyceae	--	--	--			+	
OTU 603	Photot.	Prok.	Cyanophyta	Cyanophyceae	--	--	--			+	
OTU 602	Photot.	Prok.	Cyanophyta	Cyanophyceae	--	--	--			+	
OTU 585	Photot.	Prok.	Cyanophyta	Cyanophyceae	--	--	--			+	
OTU 668	Photot.	Prok.	Cyanophyta	Cyanophyceae	--	--	--			+	
OTU 715	Photot.	Prok.	Cyanophyta	Cyanophyceae	--	--	--			+	
OTU 314	Photot.	Prok.	Cyanophyta	Cyanophyceae	--	--	--			+	
OTU 597	Photot.	Prok.	Cyanophyta	Cyanophyceae	--	--	--			+	
OTU 598	Photot.	Prok.	Cyanophyta	Cyanophyceae	--	--	--			+	
OTU 666	Photot.	Prok.	Cyanophyta	Cyanophyceae	--	--	--			+	
OTU 097	Photot.	Prok.	Cyanophyta	Cyanophyceae	--	--	--			+	
OTU 320	Photot.	Prok.	Cyanophyta	Cyanophyceae	--	--	--			+	
OTU 228	Photot.	Prok.	Cyanophyta	Cyanophyceae	--	--	--			+	
OTU 287	Photot.	Prok.	Cyanophyta	Cyanophyceae	--	--	--			+	
OTU 581	Photot.	Prok.	Cyanophyta	Cyanophyceae	--	--	--			+	
OTU 018	Photot.	Prok.	Cyanophyta	Cyanophyceae	--	--	--			+	
OTU 092	Photot.	Prok.	Cyanophyta	Cyanophyceae	--	--	--			+	
OTU 552	Photot.	Prok.	Cyanophyta	Cyanophyceae	--	--	--			+	
OTU 672	Photot.	Prok.	Cyanophyta	Cyanophyceae	--	--	--			+	
OTU 709	Photot.	Prok.	Cyanophyta	Cyanophyceae	--	--	--			+	
OTU 730	Photot.	Prok.	Cyanophyta	Cyanophyceae	--	--	--			+	
OTU 315	Photot.	Prok.	Cyanophyta	Cyanophyceae	--	--	--			+	

Identifier	Function	Domain	Phyla	Class	Order	Family (Suborder)	Genus	FL01	FL02	JP07	GM14
OTU 555	Photot.	Prok.	Cyanophyta	Cyanophyceae	--	--	--			+	
OTU 091	Photot.	Prok.	Cyanophyta	Cyanophyceae	--	--	--			+	
OTU 300	Photot.	Prok.	Cyanophyta	Cyanophyceae	--	--	--			+	
OTU 567	Photot.	Prok.	Cyanophyta	Cyanophyceae	--	--	--			+	
OTU 621	Photot.	Prok.	Cyanophyta	Cyanophyceae	--	--	--			+	
OTU 063	Photot.	Prok.	Cyanophyta	Cyanophyceae	--	--	--			+	
OTU 017	Photot.	Prok.	Cyanophyta	Cyanophyceae	--	--	--			+	
OTU 283	Photot.	Prok.	Cyanophyta	Cyanophyceae	--	--	--			+	
OTU 286	Photot.	Prok.	Cyanophyta	Cyanophyceae	--	--	--			+	
OTU 545	Photot.	Prok.	Cyanophyta	Cyanophyceae	--	--	--			+	
OTU 546	Photot.	Prok.	Cyanophyta	Cyanophyceae	--	--	--			+	
OTU 588	Photot.	Prok.	Cyanophyta	Cyanophyceae	--	--	--			+	
OTU 237	Photot.	Prok.	Cyanophyta	Cyanophyceae	--	--	--			+	
OTU 284	Photot.	Prok.	Cyanophyta	Cyanophyceae	--	--	--			+	
OTU 592	Photot.	Prok.	Cyanophyta	Cyanophyceae	--	--	--			+	
OTU 710	Photot.	Prok.	Cyanophyta	Cyanophyceae	--	--	--			+	
OTU 551	Photot.	Prok.	Cyanophyta	Cyanophyceae	--	--	--			+	
OTU 307	Photot.	Prok.	Cyanophyta	Cyanophyceae	--	--	--			+	
OTU 120	Photot.	Prok.	Cyanophyta	Cyanophyceae	--	--	--			+	
OTU 304	Photot.	Prok.	Cyanophyta	Cyanophyceae	--	--	--			+	
OTU 641	Photot.	Prok.	Cyanophyta	Cyanophyceae	--	--	--			+	
OTU 066	Photot.	Prok.	Cyanophyta	Cyanophyceae	--	--	--			+	
OTU 073	Photot.	Prok.	Cyanophyta	Cyanophyceae	--	--	--			+	
OTU 138	Photot.	Prok.	Cyanophyta	Cyanophyceae	--	--	--			+	
OTU 161	Photot.	Prok.	Cyanophyta	Cyanophyceae	--	--	--			+	
OTU 275	Photot.	Prok.	Cyanophyta	Cyanophyceae	--	--	--			+	
OTU 566	Photot.	Prok.	Cyanophyta	Cyanophyceae	--	--	--			+	
OTU 696	Photot.	Prok.	Cyanophyta	Cyanophyceae	--	--	--			+	
OTU 070	Photot.	Prok.	Cyanophyta	Cyanophyceae	--	--	--			+	
OTU 301	Photot.	Prok.	Cyanophyta	Cyanophyceae	--	--	--			+	
OTU 281	Photot.	Prok.	Cyanophyta	Cyanophyceae	--	--	--			+	
OTU 649	Photot.	Prok.	Cyanophyta	Cyanophyceae	--	--	--			+	
OTU 650	Photot.	Prok.	Cyanophyta	Cyanophyceae	--	--	--			+	
OTU 115	Photot.	Prok.	Cyanophyta	Cyanophyceae	--	--	--			+	
OTU 644	Photot.	Prok.	Cyanophyta	Cyanophyceae	--	--	--			+	
OTU 056	Photot.	Prok.	Cyanophyta	Cyanophyceae	--	--	--			+	
OTU 075	Photot.	Prok.	Cyanophyta	Cyanophyceae	--	--	--			+	
OTU 363	Photot.	Prok.	Cyanophyta	Cyanophyceae	--	--	--			+	
OTU 608	Photot.	Prok.	Cyanophyta	Cyanophyceae	--	--	--			+	
OTU 069	Photot.	Prok.	Cyanophyta	Cyanophyceae	--	--	--			+	
OTU 137	Photot.	Prok.	Cyanophyta	Cyanophyceae	--	--	--			+	
OTU 139	Photot.	Prok.	Cyanophyta	Cyanophyceae	--	--	--			+	
OTU 280	Photot.	Prok.	Cyanophyta	Cyanophyceae	--	--	--			+	
OTU 290	Photot.	Prok.	Cyanophyta	Cyanophyceae	--	--	--			+	
OTU 695	Photot.	Prok.	Cyanophyta	Cyanophyceae	--	--	--			+	
OTU 096	Photot.	Prok.	Cyanophyta	Cyanophyceae	--	--	--			+	
OTU 273	Photot.	Prok.	Cyanophyta	Cyanophyceae	--	--	--			+	
OTU 632	Photot.	Prok.	Cyanophyta	Cyanophyceae	--	--	--			+	
OTU 065	Photot.	Prok.	Cyanophyta	Cyanophyceae	--	--	--			+	
OTU 133	Photot.	Prok.	Cyanophyta	Cyanophyceae	--	--	--			+	
OTU 078	Photot.	Prok.	Cyanophyta	Cyanophyceae	--	--	--			+	
OTU 113	Photot.	Prok.	Cyanophyta	Cyanophyceae	--	--	--			+	
OTU 160	Photot.	Prok.	Cyanophyta	Cyanophyceae	--	--	--			+	
OTU 289	Photot.	Prok.	Cyanophyta	Cyanophyceae	--	--	--			+	
OTU 291	Photot.	Prok.	Cyanophyta	Cyanophyceae	--	--	--			+	
OTU 366	Photot.	Prok.	Cyanophyta	Cyanophyceae	--	--	--		+		
OTU 653	Photot.	Prok.	Cyanophyta	Cyanophyceae	--	--	--		+		
OTU 450	Photot.	Prok.	Cyanophyta	Cyanophyceae	--	--	--		+		
OTU 636	Photot.	Prok.	Cyanophyta	Cyanophyceae	--	--	--		+		
OTU 630	Photot.	Prok.	Cyanophyta	Cyanophyceae	--	--	--		+		
OTU 643	Photot.	Prok.	Cyanophyta	Cyanophyceae	--	--	--		+		
OTU 229	Photot.	Prok.	Cyanophyta	Cyanophyceae	--	--	--		+		
OTU 308	Photot.	Prok.	Cyanophyta	Cyanophyceae	--	--	--		+		
OTU 587	Photot.	Prok.	Cyanophyta	Cyanophyceae	--	--	--		+		
OTU 123	Photot.	Prok.	Cyanophyta	Cyanophyceae	--	--	--		+		
OTU 310	Photot.	Prok.	Cyanophyta	Cyanophyceae	--	--	--		+		

Identifier	Function	Domain	Phyla	Class	Order	Family (Suborder)	Genus	FL01	FL02	JP07	GM14
OTU 094	Photot.	Prok.	Cyanophyta	Cyanophyceae	--	--	--		+		
OTU 579	Photot.	Prok.	Cyanophyta	Cyanophyceae	--	--	--		+		
OTU 042	Photot.	Prok.	Cyanophyta	Cyanophyceae	--	--	--		+		
OTU 305	Photot.	Prok.	Cyanophyta	Cyanophyceae	--	--	--		+		
OTU 671	Photot.	Prok.	Cyanophyta	Cyanophyceae	--	--	--		+		
OTU 700	Photot.	Prok.	Cyanophyta	Cyanophyceae	--	--	--		+		
OTU 263	Photot.	Prok.	Cyanophyta	Cyanophyceae	--	--	--		+		
OTU 269	Photot.	Prok.	Cyanophyta	Cyanophyceae	--	--	--		+		
OTU 623	Photot.	Prok.	Cyanophyta	Cyanophyceae	--	--	--		+		
OTU 012	Photot.	Prok.	Cyanophyta	Cyanophyceae	--	--	--		+		
OTU 395	Photot.	Prok.	Cyanophyta	Cyanophyceae	--	--	--		+		
OTU 692	Photot.	Prok.	Cyanophyta	Cyanophyceae	--	--	--		+		
OTU 251	Photot.	Prok.	Cyanophyta	Cyanophyceae	--	--	--		+		
OTU 109	Photot.	Prok.	Cyanophyta	Cyanophyceae	--	--	--		+		
OTU 458	Photot.	Prok.	Cyanophyta	Cyanophyceae	--	--	--		+		
OTU 459	Photot.	Prok.	Cyanophyta	Cyanophyceae	--	--	--		+		
OTU 447	Photot.	Prok.	Cyanophyta	Cyanophyceae	--	--	--		+		
OTU 527	Photot.	Prok.	Cyanophyta	Cyanophyceae	--	--	--		+		
OTU 455	Photot.	Prok.	Cyanophyta	Cyanophyceae	--	--	--		+		
OTU 756	Photot.	Prok.	Cyanophyta	Cyanophyceae	--	--	--		+		
OTU 416	Photot.	Prok.	Cyanophyta	Cyanophyceae	--	--	--		+		
OTU 543	Photot.	Prok.	Cyanophyta	Cyanophyceae	--	--	--		+		
OTU 532	Photot.	Prok.	Cyanophyta	Cyanophyceae	--	--	--		+		
OTU 206	Photot.	Prok.	Cyanophyta	Cyanophyceae	--	--	--		+		
OTU 658	Photot.	Prok.	Cyanophyta	Cyanophyceae	Unclassified	Unclassified	Acaryochloris marina			+	
OTU 371	Heterot.	Prok.	--	--	--	--	--		+	+	
OTU 533	Heterot.	Prok.	--	--	--	--	--	+			+
OTU 112	Heterot.	Prok.	--	--	--	--	--				+
OTU 607	Heterot.	Prok.	--	--	--	--	--				+
OTU 713	Heterot.	Prok.	--	--	--	--	--				+
OTU 584	Heterot.	Prok.	--	--	--	--	--				+
OTU 086	Heterot.	Prok.	--	--	--	--	--				+
OTU 265	Heterot.	Prok.	--	--	--	--	--				+
OTU 800	Heterot.	Prok.	--	--	--	--	--				+
OTU 415	Heterot.	Prok.	--	--	--	--	--			+	
OTU 165	Heterot.	Prok.	--	--	--	--	--			+	
OTU 479	Heterot.	Prok.	--	--	--	--	--			+	
OTU 617	Heterot.	Prok.	--	--	--	--	--			+	
OTU 271	Heterot.	Prok.	--	--	--	--	--			+	
OTU 731	Heterot.	Prok.	--	--	--	--	--			+	
OTU 569	Heterot.	Prok.	--	--	--	--	--			+	
OTU 652	Heterot.	Prok.	--	--	--	--	--			+	
OTU 560	Heterot.	Prok.	--	--	--	--	--			+	
OTU 361	Heterot.	Prok.	--	--	--	--	--			+	
OTU 293	Heterot.	Prok.	--	--	--	--	--			+	
OTU 101	Heterot.	Prok.	--	--	--	--	--		+		
OTU 163	Heterot.	Prok.	--	--	--	--	--		+		
OTU 702	Heterot.	Prok.	--	--	--	--	--		+		
OTU 548	Heterot.	Prok.	--	--	--	--	--		+		
OTU 186	Heterot.	Prok.	--	--	--	--	--		+		
OTU 741	Heterot.	Prok.	--	--	--	--	--		+		
OTU 117	Heterot.	Prok.	Bacteroidetes	--	--	--	--		+	+	
OTU 699	Heterot.	Prok.	Bacteroidetes	--	--	--	--		+	+	
OTU 192	Heterot.	Prok.	Bacteroidetes	--	--	--	--		+	+	
OTU 718	Heterot.	Prok.	Bacteroidetes	--	--	--	--		+	+	
OTU 080	Heterot.	Prok.	Bacteroidetes	--	--	--	--		+	+	
OTU 513	Heterot.	Prok.	Bacteroidetes	--	--	--	--		+	+	
OTU 549	Heterot.	Prok.	Bacteroidetes	--	--	--	--		+	+	
OTU 224	Heterot.	Prok.	Bacteroidetes	--	--	--	--		+	+	
OTU 446	Heterot.	Prok.	Bacteroidetes	--	--	--	--		+	+	
OTU 350	Heterot.	Prok.	Bacteroidetes	--	--	--	--		+	+	
OTU 765	Heterot.	Prok.	Bacteroidetes	--	--	--	--		+	+	
OTU 199	Heterot.	Prok.	Bacteroidetes	--	--	--	--		+	+	
OTU 751	Heterot.	Prok.	Bacteroidetes	--	--	--	--		+	+	
OTU 245	Heterot.	Prok.	Bacteroidetes	--	--	--	--	+			
OTU 060	Heterot.	Prok.	Bacteroidetes	--	--	--	--				+

Identifier	Function	Domain	Phyla	Class	Order	Family (Suborder)	Genus	FL01	FL02	JP07	GM14
OTU 110	Heterot.	Prok.	Bacteroidetes	--	--	--	--				+
OTU 321	Heterot.	Prok.	Bacteroidetes	--	--	--	--				+
OTU 005	Heterot.	Prok.	Bacteroidetes	--	--	--	--				+
OTU 230	Heterot.	Prok.	Bacteroidetes	--	--	--	--				+
OTU 125	Heterot.	Prok.	Bacteroidetes	--	--	--	--			+	
OTU 347	Heterot.	Prok.	Bacteroidetes	--	--	--	--			+	
OTU 166	Heterot.	Prok.	Bacteroidetes	--	--	--	--			+	
OTU 769	Heterot.	Prok.	Bacteroidetes	--	--	--	--			+	
OTU 464	Heterot.	Prok.	Bacteroidetes	--	--	--	--			+	
OTU 782	Heterot.	Prok.	Bacteroidetes	--	--	--	--			+	
OTU 387	Heterot.	Prok.	Bacteroidetes	--	--	--	--			+	
OTU 188	Heterot.	Prok.	Bacteroidetes	--	--	--	--			+	
OTU 772	Heterot.	Prok.	Bacteroidetes	--	--	--	--			+	
OTU 784	Heterot.	Prok.	Bacteroidetes	--	--	--	--			+	
OTU 778	Heterot.	Prok.	Bacteroidetes	--	--	--	--			+	
OTU 011	Heterot.	Prok.	Bacteroidetes	--	--	--	--			+	
OTU 785	Heterot.	Prok.	Bacteroidetes	--	--	--	--			+	
OTU 449	Heterot.	Prok.	Bacteroidetes	--	--	--	--			+	
OTU 257	Heterot.	Prok.	Bacteroidetes	--	--	--	--			+	
OTU 443	Heterot.	Prok.	Bacteroidetes	--	--	--	--			+	
OTU 022	Heterot.	Prok.	Bacteroidetes	--	--	--	--			+	
OTU 423	Heterot.	Prok.	Bacteroidetes	--	--	--	--			+	
OTU 473	Heterot.	Prok.	Bacteroidetes	--	--	--	--			+	
OTU 442	Heterot.	Prok.	Bacteroidetes	--	--	--	--			+	
OTU 388	Heterot.	Prok.	Bacteroidetes	--	--	--	--			+	
OTU 427	Heterot.	Prok.	Bacteroidetes	--	--	--	--			+	
OTU 727	Heterot.	Prok.	Bacteroidetes	--	--	--	--			+	
OTU 657	Heterot.	Prok.	Bacteroidetes	--	--	--	--			+	
OTU 629	Heterot.	Prok.	Bacteroidetes	--	--	--	--			+	
OTU 035	Heterot.	Prok.	Bacteroidetes	--	--	--	--			+	
OTU 400	Heterot.	Prok.	Bacteroidetes	--	--	--	--			+	
OTU 232	Heterot.	Prok.	Bacteroidetes	--	--	--	--			+	
OTU 033	Heterot.	Prok.	Bacteroidetes	--	--	--	--			+	
OTU 262	Heterot.	Prok.	Bacteroidetes	--	--	--	--			+	
OTU 664	Heterot.	Prok.	Bacteroidetes	--	--	--	--			+	
OTU 028	Heterot.	Prok.	Bacteroidetes	--	--	--	--			+	
OTU 001	Heterot.	Prok.	Bacteroidetes	--	--	--	--			+	
OTU 053	Heterot.	Prok.	Bacteroidetes	--	--	--	--			+	
OTU 084	Heterot.	Prok.	Bacteroidetes	--	--	--	--			+	
OTU 719	Heterot.	Prok.	Bacteroidetes	--	--	--	--			+	
OTU 660	Heterot.	Prok.	Bacteroidetes	--	--	--	--			+	
OTU 103	Heterot.	Prok.	Bacteroidetes	--	--	--	--			+	
OTU 249	Heterot.	Prok.	Bacteroidetes	--	--	--	--			+	
OTU 260	Heterot.	Prok.	Bacteroidetes	--	--	--	--			+	
OTU 243	Heterot.	Prok.	Bacteroidetes	--	--	--	--			+	
OTU 024	Heterot.	Prok.	Bacteroidetes	--	--	--	--			+	
OTU 553	Heterot.	Prok.	Bacteroidetes	--	--	--	--			+	
OTU 573	Heterot.	Prok.	Bacteroidetes	--	--	--	--			+	
OTU 677	Heterot.	Prok.	Bacteroidetes	--	--	--	--			+	
OTU 686	Heterot.	Prok.	Bacteroidetes	--	--	--	--			+	
OTU 726	Heterot.	Prok.	Bacteroidetes	--	--	--	--			+	
OTU 085	Heterot.	Prok.	Bacteroidetes	--	--	--	--			+	
OTU 121	Heterot.	Prok.	Bacteroidetes	--	--	--	--			+	
OTU 007	Heterot.	Prok.	Bacteroidetes	--	--	--	--			+	
OTU 079	Heterot.	Prok.	Bacteroidetes	--	--	--	--			+	
OTU 240	Heterot.	Prok.	Bacteroidetes	--	--	--	--			+	
OTU 303	Heterot.	Prok.	Bacteroidetes	--	--	--	--			+	
OTU 577	Heterot.	Prok.	Bacteroidetes	--	--	--	--			+	
OTU 705	Heterot.	Prok.	Bacteroidetes	--	--	--	--			+	
OTU 309	Heterot.	Prok.	Bacteroidetes	--	--	--	--			+	
OTU 233	Heterot.	Prok.	Bacteroidetes	--	--	--	--			+	
OTU 081	Heterot.	Prok.	Bacteroidetes	--	--	--	--			+	
OTU 640	Heterot.	Prok.	Bacteroidetes	--	--	--	--			+	
OTU 058	Heterot.	Prok.	Bacteroidetes	--	--	--	--			+	
OTU 157	Heterot.	Prok.	Bacteroidetes	--	--	--	--			+	
OTU 159	Heterot.	Prok.	Bacteroidetes	--	--	--	--			+	

Identifier	Function	Domain	Phyla	Class	Order	Family (Suborder)	Genus	FL01	FL02	JP07	GM14
OTU 162	Heterot.	Prok.	Bacteroidetes	--	--	--	--			+	
OTU 648	Heterot.	Prok.	Bacteroidetes	--	--	--	--			+	
OTU 068	Heterot.	Prok.	Bacteroidetes	--	--	--	--			+	
OTU 568	Heterot.	Prok.	Bacteroidetes	--	--	--	--			+	
OTU 019	Heterot.	Prok.	Bacteroidetes	--	--	--	--			+	
OTU 064	Heterot.	Prok.	Bacteroidetes	--	--	--	--			+	
OTU 158	Heterot.	Prok.	Bacteroidetes	--	--	--	--			+	
OTU 435	Heterot.	Prok.	Bacteroidetes	--	--	--	--			+	
OTU 105	Heterot.	Prok.	Bacteroidetes	--	--	--	--			+	
OTU 324	Heterot.	Prok.	Bacteroidetes	--	--	--	--			+	
OTU 365	Heterot.	Prok.	Bacteroidetes	--	--	--	--			+	
OTU 297	Heterot.	Prok.	Bacteroidetes	--	--	--	--			+	
OTU 319	Heterot.	Prok.	Bacteroidetes	--	--	--	--			+	
OTU 460	Heterot.	Prok.	Bacteroidetes	--	--	--	--			+	
OTU 062	Heterot.	Prok.	Bacteroidetes	--	--	--	--			+	
OTU 111	Heterot.	Prok.	Bacteroidetes	--	--	--	--			+	
OTU 645	Heterot.	Prok.	Bacteroidetes	--	--	--	--			+	
OTU 076	Heterot.	Prok.	Bacteroidetes	--	--	--	--		+		
OTU 638	Heterot.	Prok.	Bacteroidetes	--	--	--	--		+		
OTU 454	Heterot.	Prok.	Bacteroidetes	--	--	--	--		+		
OTU 639	Heterot.	Prok.	Bacteroidetes	--	--	--	--		+		
OTU 703	Heterot.	Prok.	Bacteroidetes	--	--	--	--		+		
OTU 156	Heterot.	Prok.	Bacteroidetes	--	--	--	--		+		
OTU 563	Heterot.	Prok.	Bacteroidetes	--	--	--	--		+		
OTU 562	Heterot.	Prok.	Bacteroidetes	--	--	--	--		+		
OTU 099	Heterot.	Prok.	Bacteroidetes	--	--	--	--		+		
OTU 322	Heterot.	Prok.	Bacteroidetes	--	--	--	--		+		
OTU 662	Heterot.	Prok.	Bacteroidetes	--	--	--	--		+		
OTU 074	Heterot.	Prok.	Bacteroidetes	--	--	--	--		+		
OTU 093	Heterot.	Prok.	Bacteroidetes	--	--	--	--		+		
OTU 665	Heterot.	Prok.	Bacteroidetes	--	--	--	--		+		
OTU 238	Heterot.	Prok.	Bacteroidetes	--	--	--	--		+		
OTU 278	Heterot.	Prok.	Bacteroidetes	--	--	--	--		+		
OTU 006	Heterot.	Prok.	Bacteroidetes	--	--	--	--		+		
OTU 264	Heterot.	Prok.	Bacteroidetes	--	--	--	--		+		
OTU 054	Heterot.	Prok.	Bacteroidetes	--	--	--	--		+		
OTU 469	Heterot.	Prok.	Bacteroidetes	--	--	--	--		+		
OTU 467	Heterot.	Prok.	Bacteroidetes	--	--	--	--		+		
OTU 008	Heterot.	Prok.	Bacteroidetes	--	--	--	--		+		
OTU 480	Heterot.	Prok.	Bacteroidetes	--	--	--	--		+		
OTU 615	Heterot.	Prok.	Bacteroidetes	--	--	--	--		+		
OTU 601	Heterot.	Prok.	Bacteroidetes	--	--	--	--		+		
OTU 490	Heterot.	Prok.	Bacteroidetes	--	--	--	--		+		
OTU 482	Heterot.	Prok.	Bacteroidetes	--	--	--	--		+		
OTU 797	Heterot.	Prok.	Bacteroidetes	--	--	--	--		+		
OTU 485	Heterot.	Prok.	Bacteroidetes	--	--	--	--		+		
OTU 344	Heterot.	Prok.	Bacteroidetes	--	--	--	--		+		
OTU 171	Heterot.	Prok.	Bacteroidetes	--	--	--	--		+		
OTU 342	Heterot.	Prok.	Bacteroidetes	--	--	--	--		+		
OTU 539	Heterot.	Prok.	Bacteroidetes	--	--	--	--		+		
OTU 768	Heterot.	Prok.	Bacteroidetes	--	--	--	--		+		
OTU 738	Heterot.	Prok.	Bacteroidetes	--	--	--	--		+		
OTU 502	Heterot.	Prok.	Bacteroidetes	--	--	--	--		+		
OTU 492	Heterot.	Prok.	Bacteroidetes	--	--	--	--		+		
OTU 386	Heterot.	Prok.	Firmicutes	--	--	--	--	+			
OTU 061	Heterot.	Prok.	Firmicutes	--	--	--	--		+		
OTU 312	Heterot.	Prok.	Firmicutes	--	--	--	--		+		
OTU 124	Heterot.	Prok.	Proteobacteria	--	--	--	--		+	+	
OTU 164	Heterot.	Prok.	Proteobacteria	--	--	--	--				+
OTU 496	Heterot.	Prok.	Proteobacteria	--	--	--	--			+	
OTU 767	Heterot.	Prok.	Proteobacteria	--	--	--	--			+	
OTU 448	Heterot.	Prok.	Proteobacteria	--	--	--	--			+	
OTU 799	Heterot.	Prok.	Proteobacteria	--	--	--	--			+	
OTU 779	Heterot.	Prok.	Proteobacteria	--	--	--	--			+	
OTU 583	Heterot.	Prok.	Proteobacteria	--	--	--	--			+	
OTU 734	Heterot.	Prok.	Proteobacteria	--	--	--	--			+	

Identifier	Function	Domain	Phyla	Class	Order	Family (Suborder)	Genus	FL01	FL02	JP07	GM14
OTU 083	Heterot.	Prok.	Proteobacteria	--	--	--	--			+	
OTU 465	Heterot.	Prok.	Proteobacteria	--	--	--	--			+	
OTU 016	Heterot.	Prok.	Proteobacteria	--	--	--	--			+	
OTU 475	Heterot.	Prok.	Proteobacteria	--	--	--	--			+	
OTU 706	Heterot.	Prok.	Proteobacteria	--	--	--	--			+	
OTU 613	Heterot.	Prok.	Proteobacteria	--	--	--	--			+	
OTU 642	Heterot.	Prok.	Proteobacteria	--	--	--	--			+	
OTU 294	Heterot.	Prok.	Proteobacteria	--	--	--	--			+	
OTU 302	Heterot.	Prok.	Proteobacteria	--	--	--	--			+	
OTU 055	Heterot.	Prok.	Proteobacteria	--	--	--	--		+		
